# Modular Characteristics and Mechanism of Action of Herbs for Endometriosis Treatment in Chinese Medicine: A Data Mining and Network Pharmacology–Based Identification

**DOI:** 10.3389/fphar.2020.00147

**Published:** 2020-03-06

**Authors:** Weilin Zheng, Jiayi Wu, Jiangyong Gu, Heng Weng, Jie Wang, Tao Wang, Xuefang Liang, Lixing Cao

**Affiliations:** ^1^The Second Clinical College of Guangzhou University of Chinese Medicine, Guangzhou, China; ^2^Guangzhou University of Chinese Medicine, Guangzhou, China; ^3^Department of Big Medical Data, Second Affiliated Hospital of Guangzhou University of Chinese Medicine, Guangzhou, China; ^4^Department of Gynecology, Second Affiliated Hospital of Guangzhou University of Chinese Medicine, Guangzhou, China; ^5^Team of Application of Chinese Medicine in Perioperative Period, Second Affiliated Hospital of Guangzhou University of Chinese Medicine, Guangzhou, China

**Keywords:** medicinal herb, data mining, network analysis, bioinformatics, network pharmacology, endometriosis

## Abstract

Endometriosis is a common benign disease in women of reproductive age. It has been defined as a disorder characterized by inflammation, compromised immunity, hormone dependence, and neuroangiogenesis. Unfortunately, the mechanisms of endometriosis have not yet been fully elucidated, and available treatment methods are currently limited. The discovery of new therapeutic drugs and improvements in existing treatment schemes remain the focus of research initiatives. Chinese medicine can improve the symptoms associated with endometriosis. Many Chinese herbal medicines could exert antiendometriosis effects *via* comprehensive interactions with multiple targets. However, these interactions have not been defined. This study used association rule mining and systems pharmacology to discover a method by which potential antiendometriosis herbs can be investigated. We analyzed various combinations and mechanisms of action of medicinal herbs to establish molecular networks showing interactions with multiple targets. The results showed that endometriosis treatment in Chinese medicine is mainly based on methods of supplementation with blood-activating herbs and strengthening qi. Furthermore, we used network pharmacology to analyze the main herbs that facilitate the decoding of multiscale mechanisms of the herbal compounds. We found that Chinese medicine could affect the development of endometriosis by regulating inflammation, immunity, angiogenesis, and other clusters of processes identified by Gene Ontology (GO) and Kyoto Encyclopedia of Genes and Genomes (KEGG) pathway analyses. The antiendometriosis effect of Chinese medicine occurs mainly through nervous system–associated pathways, such as the serotonergic synapse, the neurotrophin signaling pathway, and dopaminergic synapse, among others, to reduce pain. Chinese medicine could also regulate VEGF signaling, toll-like reporter signaling, NF-κB signaling, MAPK signaling, PI3K-Akt signaling, and the HIF-1 signaling pathway, among others. Synergies often exist in herb pairs and herbal prescriptions. In conclusion, we identified some important targets, target pairs, and regulatory networks, using bioinformatics and data mining. The combination of data mining and network pharmacology may offer an efficient method for drug discovery and development from herbal medicines.

## Introduction

Endometriosis is a common benign condition in women of reproductive age, which is characterized by inflammation, compromised immunity, hormone dependence, and neuroangiogenesis, etc. (Rogers et al., 2009). The main symptoms of endometriosis include dysmenorrhea, endometriosis-associated pain, and infertility (Brown and Farquhar, 2014; Bedaiwy et al., 2017). In addition to surgical treatment, conventional drug therapies includes nonsteroidal anti-inflammatory drugs (NSAIDs) and other drugs, such as androgens, aromatase inhibitors, selective progesterone receptor modulators, oral contraceptives, and gonadotropin releasing hormone (GnRH) agonists (Mihalyi et al., 2006; Dunselman et al., 2014; Legendre et al., 2018). Although these drugs play a certain therapeutic role, the main effect is only to relieve symptoms, delay recurrence, and bring certain side effects. Chinese medicine has attracted extensive attention for its ability to treat complex diseases due to its moderate treatment effect and lower side effect. Given the complex pathogenesis of the condition and limited treatment effect, patients with endometriosis often explore Chinese medicines to treat the primary lesions or control the symptoms (Flower et al., 2012). Chinese medicine has significant advantages in treating gynecological disorders such as endometriosis associated dysmenorrhea, chronic pelvic pain, abnormal uterine bleeding, and infertility (Zhou et al., 2019). However, due to the differences in clinical experience and the complexity of Chinese medicine, prescriptions differ greatly from each other. In addition, current Traditional Chinese Medicine (TCM)–related research is mainly focused on experience summarization (Su et al., 2014). These lack the in-depth study of the rules and mechanisms of medication in prescriptions. We urgently need to summarize the rules for the administration of Chinese medicine and analyze the mechanism of action of the herbs, in order to provide the basis for the optimization of drug use and the screening of new compounds.

Data mining approaches and network pharmacology have been utilized to discover the underlying pathogenesis of the condition (Ren et al., 2019). Data mining approaches can analysis the frequency of herbal medications, formula ontology, and changes in formula patterns derived from a knowledge graph (Leem et al., 2018; You et al., 2019). Network pharmacology is a holistic approach to understanding the function and behavior of a biological system, at the systems level in the context of biological networks. Network pharmacology, when combined with traditional pharmacology, can predict the target profiles and pharmacological actions of herbal compounds to reveal drug-gene-disease comodule associations, screen synergistic multicompounds, and elucidate herbal ingredients with their related properties and compound-target and target-disease relationships (Zeng et al., 2017).

In this study, data mining was used to analyze the application rules of TCM in the treatment of endometriosis. We also proposed a computational systems pharmacology method and statistical analysis to determine the associated molecular mechanisms. We analyzed an endometriosis biomarker network and identified biologically relevant functional modules of Chinese medicine targets in endometriosis. These findings would provide a better understanding of the mechanism of multitarget regulation of endometriosis in Chinese medicine and shed light on the screening of core compounds. This in turn has facilitated the discovery of novel effective drugs.

## Materials and Methods

### Workflow of Data Mining and Network Pharmacology Approach

**Figure 1:**
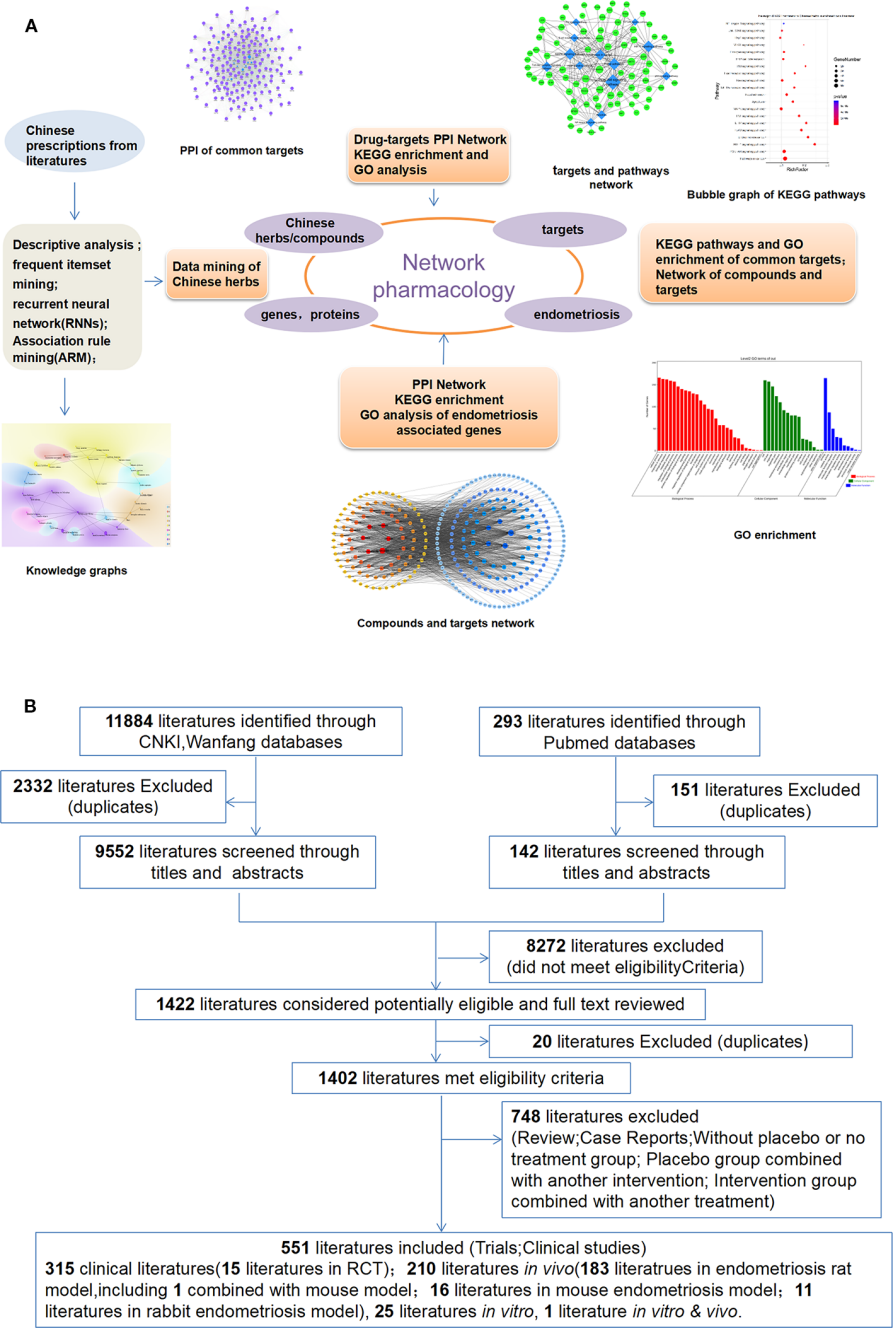
Workflow of the systems pharmacology approach. **(A)** Method (1) Endometriosis-associated genes were integrated from three different sources: the Genecard database, PubMed database, Genbank, and the Online Mendelian Inheritance in Man (OMIM) database. (2) Construction of protein-protein interaction (PPI) topological modules. Gene Ontology (GO) and pathway enrichment functional analyses were conducted for the endometriosis and Chinese medicine targets modules. (3) Disease-related conventional medication targets from the Therapeutic Target Database (TTD) and DrugBank databases, guidelines, and published literature. (4) KEGG and GO analyses of the targets of major compounds. (5) Analysis of the targets of endometriosis and pathways that Chinese herbs could regulate. **(B)** Workflow of literatures search for data mining.

The research framework of this study is presented in **Figure 1A**. It is summarized as follows: (1) Base datasets of herbal medicines with antiendometriosis effects were constructed. These datasets of Chinese herbal medicines were screened from the literature. (2) The main ingredients and targets were obtained. The targets of endometriosis treatment were derived from the Genecard dataset, National Center for Biotechnology Information (NCBI), Genbank, and the Online Mendelian Inheritance in Man (OMIM) databases, disease-related drug targets from the Therapeutic Target Database (TTD), DrugBank databases, the search tool for interactions of chemicals (STITCH) dataset (http://stitch.embl.de/) (Szklarczyk et al., 2016), etc. The Gene Ontology (GO) database, Kyoto Encyclopedia of Genes and Genomes (KEGG), and the Database for Annotation, Visualization, and Integrated Discovery (DAVID) were all explored. (3) Molecular networks and pathways were analyzed, mutual target proteins were mined and the regulatory network was explored. (4) The network of targets for the condition was constructed. Cytoscape, an open source software project for the integration of biomolecular interaction networks with high-throughput expression data, was applied and other molecular states were combined in a unified conceptual framework (Shannon et al., 2003).

### Search Methods

We conducted an electronic search of the Chinese and English databases from their inception to 31/12/2018. We searched the Chinese literatures in China National Knowledge Infrastructure (CNKI) database and WanFang database. The English databases included Embase and the PubMed database. All types and formulations of herbal medicines were considered, including extracts, decoctions, pills, and natural compounds. The outcomes of clinical studies were not considered.

The following search terms were used either individually or in combination: “Chinese medicine,” “traditional medicine,” “herbal medicine,” “oriental medicine,” “herb,” “plant,” “prescription,” “decoction,” and “endometriosis,” among others. All prescriptions for endometriosis treatment were selected and the drug records were complete. Formulations of pills, powders, and decoctions were all evaluated. The name of each herbal formula was extracted, as well as its composition of medicinal herbs, its origin (name of article or ancient literature), author, publication year, and internal/external application. Exclusion criteria included the following: missing prescription; the application of acupuncture, massage, or other external treatments. The method of literature searching is presented in **Figure 1B**.

### TCM and Compound Targets

The names of Chinese herb included the Chinese pinyin and full plant name with taxonomic validation (species name/scientific name). The validated information of herbs/plants names with taxonomic validation were collected from Kew Royal Botanic Garden (https://mpns.science.kew.org/mpns-portal/), the Plant List (http://www.theplantlist.org), and the Plants of the World Online(http://plantsoftheworldonline.org/). Chemical ingredients were compiled from the TCM Systems Pharmacology (TCMSP) Database (http://tcmspw.com/tcmsp.php), TCM Integrative Database (TCMID) (http://www.megabionet.org/tcmid/), universal natural product database (UNPD) (Gu et al., 2013), TCM-MeSH(http://mesh.tcm.microbioinformatics.org/), STITCH database (http://stitch.embl.de), and DrugBank (https://www.drugbank.ca). Finally, the structures of relevant natural compounds in the medicinal plants were retrieved from the NCBI Pubchem dataset (https://www.ncbi.nlm.nih.gov/pccompound/).

### Active Ingredients Screening

The absorption, distribution, metabolism, excretion, and toxicity (ADMET) modeling as a tool for rational drug design has significant effect in new drug discovery (Wang et al., 2015). The druggability of each candidate was analyzed according to its oral bioavailability (OB) and drug-likeness (DL) indices, as recommended by the TCMSP database. High OB values is often an important consideration for the development of bioactive molecules as therapeutic agents. DL evaluation is used in drug design to evaluate whether a compound is chemically suitable for use as a drug, and how drug-like a molecule is with respect to parameters that affect its pharmacodynamic and pharmacokinetic profiles, which ultimately impact its absorption, distribution, metabolism, and excretion properties. The molecules with OB ≥ 25% and DL ≥ 0.18 could be considered to exhibit relatively better pharmacological properties. We further selected the major compounds based on literatures to identify the potential therapeutic effect. Although some compounds, such as essential oils, have lower DL values, they are selected because the effect had been experimental verification.

### Endometriosis-Related Targets and Endometriosis-Associated Drugs Targets

We also collected targets of endometriosis. To obtain the most reliable results, the following steps were taken: (A) endometriosis-associated genes were integrated from three different sources: the Genecard database, Malacards database (https://www.malacards.org/), literature on PubMed, Genbank, and the OMIM database (http://www.omim.org/). (B) Disease-related drug targets were compiled from the TTD (http://bidd.nus.edu.sg/group/cjttd/), DrugBank databases (http://www.drugbank.ca/, version: 3.0), PharmGKB (https://www.pharmgkb.org), The Drug-Gene Interaction database (DGIdb) (http://dgidb.org/search_interactions), as well as the European Society of Human Reproduction and Embryology (ESHRE) guidelines (Dunselman et al., 2014). We used those drug-target interactions to determine the treatments for which the targets were human genes/proteins. (C) Associated targets and structures of human proteins were determined. The targets and proteins were researched in the UniProt (https://www.uniprot.org).

### Data Analysis

#### Data Mining Analysis

We used “A Framework for Automated Knowledge Graph Construction Towards Traditional Chinese Medicine” developed by the Second Affiliated Hospital of Guangzhou University of Chinese Medicine to analyze the Chinese herbs for endometriosis in the literature (Weng et al., 2017). In the early stage of data set, we used more than 1,100 ancient literatures in the “Chinese Medical Code” dataset to extract knowledge of Chinese medicine. And we further constructed a large-scale knowledge graph of TCM. It contains 13 first-class knowledge classifications, 116 second-class knowledge classifications, more than 59,000 core knowledge elements, more than 0.5 million expand core knowledge elements, and more than 215,000 knowledge lists of herbal medicine and prescriptions. Then we used the recurrent neural network (RNN) model (Cho et al., 2014) to establish the generalized Chinese medicine knowledge meta-semantic representation. And we used fine-tune's transfer learning method, a way of machine learning, to learn the core knowledge elements of gynecological disease in dataset, and further obtained Chinese medicine knowledge meta-semantic representation vector. Finally, the hierarchical clustering results network and semantic representation could be visualized. Based on the above research, we selected relevant prescriptions in endometriosis treatment for analysis.

A. Descriptive analysis and frequent itemset mining: the frequency and pattern was used to calculate the type and frequency of each Chinese medicinal compound (Chen et al., 2008). The TCM paired drugs discovered by frequent itemset analysis (Feng et al., 2006).

B. Knowledge graph and RNNs: this was conducted using a hierarchical clustering algorithm, according to the nature, flavor, meridian tropism, and main efficacy of medicinal compounds. We established the knowledge graph of RNNs. RNNs are temporal-based neural networks that capture long sequences of inputs using the internal memory which has been used in medical research (Zhang Y. et al., 2019).

C. Association rule mining (ARM): The compatibility rules of the couplet medicinal/group medicinal were extracted according to association rule analysis. The strength of the association rule was measured in terms of its support, confidence, and lift. ARM was used to demonstrate the prescription (Chen et al., 2008).

ARM and RNNs analysis could further reflect the compatibility rules. Based on the above, combined with the results of previous literature reviews, further screening of TCMs, drug pairs, and compound pharmacological analyses could be performed.

#### Construction of Gene Enrichment Analysis

To better understand the processes associated with endometriosis and Chinese medicine treatment, we performed GO terms and KEGG pathway enrichment analyses. The GO enrichment analysis provides three structured networks of defined terms to describe gene attributes. Enriched GO terms are classified according to biological process (BP), molecular function (MF), and cellular component (CC). The KEGG (http://www.genome.jp/kegg/) is a database for large-scale systematic analysis of molecular interaction networks of genes or proteins (Kanehisa and Goto, 2000). The DAVID bioinformatics resources consist of an integrated biological knowledge base and analytic tools, aimed at systematically extracting biological meaning from large gene or protein lists. We used the web-based search engine, DAVID, to determine over-represented GO terms and KEGG pathways with thresholds of an enrichment score > 2, count > 5, and *P* < 0.05 and analyzed endometriosis-related pathways and GO terms. Venn diagram and bubble graphs of were performed using the OmicShare tools, a free online platform for data analysis (http://www.omicshare.com/tools). Significant pathway terms of KEGG were mapped into a bubble graph. The big and higher bubbles represent those highly significantly enriched pathway terms.

#### Protein-Protein Interaction Network Analysis

Protein-protein interaction (PPI) networks included information on the biological processes and molecular functions of cells (Vidal et al., 2011). We used the online search tool for recurring instances of neighboring genes (STRING, Version 9.1) (http://www.string-db.org) to predict the interactions. The Cytoscape software 3.7.2 (http://cytoscape.org/) was used to visualize networks. To identify crucial relationships in the PPI network, potentially overlapping modules that were densely connected were subsequently identified using Molecular Complex Detection (MCODE) plugin in the Cytoscape program. The MCODE plugin was used to re‐analyze the clusters among the network according to the k‐core = 2. A value of *P* < 0.01 was considered the significant threshold. And Cytoscape plugin *cytohubba* (Chin et al., 2014) was used to identify central elements of biological networks.

#### Network Construction of KEGG Pathway

To better analysis the holistic mechanism of Chinese herbs in endometriosis treatment, the subnetworks pathway was compiled by following the procedures: All targets of Chinese herbs in endometriosis treatment were submitted to an online tool KEGG Search Pathway (https://www.genome.jp/kegg/tool/map_pathway1.html). Based on the mechanism of endometriosis, multiple pathways were integrated and overlapped according to cross-talk targets in these maps. Based on the cross-talk of the pathways, we further constructed a targets-pathways network of Chinese medicine treatment.

#### The Connectiviy Map Analysis

The Connectivity Map (CMap) is an online pharmacogenomic database using gene-expression signatures from cultured cells treated individually with various chemicals to connect small molecules, genes, and disease(Chin et al., 2014). The expression profiles GSE25628 were downloaded from the Gene Expression Omnibus (GEO) database of patients both with and without endometriosis. The GSE25628 (https://www.ncbi.nlm.nih.gov/geo/query/acc.cgi?acc=GSE25628) gene expression profiles comprised 22 samples (GPL571 Affymetrix Human Genome U133A 2.0 Array), with eight ectopic samples and eight eutopic endometrium samples from several affected women in the proliferative phase, and normal health donors as the control. The limma package in the R software was used to compare the differentially expressed genes (DEGs) between eutopic endometrium samples group and ectopic endometrium samples group, and the DEGs between eutopic endometrium samples group and endometrium in normal health women group. In addition, these two sets of DEG profiles were used to predict compounds by the Connectivity Map O2 (CMap) (https://portals.broadinstitute.org/cmap/, Update September 12, 2017) (Lamb et al., 2006; Lamb, 2007). The representing similarity was calculated, ranging from −1 to 1. A positive connectivity score indicated that the drug was able to induce the input signature in human cell lines. The negative connectivity score was indicated potential therapeutic value. After rank ordering of all instances, the connectivity score of various instances were filtered according to the *P-*value < (0.05) to determine the natural compounds for further consideration.

#### Network Construction

We established a network analysis of the relevant compounds targets. The components of the target networks for the herbs were constructed using the Cytoscape software 3.7.2. The nodes in each network were evaluated based on three indices: degree, node betweenness, and node closeness. Degree indicated the number of edges between a single node and other nodes in a network. Node closeness represented the inverse of the sum of the distance from one node to other nodes. The importance of a node in a network was indicated by the values of these indices, with higher values indicating greater importance.

## Results

### Chinese Medicines Commonly Used for Endometriosis

In the past, we have studied the prescriptions of Chinese medicine for endometriosis through literature monographs. A total of 551 literatures were screened: 513 literatures in Chinese databases (including CNKI and WanFang databases) and 38 literatures from the PubMed and Embase database. Among those screened, 315 literatures were clinical studies, and 234 literatures were experimental studies (25 literatures were *in vitro*, 210 literatures were *in vivo*, and one literature was both *in vitro* and *in vivo*). The major TCM literatures were clinical observational studies. Fifteen literatures were clinical randomized controlled studies. Some literatures contain two or three prescriptions according to different syndromes in TCM. A total of 615 medicinal prescriptions were selected from the literature screened (**Figure 1B**). The most published prescriptions included those for *Guizhi Fuling Wan (GFW), Jiawei Sanleng Wan, Neiyi Fang*, and *Shaofu Zuyu* decoction.

**Figure 2:**
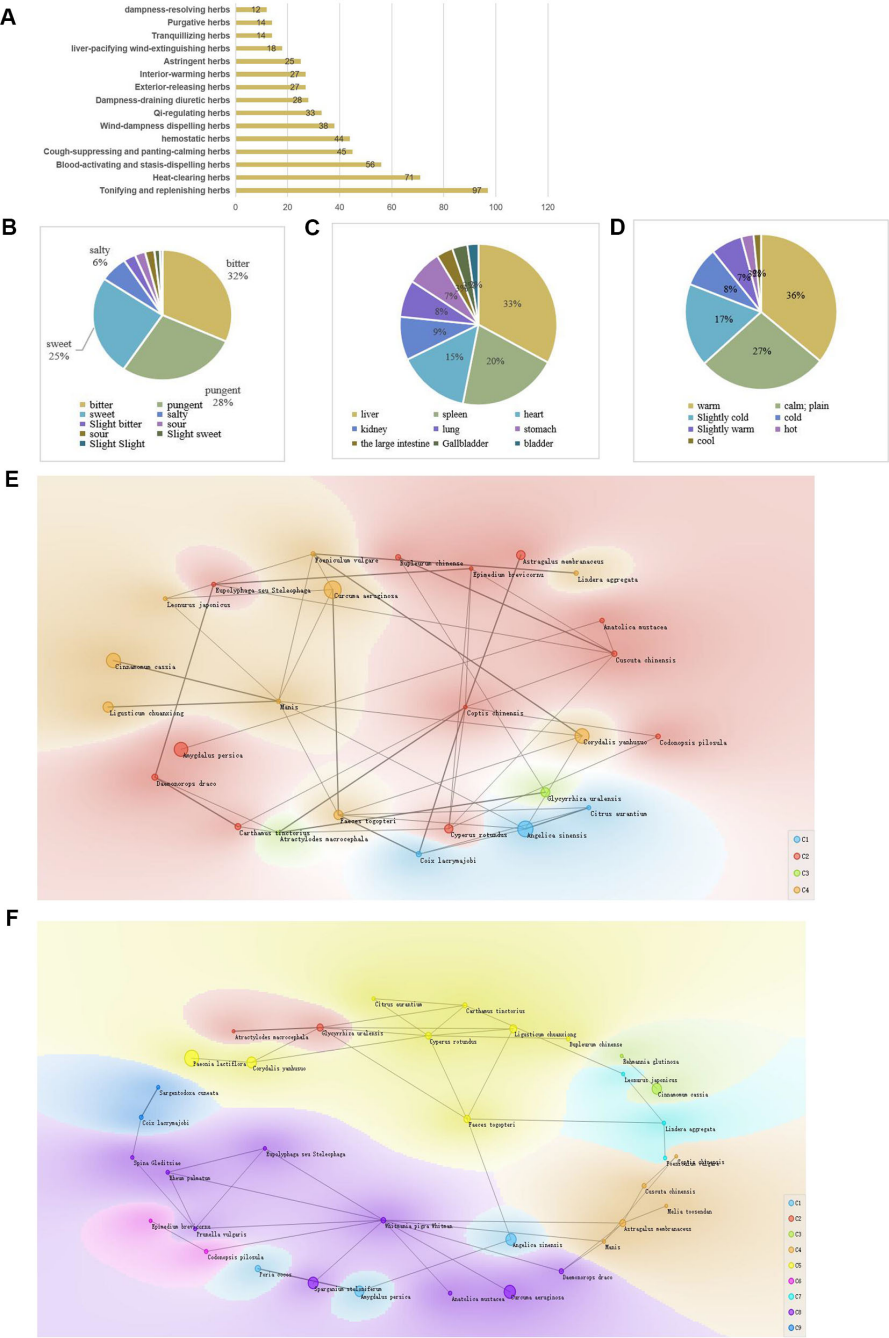
Data mining of Chinese herbs from literature. **(A)** Ratio of herbs of different classifications. Traditional Chinese medicine includes tonics, heat-clearing medicines, and blood-activating and stasis-removing medicines. **(B–D)** Ratios of the taste and action of Chinese herbs. Among the Chinese herbs evaluated, most were used as warm drugs, mainly targeting the liver and spleen. **(E, F)** Knowledge Graphs of major Chinese herbs. The size of a node represents the frequency of Traditional Chinese medicine in our gynecological dataset of Chinese medicine. The node represents the knowledge element, Chinese medicine. Cosine distance of the knowledge meta-semantic representation vector, a feature of clustering, was evaluated. Based on the association rules, frequent itemsets and recurrent neural networks (RNNs), the knowledge graphs were constructed, which were divided into four clusters and nine clusters, respectively. The knowledge graphs may provide the basis for the discovery of relevant prescriptions. Cluster analysis could also provide a reference for prescription guidelines.

The total 231 kinds of Chinese herbs were selected in literatures. The top 20 herbs were determined using frequency statistics and are presented in **Table 1**. The validated information of major herbs, including location, used part, famliy, genus are showed in **Supplementary Table 1**. We analyzed the classification, medicinal properties, and medicinal taste of all common drugs for endometriosis. Analysis from the prescriptions for endometriosis indicated a higher proportion and frequency of deficiency-tonifying herbs, blood-activating herbs, dampness-draining diuretic herbs, heat-clearing herbs, and digestant herbs (**Figure 2A**). *Curcuma phaeocaulis* Valeton (Ezhu), *Sparganium stoloniferum* (Buch.-Ham. ex Graebn.) Buch.-Ham. ex Juz.[Typhaceae] (Sanleng), and *Corydalis yanhusuo* (Y.H.Chou & Chun C.Hsu) W.T.Wang ex Z.Y.Su & C.Y.Wu(Yanhusuo) are both effective in promoting blood circulation to alleviate pain and activate qi to resolve stagnation in Chinese theory. The treatment of endometriosis with basic theory of Chinese medicine has always been based on promoting blood circulation to minimize blood stasis and alleviate pain.

**Table 1:** Frequency, module, and meridian tropism of the top 20 medicinal herbs.

No.	TCM Name(Chinese Pinyin)	Species Name/Scientific Name	Family	Genus	Properties	Meridians	Effect	Frequency
1	Ezhu	1*.Curcuma phaeocaulis* Valeton[Zingiberaceae] 2*.Curcuma zedoaria (Christm.)* Roscoe [Zingiberaceae]	Zingiberaceae Martinov	*Curcuma L*.	Warm, Pungent, Bitter	Spleen, Liver	Treatment of mass in the abdomen, amenorrhea due to blood stasis, distension and pain.	226
2	Chishao	*Paeonia lactiflora* Pall.	Paeoniaceae Raf.	*Paeonia*	Minor cold, Bitter	Liver	Treatment of pain in the chest and coastal regions, amenorrhea, dysmenorrhea, mass formation in the abdomen, traumatic injuries.	218
3	Danggui	*Angelica sinensis* (Oliv.)Diels	Apiaceae* *Lindl.	*Angelica*.	Warm, Pungent, Sweet	Spleen, Liver, Heart	To nourish blood and regulate menstruation, quicken blood, relieve pain, moisten intestines and relieve constipation.	202
4	Sanleng	*Sparganium stoloniferum* (Buch.-Ham. ex Graebn.) Buch.-Ham. ex Juz.	Typhaceae Juss.	*Sparganium* *L*.	Mild, Bitter	Spleen, Liver	To break blood, move qi and relieve pain, disperse accumulation.	198
5	Yanhusuo	*Corydalis yanhusuo* (Y.H.Chou & Chun C.Hsu) W.T.Wang ex Z.Y.Su & C.Y.Wu [Papaveraceae]	Papaveraceae Juss.	*Corydalis* *DC*.	Warm, Pungent, Bitter	Spleen, Liver, Heart	For stagnation of vital energy or blood stasis resulting in headache, chest pain, hypochondriac pain, epigastric pain, abdominal pain, backache, arthralgia, dysmenorrhea or trauma.	185
6	Taoren	*Prunus persica* (L.) Batsch	Rosaceae	*Prunus L*.	Mild, Sweet, Bitter	Large Intestine, Liver, Heart	To regulate blood and dispel stasis, moisten intestines and free stool.	177
7	Danshen	*Salvia miltiorrhiza* Bunge [Lamiaceae]	Lamiaceae Martinov	*Salvia L*.	Minor cold, Bitter	Liver, Heart	To regulate blood and dispel stasis, regulate menstruation and relieve pain, nourish blood and quiet spirit, cool blood.	164
8	Chuanxiong	1.*Conioselinum anthriscoides* (H.Boissieu) Pimenov & Kljuykov [Apiaceae] *2.Ligusticum chuanxiong* *3.Conioselinum anthriscoides* ‘Chuanxiong' [Apiaceae]	Apiaceae	*Ligusticum*	Warm, Pungent	Liver, Cardiovascular, Gallbladder	To move qi and quicken blood, dispel wind and relieve pain.	125
9	Guizhi	*Cinnamomum cassia* (L.) J.Presl [Lauraceae]	Lauraceae	*Cinnamomum Schaeff*.	Warm, Pungent, Sweet	Lung, Bladder, Heart	To dissipate cold and resolve exterior, warm channels and free network vessels, promote yang and transform qi.	123
10	Puhaung	*Typha angustifolia L*. [Typhaceae]	Typhaceae	*Typha L*.	Mild, Sweet	Liver, Heart	To lower cholesterol, cool blood and stanch bleeding, quicken blood and dispel stasis.	112
11	Wulingzhi *	*Faeces Togopteri*	Trogopterus xanthipes Milne	Edwards	Warm, Sweet, Bitter, Salty	Liver	To quicken blood and relieve pain, transform stasis and stanch bleeding, disperse accumulation and resolve toxin.	109
12	Huangqi	*Astragalus mongholicus* Bunge [Fabaceae]	Fabaceae Lindl.	*Astragalus L*.	Warm, Sweet	Lung, Spleen	To boost qi and secure exterior, disinhibit urine and draw toxin, expel pus, close sores and engender flesh.	104
13	Xiangfu	*Cyperus rotundus L*.	Cyperaceae Juss.	*Cyperus*.	Mild, Pungent, Slightly Sweet, Slightly Bitter	Spleen, Liver, Three End	To move qi and relieve depression, regulate menstruation and relieve pain.	99
14	Mudanpi	*Paeonia suffruticosa Andrews* [Paeoniaceae]	Paeoniaceae Raf.	*Paeonia L*.	Minor cold, Pungent, Bitter	Liver, Heart, Kidney	To clear heat and cool blood, quicken blood and dissipate stasis.	95
15	Fuling	*Wolfiporia extensa*(Peck) Ginns *Poria cocos*(Schw.)Wolf	Polyporaceae	*Poria Pers.ex Grag*	Mild, Sweet, Neutral	Spleen, Heart, Kidney	To disinhibit water and percolate damp,fortify spleen and quiet heart.	90
16	Gancao	*Glycyrrhiza uralensis* Fisch. ex DC. [Fabaceae]	Fabaceae Lindl	*Glycyrrhiza Tourn. ex L*.	Mild, Sweet	Lung, Spleen, Stomach, Heart	To supplement center and boost qi, relax tension and relieve pain	74
17	Shuizhi	*Whitmania pigra Whitman*	Gnathobdellida	Hirudinidae	Mild, Bitter, Salty	Liver	To clear heat and resolve toxin, disperse swelling and relieve pain.	74
18	Baishao	*Paeonia lactiflora* Pall.	Paeoniaceae Raf.	*Paeonia L*.	Minor cold, Sour, Bitter	Spleen, Liver	To calm liver and relieve pain, nourish blood and regulate menstruation, constrain yin and check sweating.	73
19	Moyao	*Commiphora myrrha* (T.Nees) Engl.	Burseraceae Kunth	*Commiphora Jacq*.	Mild, Pungent, Bitter	Spleen, Liver, Heart, Kidney	To quicken blood and relieve pain, disperse swelling and engender flesh.	67
20	Honghua	*Carthamus tinctorius* L.[Asteraceae]	Asteraceae Bercht. & J.Presl	*Carthamus L*.	Warm, Pungent	Liver, Heart	To quicken blood and free menstruation, dissipate stasis and relieve pain.	66

The characteristics of four properties, five tastes, and channel tropism were evaluated. The analysis of herbal properties is presented in **Figures 2B–D**. Chinese herbs that were considered warm and slight were given priority. The treatment of endometriosis is mainly based on invigorating the spleen, tonifying the liver to strengthen healthy qi, and supplementation with blood-activating herbs to eliminate pathogenic factors.

Frequent itemset mining is a crucial data mining task with numerous applications in discovery, including recommendation and classification, among others (Soltani and Akbarzadeh-T, 2014). By considering both the frequent itemsets and association rules, we inferred the relevant drug pairs and combinations, such as *S. stoloniferum* (Sanleng)*-C. phaeocaulis*(Ezhu); *C. phaeocaulis-Paeonia lactiflora* Pall(Chishao); and *C. yanhusuo* (Yanhusuo)*-Angelica sinensis (Oliv.)Diels* (Danggui), among others. The main roles of these herbs were to promote blood circulation and relieve pain. **Table 2** shows the herbs commonly used in combination, as determined by frequent itemset mining analysis. The frequent item sets were showed in **Supplementary Table 2**.

**Table 2:** The major Chinese herbs pairs/prescriptions in endometriosis treatment.

No.	Key combinations
1	*Sparganium stoloniferum* (Sanleng)*,Curcuma phaeocaulis* (Ezhu)
2	*Angelica sinensis* (Danggui)*, Paeonia lactiflora* (Chishao)
3	*Angelica sinensis* (Danggui)*, Ligusticum sinense Oliv* (Chuanxiong)
4	*Angelica sinensis* (Danggui) *, Corydalis yanhusuo* (Yanhusuo)
5	*Paeonia lactiflora* (Chishao)*, Cinnamomum cassia* (Guizhi)
6	*Salvia miltiorrhiza Bunge* (Danshen), *Paeonia lactiflora* (Chishao)
7	*Curcuma phaeocaulis* (Ezhu), *Salvia miltiorrhiza Bunge* (Danshen)
8	*Angelica sinensis* (Danggui), *parganium stoloniferum* (Sanleng)
9	*Corydalis yanhusuo* (Yanhusuo), *Paeonia lactiflora* (Chishao)
10	*Cinnamomum cassia* (Guizhi)*, Paeonia suffruticosa* (Mudanpi)
11	*Ligusticum sinense Oliv*(Chuanxiong), *Paeonia lactiflora* (Chishao)
12	*Cyperus rotundus L*.(Xiangfu), *Angelica sinensis* (Danggui)
13	*Typha angustifolia L*.(Puhang)*, Trogopterus xanthipes* (Wulingzhi)
14	*Ligusticum sinense Oliv* (Chuanxiong), *Paeonia lactiflora* (Chishao), *Angelica sinensis* (Danggui)
15	*Corydalis yanhusuo* (Yanhusuo), *Angelica sinensis* (Danggui), *Paeonia lactiflora* (Chishao)
16	*Ligusticum sinense Oliv* (Chuanxiong)*, Commiphora myrrha* (Moyao), *Corydalis yanhusuo* (Yanhusuo)
17	*Ligusticum sinense Oliv* (Chuanxiong)*, Cyperus rotundus L*. (Xiangfu), *Angelica sinensis* (Danggui)
18	*Ligusticum sinense Oliv*(Chuanxiong)*, Corydalis yanhusuo* (Yanhusuo), *Paeonia lactiflora* (Chishao), *Angelica sinensis* (Danggui)
19	*Curcuma phaeocaulis* (Ezhu), *Paeonia lactiflora* (Chishao), *Cyperus rotundus L*. (Xiangfu)
20	*Cinnamomum cassia* (Guizhi), *Prunus persica* (Taoren), *Paeonia lactiflora* (Chishao), *Paeonia suffruticosa* (Mudanpi), *Poria cocos* (Fuling)

To uncover latent knowledge, unsupervised machine learning and RNNs were applied to generate the knowledge graph, by calculating the distance between knowledge element vectors, according to preset categories. We set various parameters, and obtained multiple knowledge graphs. We obtained a ratio of nine clusters/four clusters of knowledge maps according to the clinical medication rules of Chinese medicine. Different clusters were formed by setting parameters and applying the combination rules of TCM. These clusters may reflect the law of compatibility and combination of clinical prescriptions in TCM. **Figures 2E, F** demonstrate the knowledge graphs for endometriosis herbs. The classifications reflect the possible combinations of TCM prescription medications. Based on the knowledge graphs and association rule mining, synergistic herbal combinations could be derived; however, further analysis of the associated mechanism is required.

### GO terms and KEGG Pathway Analysis of Endometriosis-Associated Genes

**Figure 3:**
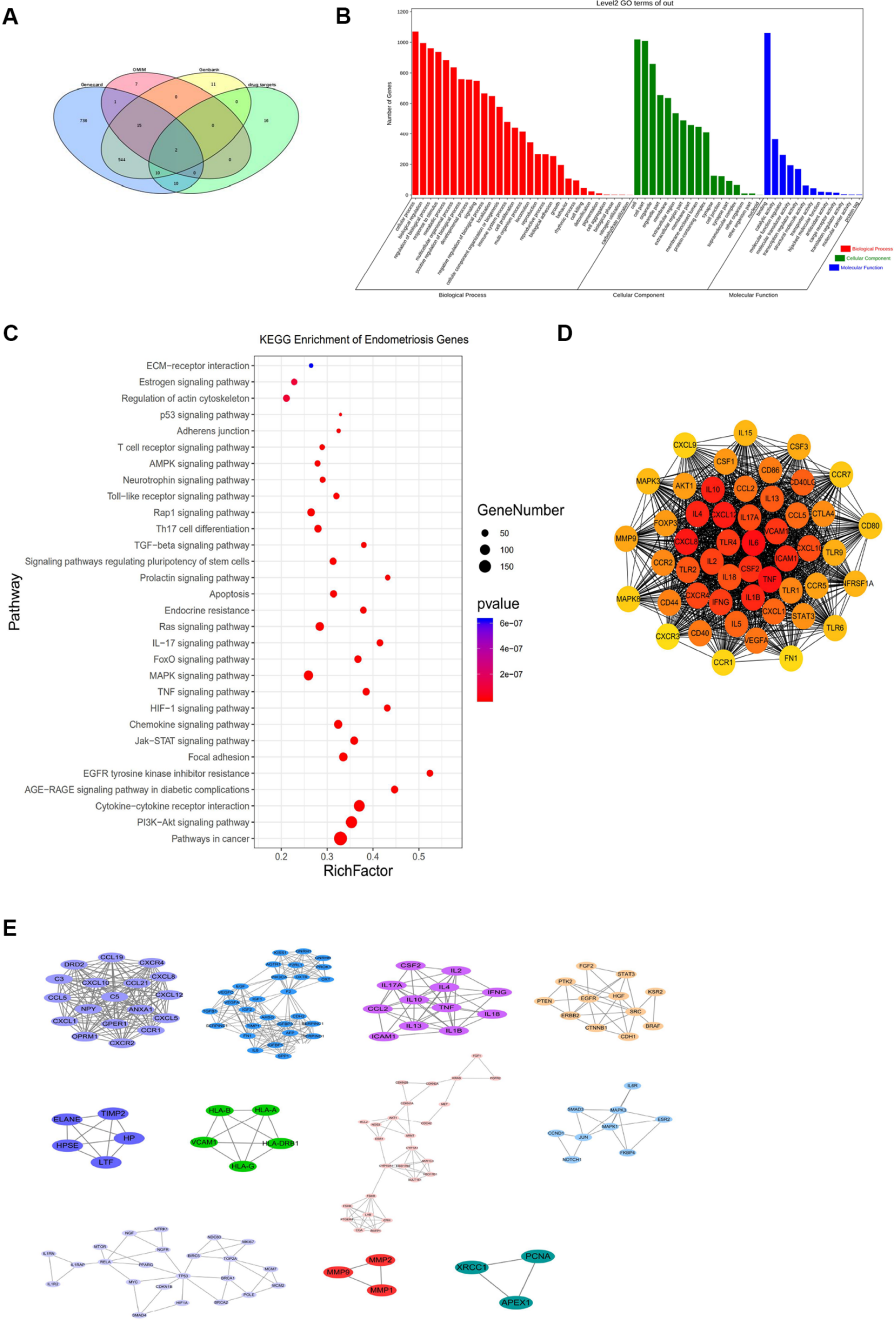
Kyoto Encyclopedia of Genes and Genomes (KEGG) and Gene Ontology (GO) enrichment analysis of endometriosis-associated genes. **(A)** Venn diagram showing analysis of endometriosis genes from different datasets. *MMP2, PTEN, LHB, BIRC5, EMX2, ENDO1, KRAS, NR5A1, AHRR, CCL11, MMP14, CCL5, ESR2, ARID1A*, and *MMP9* were the shared genes from the Genecard, Genbank, and OMIM datasets. **(B)** GO enrichment of endometriosis-associated genes. **(C)** KEGG pathway of endometriosis genes. Bubble chart for significantly enriched pathway terms from KEGG analysis. The size of each bubble is reflective of the relative ratio of the number of targets hitting each pathway over the total number of targets. *P* indicates the statistical significance of the *P*-values; greater numbers indicate greater significance. **(D)** Hub genes of endometriosis-associated genes, determined by cytoHubba in Cytoscape 3.7.2. **(E)** Cluster analysis of endometriosis-associated genes, determined by Molecular Complex Detection (MCODE) in Cytoscape 3.7.2.

We collected a total of 1,289 endometriosis-related targets from Genecard, Genbank, as well as the OMIM records of genomic databases of human diseases. The Venn diagram in **Figure 3A** reflects common genes from different databases. The genes significantly associated with endometriosis were tested for functional enrichment, including the relevant pathways and GO terms. The results of the GO analysis indicated that “inflammatory response,” “innate immune response,” and “chemokine production” were the most significant terms related to endometriosis in the BP category. Furthermore, the significant MF terms related to endometriosis were “cytokine activity,” “growth factor activity,” and “receptor binding.” The results of GO enrichement showed in **Figure 3B**.

The majority of the pathways were related to cytokine-cytokine receptor interactions, the PI3K-Akt signaling pathway, focal adhesion, the TNF signaling pathway, and HIF-1 signaling pathway, among others (**Figure 3C**). The KEGG pathways of endometriosis associated genes are showed in **Supplementary Table 3**. These signal pathways can affect the cell proliferation, migration and invasion of endometriosis.

We used the *cytoHubba* in Cytoscape to find the hub genes. The hub genes showed in **Figure 3D**. And we further used MCODE plugin densely connected regions to analyze the major modules in biological processes. The results showed that the genes associated with endometriosis could be divided into 11 clusters (**Figure 3E**). The PPI network of the major endometriosis genes is showed in **Supplementary Figure 1**. The genes in cluster 1, which is dominated by chemokines, are related to the chemokine signaling pathway and cytokine-cytokine receptor interactions. The genes in cluster 2 participate in receptor ligand activity, growth factor activity, HIF-1 signaling, PI3K-Akt signaling, and the Ras signaling pathway. The genes in cluster 3 are mainly interleukins, suggesting pathways related to inflammation. In addition, cluster 4 is related to kinase proteins. Cluster 7 is related to hormone regulation. Cluster 9 is closely related to tumor transcription regulation. Cluster 6 and cluster 10 are related to cell adhesion, intercellular adhesion molecules, vascular cell adhesion molecules, and matrix metalloproteinases. The pathogenesis of endometriosis is related to chemokine regulation, adhesion, invasion, angiogenesis, inflammation, immunity, and hormone regulation. These findings provide the genetic background of endometriosis for further network pharmacology research.

### Overall Ingredients and Targets of Common Chinese Herbal Treatments

Chinese herbal medicines each contain dozens or even hundreds of various ingredients. Thus, the compilation of an ingredient database is crucial. Some Chinese medicines are animal-derived compounds that were not included in the network pharmacological analysis. *Typha angustifolia L*. [Typhaceae] (Puhang) and *Faeces Togopteri* (Wulingzhi) represented one herbal pair (HP). The source of *F. Togopteri* is *Trogopterus xanthipes* feces; thus, we did not analysis this HP. The single herbs, HPs, and prescriptions were evaluated, to determine the characteristics of Chinese medicine more comprehensively. In order to further corroborate our predictions, a literature review was conducted to determine whether the compounds were already experimentally validated for any associated therapeutic effects. We analyzed gene enrichment for the major Chinese herbs, as hub herb associated targets. Compounds and targets from of major Chinese herbs for endometriosis treatment are listed in **Supplementary Table 4**. The Chinese herbs associated targets are listed in **Supplementary Table 5**.

#### *Salvia miltiorrhiza* Bunge[Lamiaceae] (Dangshen)

More than 200 compounds from *Salvia miltiorrhiza Bunge* were found, including lipophilic diterpenoids, water-soluble phenolic acids, and other constituents. The active ingredients of *S. miltiorrhiza* include tanshinone I, tanshinone IIA, salvianolic acid, and dihydrotanshinone, among others (MEIm et al., 2019). A total of 196 targets were possibly related to *S. miltiorrhiza*, and 63 targets could be associated with endometriosis genes. Based on the KEGG pathway analysis, the main pathways were those associated with cancer, calcium signaling, VEGF signaling, T cell receptor signaling, progesterone-mediated oocyte maturation, apoptosis, and p53 signaling pathway, among others that were related to endometriosis (*P* < 0.05). Compounds such as ursolic acid, rosmarinic acid, ferulic acid, caffeic acid, tanshinone IIA, protocatechuic acid, and tetramethylpyrazine could evidently regulate pain-associated targets to reduce pain, based on the network pharmacological analysis.

#### *Angelica Sinensis (Oliv.)/Angelica Sinensis* Var. Wilsonii (H.Wolff) Z.H.Pan & M.F.Watson (Danggui)

*Angelica sinensis* var. *wilsonii* (H.Wolff) Z.H.Pan & M.F.Watson is predominantly known as a treatment for intractable gynecological disorders. Ultrahigh performance liquid chromatography-tandem mass spectrometry (UHPLC-MS/MS) showed that *A. sinensis contains* eight components, including ferulic acid, senkyunolide A, butylphthalide, ligustilide, butylidenephalide, senkyunolide I, senkyunolide H, and levistolide A (Gui and Zheng, 2019). The volatile oil of *A. sinensis*, has evident antiinflammatory activities (Zhong et al., 2016). A total of 86 targets were possibly related to *A. Sinensis*, and 27 targets were associated with endometriosis genes. The KEGG enrichment showed associated signaling pathways in neuroactive ligand-receptor interactions, calcium signaling, TNF signaling, cGMP-PKG signaling, and the estrogen signaling pathway.

#### *Corydalis yanhusuo* (Y.H.Chou & Chun C.Hsu) (Yanhusuo)

*Corydalis yanhusuo* (Y.H.Chou & Chun C.Hsu) could effectively attenuate acute inflammatory and neuropathic pain. The main alkaloid contents and composition of *C. yanhusuo* includes protopine, α-allocryptopine, tetrahydrocolumbamine, coptisine, palmatine, berberine, dehydrocorydaline D, L-tetrahydropalmatine, tetrahydroberberine, corydaline, and tetrahydrocoptisine, as determined by the high performance liquid chromatography-diode array detector (HPLC-DAD) method. A total of 165 targets were possibly related to *C. yanhusuo*, and 47 targets were associated with endometriosis genes. The KEGG enrichment analysis revealed pathways associated with cancer, calcium signaling, neuroactive ligand-receptor interactions, VEGF signaling, apoptosis, T cell receptor signaling, and B cell receptor signaling pathway, among others.

#### *Ligusticum chuanxiong* S.H.Qiu, Y.Q.Zeng, K.Y.Pan, Y.C.Tang & J.M.Xu/*Conioselinum anthriscoides* ‘Chuanxiong’ (Chuanxiong)

*Conioselinum anthriscoides* ‘Chuanxiong’ is used to regulate menstruation and relieve pain in multiple diseases. A total of 174 ingredients of *L. chuanxiong* and 22 compounds demonstrated favorable bioavailability (Chen Z. et al., 2018). A total of 95 targets may be related to *L. chuanxiong*, and 26 targets were associated with endometriosis genes. Based on the KEGG enrichment analysis, we obtained the following associated pathways: neuroactive ligand-receptor interaction, calcium signaling, pathways in cancer, and VEGF signaling pathway. We inferred that fumarine, isocorypalmine, and fagarine I could regulate the 5-hydroxytryptamine receptor to reduce pain in endometriosis.

#### *Astragalus mongholicus* Bunge (Huangqi)

*Astragalus mongholicus* Bunge is one of the most popular traditional medicinal herbs with several pharmacological activities, including hematopoietic, antiinflammatory, and immunological. The main components of *A. mongholicus* were hederagenin, kumatakenin, isorhamnetin, 3,9-di-O-methylnissolin, calycosin, 7-O-methylisomucronulatol, formononetin, quercetin, and betulinic acid (Fu et al., 2014). A total of 263 targets were possibly related to *A. membranaceus*, and 68 targets were associated with endometriosis genes. The KEGG enrichment analysis showed the following pathways: cancer, neuroactive ligand-receptor interactions, calcium signaling, endometrial cancer, p53 signaling, T cell receptor signaling, metabolism of xenobiotics by cytochrome P450, toll-like receptor signaling, and VEGF signaling pathway, among others.

#### *Cyperus rotundus L*. (Xiangfu)

*Cyperus rotundus L*., a widely distributed perennial sedge has a relatively higher concentration of active ingredients in the form of essential oils, phenolic acids, ascorbic acids, and flavonoids in the tuber and rhizomes. *C. rotundus* is widely used in many disorders such as inflammation, diabetes, diarrhea, tumors, among others (Pirzada et al., 2015). A total of 246 targets were possibly related to *C. rotundus*, and 74 targets were associated with endometriosis genes. The KEGG enrichment analysis showed the following pathways: PI3K-Akt signaling pathway, neuroactive ligand-receptor interaction, TNF signaling pathway, MAPK signaling pathway, and HIF-1 signaling pathway, among others.

#### *Commiphora myrrha* (T.Nees) Engl. (Moyao)

*Commiphora myrrha* (T.Nees) Engl., the Commiphora species, known as “myrrh,” are characterized by resinous exudates from the bark of plants. They are used in the treatment of trauma, arthritis, and fractures, and exert antiproliferative, antioxidant, antiinflammatory, and antibacterial effects (Shen et al., 2012). The compounds of C. myrrha include terpenoids, steroids, flavonoids, sugars, and lignans, among others. Furanosesquiterpenes, such as furanoelemanes, furanoeudesmanes, and furanogermacranes, are compounds with analgesic effects. A total of 213 targets were possibly related to *C. myrrha*, and 104 targets were associated with endometriosis genes. The KEGG enrichment analysis showed the following pathways: TNF signaling, HIF-1 signaling, toll-like receptor signaling, and PI3K-Akt signaling, among others.

#### *Carthamus tinctorius* L. (Honghua)

*Carthamus tinctorius L*. can invigorate blood circulation and has been recently shown to have antioxidant, analgesic, antiinflammatory, and antidiabetic properties. Carthamidin, carthamone, carvacrol, and isocarthamidin luteolin are the main constituents of *C. tinctorius L. (*Asgarpanah and Kazemivash, 2013). Furthermore, hydroxyethylcarthamin, hydroxysafflor yellow A (HSYA), safflor yellow B (SYB), safflomin A, safflomin B, safflomin C, isosafflomin C, safflor yellow A (SYA), and precarthamin, among others, have also been reported as constituents (Yue et al., 2013). A total of 74 targets were possibly related to *C. tinctorius*, and 37 targets were associated with endometriosis genes. The KEGG enrichment analysis showed the following significant pathways: cancer, FoxO signaling, endometrial cancer, and drug metabolism - cytochrome P450, among others.

### HPs for Endometriosis Treatment

#### *Curcuma phaeocaulis* (Ezhu) and *Sparganium Stoloniferum* (Buch.-Ham. ex Graebn.) Buch.-Ham. ex Juz. (Sanleng)

*Curcuma phaeocaulis* Valeton is the most widely used species in HPs for endometriosis in Chinese medicine, and its essential oils are widely applied in the treatment of tumors in China. In *C. Rhizoma*, curcumol, bisdemethoxycurcumin, (4S,5S)-germacrone-1,4-diepoxide, aromadendrene, hederagenin, epoxycaryophyllene, and calarene showed relatively higher levels of OB and DL (Zhou Y. et al., 2016). *S. stoloniferum* is used for its hematopoietic functions, antiinflammatory activity, and immunological properties. Notable constituents include trans-gondoic acid, hederagenin, beta-sitosterol, formononetin, stigmasterol, and epibetulinic acid. A total of 130 targets were possibly related to *C. phaeocaulis* and *S.stoloniferum*. The GO enrichment analysis showed that *C. phaeocaulis* and *S.stoloniferum* could regulate apoptosis, cell death, and cell proliferation. The KEGG analysis showed significant pathways in cancer, neuroactive ligand-receptor interaction, calcium signaling, the cGMP-PKG signaling pathway, PI3K-Akt signaling pathway, apoptosis, and serotonergic synapse, among others. A total of 38 targets could be associated with endometriosis genes. Topological analysis showed the major targets included: *TP53, SRC, TNF, VEGFA, PIK3CA, IL8*, and *EGFR*, among others, which are also the core genes that cause endometriosis.

#### Core Prescriptions-*GFW*

GFW was the core prescription used in data mining for endometriosis treatment, as well as the classical prescription in Chinese medicine. A total of 565 herbal compounds could be identified from databases. There were 230 targets in *Cinnamomum cassia* (L.) J.Presl [Lauraceae](Guizhi); 78 targets in *Paeonia suffruticosa* Andrews [Paeoniaceae](Mudanpi); 135 targets in *Paeonia lactiflora* Pall.(Chishao); 67 targets in *Prunus persica* (L.) Batsch(Taoren); and 55 targets in *Poria cocos(Schw.)Wolf*(Fuling). In *Cinnamomum cassia*, taxifolin; beta-sitosterol; sitosterol; catechin; ent-epicatechin; and peroxyergosterol all showed effective OB and DL levels. Protocatechuic acid, coumarin, cinnamyl alcohol, 2-methoxy cinnamic acid, cinnamic acid, and cinnamaldehyde are considered the core components of *C. cassia*, and the core herbs in such prescriptions. The target proteins in *C. cassia* were focused on neurological disease, inflammatory disease, cancer, cellular growth and proliferation, cell signaling, and molecular transport. *P. lactiflora* is used for pain and blood stasis, and has hematopoietic functions, antiinflammatory activity, and immunological properties. And a total of 94 of its targets were associated with endometriosis treatment. Furthermore, 70 targets in *P. suffruticosa*, 16 targets in *P. cocos* and 10 targets in *P. persica* could be associated with endometriosis genes.

In the present study, we obtained 521 targets (**Supplementary Table 5**), which may be regulated by the above mentioned Chinese herbs derived from databases. These targets were involved in kinase pathways, angiogenesis, inflammation, immunity, and other modules. The main pathways included those of neuroactive ligand-receptor interactions, toll-like receptor signaling, metabolism of xenobiotics by cytochrome P450, VEGF signaling, apoptosis, drug metabolism, endometrial cancer, and the calcium signaling pathway, among others.

**Figure 4:**
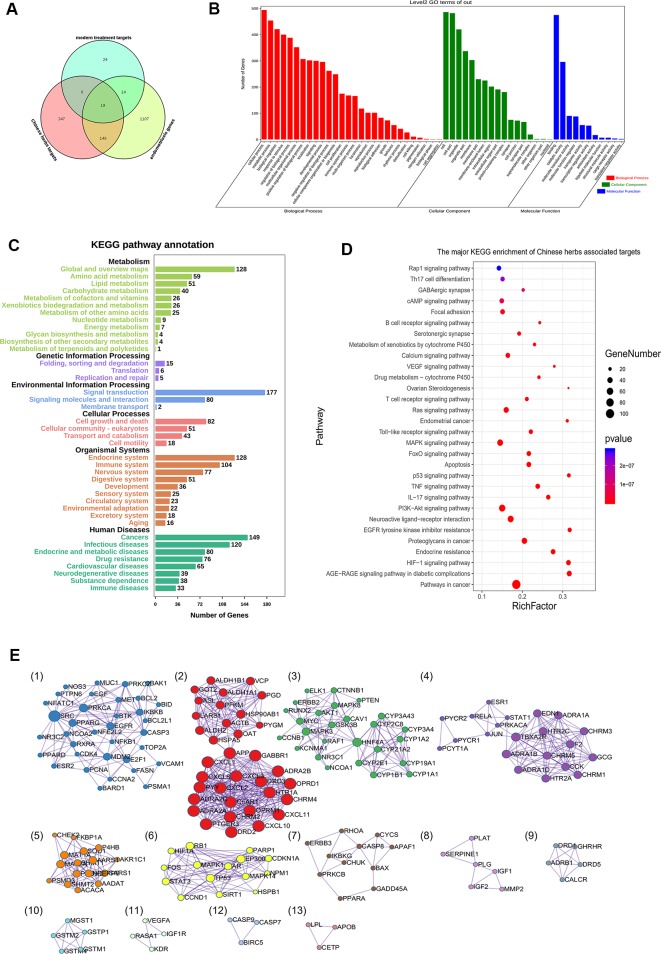
Multiregulation effect of all targets in selected Chinese medicines. These traditional Chinese medicines are mainly therapeutic medicines and official prescription medicines, which could regulate multiple pathways involving nerves, inflammation, and immunity. These actions reflect the multitarget regulatory mechanisms of Traditional Chinese medicine. **(A)** Venn diagram showing the common targets between Chinese herbs and conventional treatment drugs. **(B)** Gene Ontology (GO) enrichment analysis of Chinese herbs and associated targets. **(C)** Kyoto Encyclopedia of Genes and Genomes (KEGG) pathways in annotation. **(D)** KEGG pathway enrichment of the targets of Chinese herbs. **(E)** Module analysis of Chinese herbs, determined by Molecular Complex Detection (MCODE) of the Cytoscape software. These targets could be divided into 13 clusters. The MCODE_2 (pathways in cancer and response to reactive oxygen species); MCODE_3 (cytochrome P450 - arranged by substrate type); MCODE_6 (cellular responses to stress), MCODE_7 and MCODE_12 (apoptosis); MCODE_9 (adenylate cyclase-activating G protein-coupled receptor signaling pathway, cAMP-mediated signaling pathway); and MCODE_11 (VEGFR2 mediated cell proliferation, Ras signaling pathway) were all related to endometriosis pathogenesis.

The venn diagram of the common targets between Chinese herbs and conventional treatment drugs is showed in **Figure 4A**. The results of GO enrichment are presented in **Figure 4B**. The KEGG pathway annotation and KEGG pathways enrichment are presented in **Figures 4C, D**. The major KEGG pathways of major Chinese herbs, herbs pairs and prescription are showed in **Supplementary Table 6**. Furthermore, following cytoHubba analysis, we found that *VEGFA, MAPK3, JUN, AKT1, TP53, IL6, ALB, INS, MAPK1, MMP9, PTGS2*, among others could be core genes in the regulation of the screened herbs. Using MCODE analysis, we determined the modules of various herbs (**Figure 4E**). The results of the modules were related to the pathogenesis of endometriosis.

### Overlap of Chinese Herbs and Endometriosis Disease Modules

**Figure 5:**
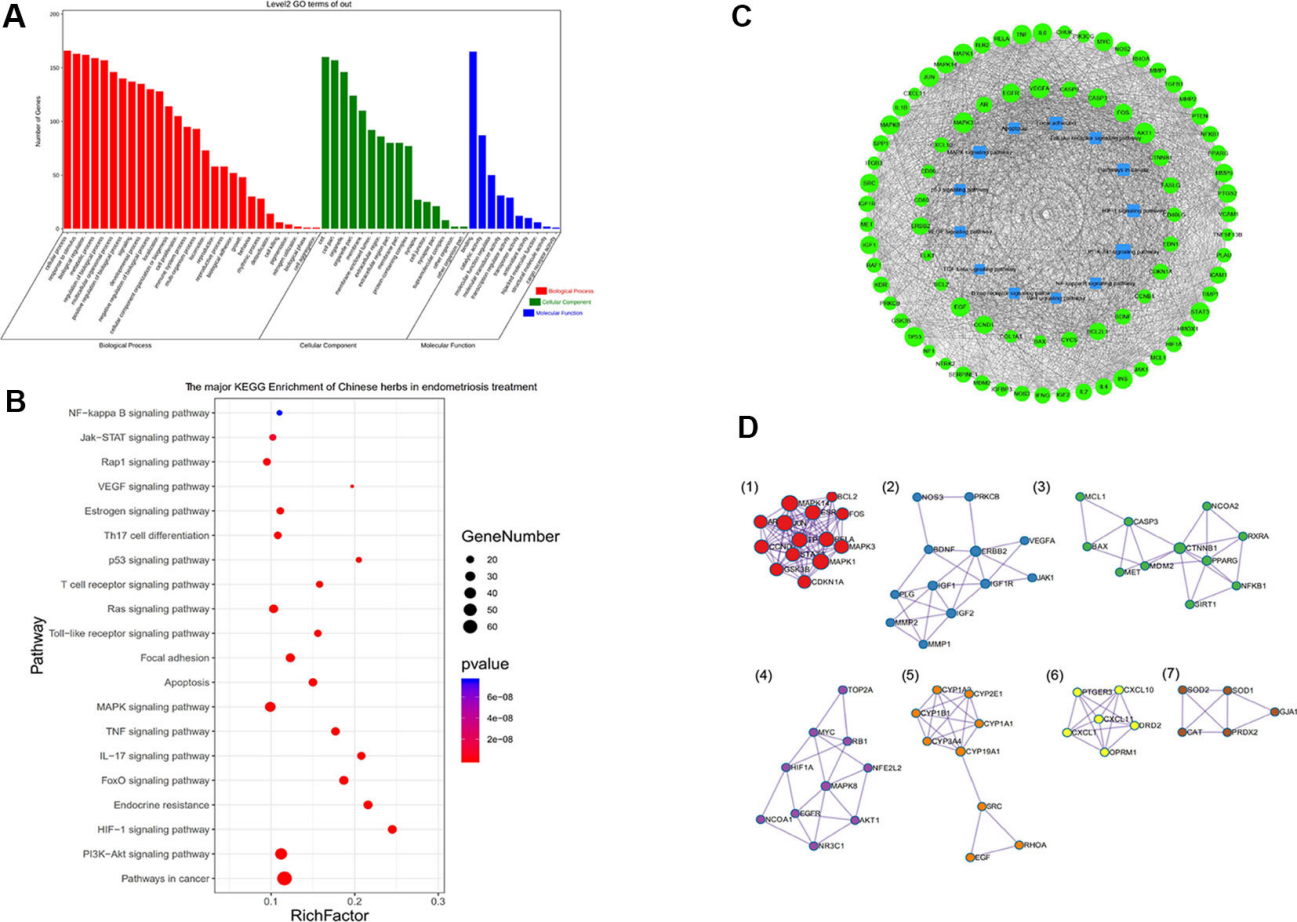
Kyoto Encyclopedia of Genes and Genomes (KEGG) and Gene Ontology (GO) enrichment analyses of the targets of Chinese medicine in endometriosis treatment. **(A)** GO analysis of the targets of endometriosis-associated genes that coincided with those of Chinese herbs. **(B)** KEGG enrichment of endometriosis-associated genes that coincided with those of Chinese herbs. **(C)** Pathways and targets network of Chinese medicine in endometriosis treatment. **(D)** Module analysis of the targets of Chinese medicine in endometriosis treatment, determined by Molecular Complex Detection (MCODE) of the Cytoscape software.

We further analyzed the targets of endometriosis-associated genes that coincided with those of Chinese herbs. A total of 170 targets of Chinese herbal medicines coincided with those of genes associated with endometriosis. The targets of endometriosis-associated genes that coincided with those of Chinese herbs are showed in **Supplementary Table 7**. Chinese herbal treatment for endometriosis could regulate the biological processes of “regulation of apoptosis,” “regulation of cell death,” and “response to hormone stimulus,” among others. **Figure 5A** shows the GO enrichment of herb-associated targets. The GO enrichment of endometriosis-associated genes that coincided with Chinese herbs are showed in **Supplementary Table 8**.

We also identified associated pathways, such as those of cancer, PI3K-Akt signaling, MAPK signaling, FoxO signaling, focal adhesion, HIF-1 signaling, Ras signaling, TNF signaling, estrogen signaling, toll-like receptor signaling, and VEGF signaling pathway, among others. To determine the functions of Chinese herbs, significant pathway terms in KEGG analysis were mapped onto a bubble graph (**Figure 5B**). Larger and higher bubbles in the Figure represent the highly, significantly enriched pathway terms. The network of major pathways and targets is presented in **Figure 5C**. **Table 3** shows the major KEGG pathways of Chinese herbs in endometriosis treatment.

**Table 3:** The Kyoto Encyclopedia of Genes and Genomes (KEGG) pathways of endometriosis-associated genes that coincided with Chinese herbs.

KEGG class	Pathway	out	All	Pvalue
Cancers	Pathways in cancer	64	550	6.88E-36
Endocrine and metabolic diseases	AGE-RAGE signaling pathway in diabetic complications	28	114	2.14E-24
Signal transduction	PI3K-Akt signaling pathway	42	374	3.13E-22
Signal transduction	HIF-1 signaling pathway	25	102	8.08E-22
Drug resistance	EGFR tyrosine kinase inhibitor resistance	23	82	1.32E-21
Drug resistance	Endocrine resistance	25	116	2.48E-20
Signal transduction	FoxO signaling pathway	26	139	1.84E-19
Cancers	MicroRNAs in cancer	27	168	2.15E-18
Immune system	IL-17 signaling pathway	22	106	1.30E-17
Signal transduction	TNF signaling pathway	23	130	9.72E-17
Signal transduction	MAPK signaling pathway	33	332	7.65E-16
Cell growth and death	Apoptosis	23	153	4.02E-15
Cellular community - eukaryotes	Focal adhesion	26	212	8.70E-15
Endocrine system	Prolactin signaling pathway	16	74	2.22E-13
Immune system	Toll-like receptor signaling pathway	19	122	6.04E-13
Signal transduction	Ras signaling pathway	25	243	1.73E-12
Immune system	T cell receptor signaling pathway	18	114	2.00E-12
Cell growth and death	p53 signaling pathway	15	73	2.86E-12
Signal transduction	ErbB signaling pathway	15	87	4.15E-11
Nervous system	Neurotrophin signaling pathway	17	124	8.75E-11
Immune system	Th17 cell differentiation	20	186	1.58E-10
Cell growth and death	Apoptosis - multiple species	10	33	2.19E-10
Endocrine system	Estrogen signaling pathway	19	171	2.66E-10
Signal transduction	VEGF signaling pathway	12	61	8.10E-10
Signal transduction	Rap1 signaling pathway	20	211	1.51E-09
Signal transduction	Jak-STAT signaling pathway	17	167	9.50E-09
Signal transduction	NF-kappa B signaling pathway	14	127	7.55E-08
Immune system	B cell receptor signaling pathway	11	74	9.07E-08
Endocrine system	GnRH signaling pathway	11	96	1.35E-06
Signal transduction	TGF-beta signaling pathway	7	92	0.001496753

The MCODE plugin was used to analyze the modules. Cluster 1 was related to endocrine resistance, prolactin signaling pathway, and pathways in cancer. Cluster 2 was related to signaling by receptor tyrosine kinases and EGFR tyrosine kinase inhibitor resistance. Cluster 3 was related to responses to steroid hormones. Cluster 4 was related to the Pathway Interaction Database ceramide pathway. Cluster 5 was related to steroid hormone biosynthesis, cytochrome P450-arranged by substrate type, and estrogen metabolic process. Cluster 6 was related to the adenylate cyclase-activating G protein-coupled receptor signaling pathway. Cluster 7 was related to cellular detoxification in GO enrichment (**Figure 5D**). Module analysis also facilitated the discovery of potential sub-modules of TCM in the regulation of endometriosis.

### Target-Pathway Network

**Figure 6:**
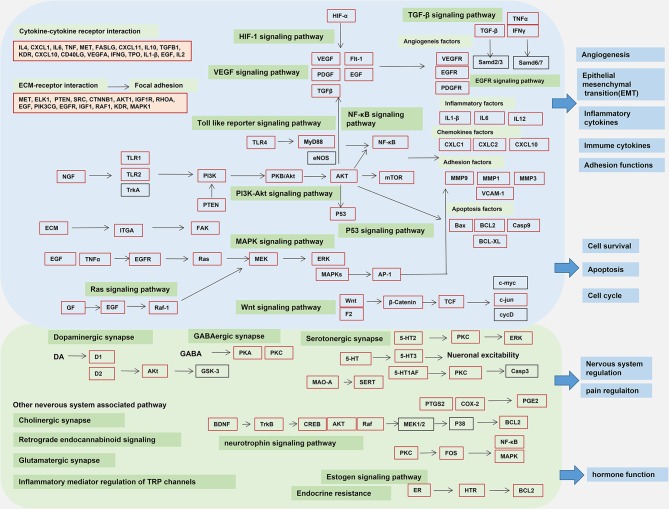
Pathway of multiregulatory effects in Chinese medicine. Target network showing multiregulation in Chinese medicine. The reconstructed Kyoto Encyclopedia of Genes and Genomes (KEGG) map reflects the multipath, multitarget regulatory pathway of Chinese medicine. Target names are shown in rectangles. The pathway network with a blue background is a pathway module that is directly related to endometriosis in Chinese medicine. The green background indicates the network of Chinese medicine that regulates endometriosis-associated pain and estrogen regulatory pathways. The red border indicates the direct target for Chinese medicine treatment in endometriosis. The black border indicates the indirect target. The arrows in the figure indicate the upstream and downstream genes in pathway actions of the targets. Interactions between the pathways were noted. Each pathway target synergistically regulates endometriosis pain, inflammation and immunity, adhesion, invasion, and angiogenesis.

We constructed a KEGG map of Chinese medicine associated targets to reflect the regulation of Chinese medicine on endometriosis genes. TCM affects the development of endometriosis through multiple interactions such as inflammation, immunity, angiogenesis, and kinase pathways. Multiple pathways were integrated and overlapped, based on cross-talk targets.The common targets showed the largest overlap with the cancer signaling pathway. The multiregulation map of the KEGG pathway is shown in **Figure 6**. By mapping the targets to related pathways, we found that endometriosis treatment is mostly related to four function modules of the mechanism pathways. The cross-talk pathways network of Chinese herbs in endometriosis treatment is showed in **Supplementary Figure 2**. Therefore, we gained a deeper understanding of the mechanisms of these pathways.

Pain relieving function is the major module in Chinese treatment, which is related to the nervous system in KEGG categories, such as neuroactive ligand-receptor interaction, serotonergic synapse, GABAergic synapse, cholinergic synapse, neurotrophin signaling pathway, dopaminergic synapse, calcium signaling pathway, glutamatergic synapse, and cAMP signaling pathway, among others.

The major targets, acetylcholinesterase (AChE), adrenoceptor beta, HTR2A prostaglandin endoperoxide synthase (PTGS), brain-derived neurotrophic factor (BDNF), serotonin receptors (HTR2A, HTR3A, HTR1A, and HTR2C) are all related to the regulation of neural receptors. The regulatory mechanism of these targets can directly affect various neurotransmitter receptors and the synthesis of neurotransmitters to modulate pain. Chinese medicine could also indirectly affect pain through downstream immune inflammatory factors (IL6, IL10, and TNF), and immuno-inflammatory pathways, such as cytokine-cytokine receptor interactions, the TNF signaling, VEGF signaling, HIF-1 signaling, toll-like receptor signaling, and PI3K-Akt signaling pathway. Estrogen could also regulated of the central serotoninergic system in pain.

The second module was associated with cell growth and death, invasion, adhesion, and angiogenesis-related signaling pathways in endometriosis. Chinese medicine could regulate invasion and adhesion-related signaling pathways, induce apoptosis, and inhibit cell proliferation, through processes such as apoptosis, P53 signaling, PI3K-Akt signaling, Wnt signaling, ECM-receptor interaction, and focal adhesion, among others. The angiogenesis and the tumor-related functions also showed considerable overlap, which included HIF signaling and the VEGF signaling pathway.

The third module was associated with inflammation and the immune system. Related processes included cytokine-cytokine receptor interaction, toll-like receptor signaling, T cell receptor signaling, B cell receptor signaling, NF-κB signaling, and IL-17 signaling pathway, which are also important in regulating inflammation and immune responses.

Moreover, kinase signaling pathways in endometriosis could affect the proliferation and differentiation of endometriosis cells, which could be potential targets for non-hormonal therapeutics. The associated module could be related to the canonical IKKβ/NFκB pathway, MAPK pathways, the PI3K/AKT/mTOR pathway, and the AMPK signaling pathway (McKinnon et al., 2016). These results suggest that multiple targets could affect various pathways to regulate the pathological processes of endometriosis.

### Target Comparison of Chinese Herbs/Natural Compounds and Conventional Drugs

In order to clarify the similarities and differences between the related targets of Chinese medicine and conventional drugs for endometriosis, we considered the targets of conventional therapeutic drugs for comparison. At present, the treatment options for endometriosis include NSAIDs and hormonal drugs (progestogens, dienogest, and GnRH agonists). A total of 85 protein targets of these drugs were identified from DrugBank and the TTD database of conventional drugs. The drug targets of endometriosis conventional treatment are listed in **Supplementary Table 9**. Conventional drugs for the treatment of endometriosis are mainly aimed at the nervous system and hormonal pathway. The core pathways of these targets included steroid hormone biosynthesis, retinol metabolism, drug metabolism-cytochrome P450, and pentose and glucuronate interconversions, among others.

A total of 17 Chinese medicine associated targets (*AR, BCHE, BCL2, CYP19A1, CYP1A2, CYP2C8, CYP3A4, ESR1, NR1I2, NR3C2, PGR, PLAT, PPARA, PPARG, PTGS1, PTGS2*, and *THBD*) coincided with the targets of conventional drugs for endometriosis treatment. The enrichment of overlapping genes was mainly focused on the regulation of hormones and pain relief. The mechanism of action of Chinese herbs was similar to that of current conventional treatments. Furthermore, Chinese medicine and its compounds provided more possibilities for multitarget therapy.

### Regulatory Effects of TCM on Related Targets of Endometriosis Pain

**Figure 7:**
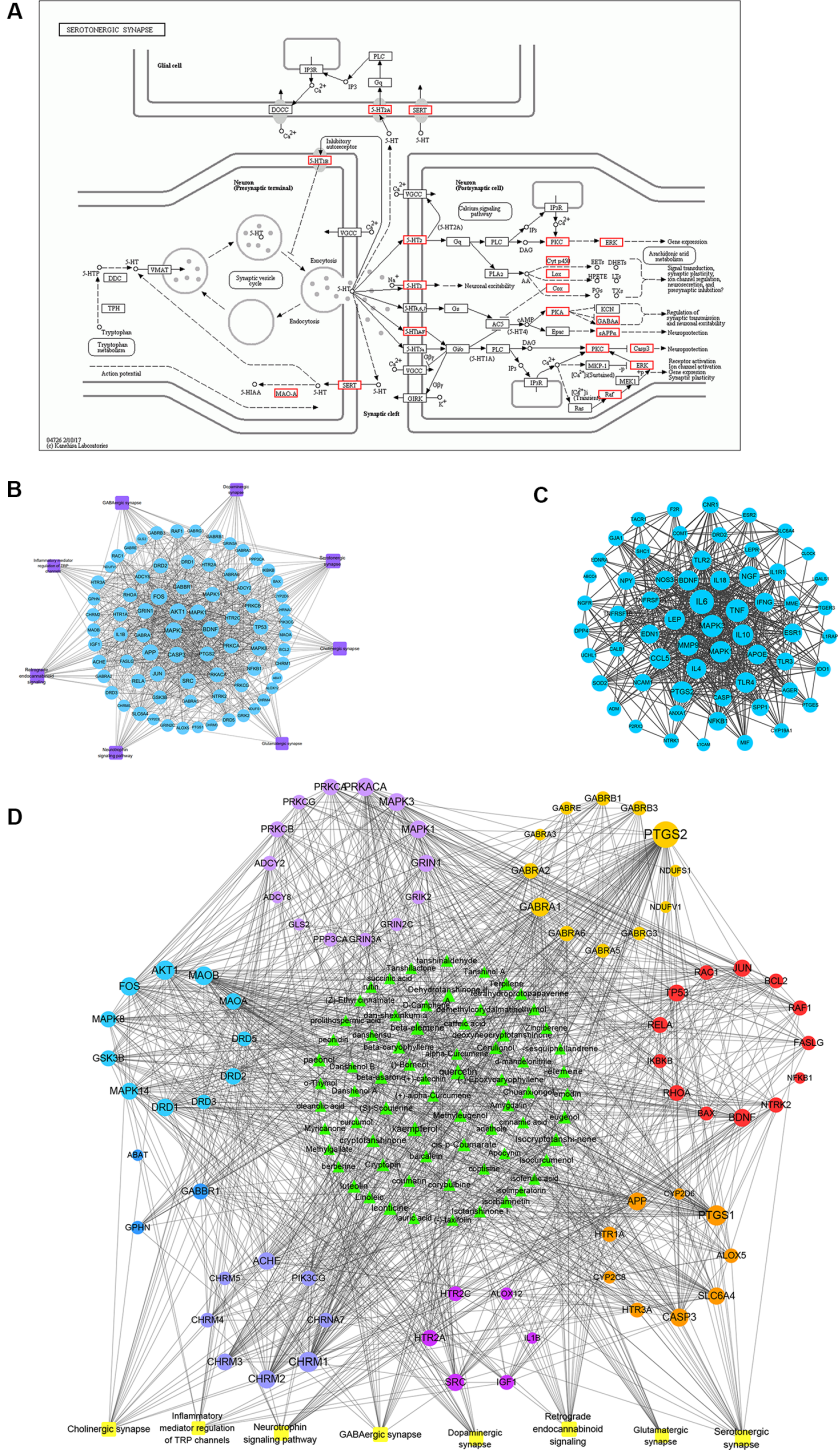
Kyoto Encyclopedia of Genes and Genomes (KEGG) pathway and sub-network analysis of traditional Chinese medicine for the endometriosis pain module. The pain module in endometriosis showed multiple neurotransmitters, nervous system pathways, and direct involvement in pain regulation. **(A)** KEGG mapper of the serotonergic synapse. **(B)** The network of targets network of pain associated pathways. Pathway of multineurotransmitter and inflammation, and immunity regulation network in endometriosis-associated pain. Purple nodes represent pathways, and blue nodes represent targets. **(C)** The protein-protein interaction (PPI) network of pain associated genes in Chinese medicine treatment. **(D)** Network of major compounds and targets in endometriosis-associated pain treatment. The middle green node represents pain treatment-related compounds, and the surrounding nodes are composed of different colors, which represent the target points enriched, according to the pathways. And the yellow node represents pathways.

Endometriosis-related pain has been the main focus of TCM research. Thus, we further analyzed the related pathways of pain, to provide a basis for the discovery of effective pain-relieving compounds. The analgesic effect of TCM is related to the regulation of neurotransmitters and related pain factors. The serotonergic synapse was the main network of neurotransmitter regulation, and its KEGG map is presented in **Figure 7A**. **Figure 7B** shows the network of targets network of pain associated pathways.

We also selected the targets of Chinese medicines for the treatment of endometriosis from the pain database. This database is a comprehensive network of contextualized PPIs specifically associated with pain, that has been created through study of the pain interactome (Jamieson et al., 2014). The results showed that a total of 61 genes were involved in pain prediction, which were also related to endometriosis. These included *PTGES, PTGS2, PTGS1, BDNF, TNF, IL6, ESR1, IL10, MMP9*, and *MAPK*, among others (**Figure 7C**). These compounds, which have similar targets to those of NSAIDs and opioid analgesics, may be considered active compounds in Chinese medicine for the treatment of various pain-related diseases in the future. **Figure 7D** shows the network of the “nervous system pathways-targets-compounds”.

We found that Chinese medicines with analgesic effects include polysaccharides, saponins, alkaloids, flavonoids, terpenoids and others. Compounds such as ursolic acid, rosmarinic acid, ferulic acid, caffeic acid, tanshinone IIA, and oleanolic acid, in *S. miltiorrhiza* could regulate pain targets. Interestingly, we also found that many of the compounds that regulate pain originate from volatile oils/essential oils molecules (de Cassia et al., 2017). Terpene compounds are the main components of volatile oils. Moreover, the combination of multiple compounds may further play a synergistic effect in Chinese medicine. These volatile oil compounds have 109 targets. The volatile oil compounds associated targets were showed in **Table 4**. We found that the DL value of volatile oils was low. Thus, the pharmaceutical activity of these compounds still needs further experimental verification.

**Table 4:** Volatile oils/essential oils from core herbs to associated pain.

Molecule name	Pubchem CID	MW	OB(%)	DL	Major source	Potential Targets
p-cymene(cymol)	7463	134.24	27.2	0.02	*Conioselinum anthriscoides* ‘Chuanxiong', *Salvia miltiorrhiza* Bunge, *Angelica sinensis*	SLC6A2,NET,E
carvacrol	10364	150.24	43.28	0.03	*Angelica sinensis*, *Cinnamomum cassia*	CHRM1,ADRB1,ADRA2C,SLC6A2,ADRA1A,SLC6A3,ADRB2,ADRA1B,ADRA1D
eugenol	3314	164.22	56.24	0.04	*Paeonia lactiflora* Pall. *Cinnamomum cassia*	VR1,CACNA1G,TRPA1,TRPV1,UGT2B17,MAOA,ALOX5,CACNA1H,TMPRSS11D,FIP1L1
menthol	165675	156.3	59.33	0.03	*Cinnamomum cassia*	TRPM8,TRPA1,TRPV3,TRPV1,ATF3,HTR3A,OPRK1,VR1,TMPRSS11D,TRPM2
cinnamaldehyde	637511	132.17	31.99	0.02	*Cinnamomum cassia*	TRPA1,SLC2A4,PTGS2,NOS2,AKR1C2,MAPK8,MAPK14,NQO1,RELA,CASP8
beta-citronellol	101977	156.3	38.89	0.02	*Citrus medica L*.	ADH1C,PTGS2,NCOA6
(L)-alpha-terpineol	443162	154.28	48.8	0.03	*Angelica sinensis*, *Curcuma phaeocaulis*, *Cinnamomum cassia*	GABRA6,CHRM3,CHRM1 ,GABRA2,GABRA5,NET,CHRM2,ADRA1B,SLC6A2,GABRA1,CHRM3,CHRM1,SLC6A2,GABRA1,ADRA1A,IGHG1
vanillin	1183	152.16	52	0.03	*Ligusticum sinense Oliv* Angelica sinensis	TRPV3,MMP9,KCNK3,UGT1A10,UGT1A8,UGT1A3,UGT1A7,CA1,CA2
borneol	6552009	154.28	81.8	0.05	Cinnamomum camphora (L.) J.Presl *Paeonia lactiflora*, *Curcuma phaeocaulis* Valeton	CYP2C8,GABRA2,GABRA5,CHRM2,GABRA1,IGHG1,GABRA6,PTGS1,PTGS2,NET,MAOB,NCOA2
pulegone	442495	152.26	51.6	0.03	*Mentha canadensis L*.	GABRA2,GABRA1,CYP2C8,GABRA5,CHRM2,CHRM1,NET,GABRA6,CYP19A1,SPEN
limonene	440917	136.26	39.84	0.02	*Paeonia lactiflora* Pall. *Cinnamomum cassia* (L.) J.Presl	PTGS2,GABRA1,ADH1B,ADH1C,CYP2C8,NCOA2,CHRM2,GABRA2,CHRM1 ,GABRA5,IGHG1,GABRA6 CYP2C19,CYP2C9,PPARG NOS1,NOS3,NOS2,MTRR,POR,IPP
geraniol	637566	154.28	23.93	0.02	*Cinnamomum cassia* (L.) J.Presl	ADH1B,ADH1C,PGR,CCND1,MAPK3,CDK4,BAK1,HERC1,PRKCB,HMGCR,CYP2B6,SI,LCT
anethole	637563	148.22	32.49	0.02	*Cinnamomum cassia* (L.) J.Presl	CDH1,NFKBIA,MAPK3,MMP9,IKBKB,AKT1,MAPK1,MMP2,ADRA2C,NET,ADRA1A,SLC6A2,ADRB2,MAOB,MAOA,E,REN,PRSS3,CHRM1,NFKB3,JUN,IKBKG,IL2
peruviol	5356544	222.41	29.61	0.06	*Cinnamomum cassia* (L.) J.Presl	PTGS2,NET
carvone	439570	150.24	49.47	0.03	*Zingiber officinale Roscoe*(Shengjiang)	TP53,GABRA2,GABRA1,CYP2C8,GSTP1,GSR
(Z,Z)-farnesol	1549107	222.41	41.14	0.06	*Cinnamomum cassia* (L.) J.Presl	CASP3,FDFT1,MAOB,UGT1A3,UGT1A4,AKR1C3,AKR1B10,UGT1A1,UGT1A9,UGT2B4,PTGS1,PTGS2,RXRA,NET,MAOB
myrcene	31253	136.26	24.96	0.02	*Angelica sinensis*, *Ligusticum sinense Oliv*.	ADH1C,GABRA1
thymol	6989	150.24	41.47	0.03	*Cinnamomum cassia* (L.) J.Presl, *Conioselinum anthriscoides* ‘Chuanxiong’	TRPV3,UGT1A7,UGT1A1,UGT1A10,UGT1A9,CASP9,CASP8,UGT1A8,CASP3,WDFY2
β-caryophyllene	5281515	204.39	29.7	0.09	*Salvia miltiorrhiza* Bunge, *Curcuma phaeocaulis*	PTGS1,CHRM3,CHRM1,PTGS2,GABRA2,RXRA,CHRM2,ADRA1B,CHRNA2,GABRA1,NCOA2,GABRA6,NET,ADRA1A,SLC6A2,IL6
γ-terpinene	7461	136.26	33.02	0.02	*Paeonia lactiflora* Pall. *Conioselinum anthriscoides* *Salvia miltiorrhiza* Bunge, *Angelica sinensis* var. *wilsonii (H.Wolff) Z.H.Pan & M.F.Watson*	PTGS2,ACHE,GABRA1,DPP4,ADH1C,CYP2C8,ADH1A,ADH1B

### Related Small Molecule Drugs Screening by CMap Analysis

We used expression profile data of CMap analysis for drug discovery. By querying CMap, we screened compounds in likely drug targets showing a similar gene expression profile with the desired and chemopreventive conditions. The DEGs were downloaded from the GSE25628 expression file. This dataset contained three sets of sample data, and we chose the comparison of two sets of samples for analysis. The endometriosis eutopic groups were compared with the non-endometriosis eutopic group (healthy women group), to include 52 upregulated and 169 downregulated DEGs (*P* < 0.05 and |logFC| > 2). A total of 67 upregulated and four downregulated DEGs with ectopic endometrium and eutopic endometrium were detected among the patients with endometriosis.

As the results, among the drugs or natural compounds identified in Chinese medicine and plants, genistein, atractyloside, naringenin, canadine, ursolic acid, and lycorine showed a higher negative correlation with, and greater potential to effectively treat endometriosis. The natural compounds with highly significant correlations from CMap analysis results are listed in **Table 5**. Based on the results of CMap analysis, we identified potential natural compounds that could be beneficial in the treatment of endometriosis, and provided a basis for further drug discovery.

**Table 5:** The results of Connectivity Map (CMap) analysis.

Compounds name	Major source	mean	n	enrichment	*P-value*	specificity	Potential targets
**A: CMap analysis of DEGs of ectopic endometrium vs. eutopic endometrium in patients with endometriosis**.							
genistein	*Pueraria montana* var. *lobata (Willd.) Maesen & S.M.Almeida ex Sanjappa & Predeep*(Gegenhua)	−0.356	17	−0.527	0.00006	0	ESR1,PPARG,PTGS2,MAPK14,HSP90AB1,CDK13,CHEK1,PRKACA,PRSS1,PIK3CG,NFKB3,EGFR,AKT1,VEGFA,BCL2,FOS,CDKN3,BAX,CASP9,MMP9,MAPK3,MAPK1,TNF,JUN,NOS2,AHSA1,CASP3,TP53,LRP5,MDM2,RASGRF1,RAF1,HIF1A,IGF1R,STAT1,CRK2,ERBB2,AR,PPARG,ICAM1,IL-1beta,CCL13,SELE,VCAM1,FN1,CXCL8,SOD2,BIRC5,NOS3,TGFB1,SULT1E1,CCNB1,PTEN,HMGCR,BTK,CHEK2,PPARA,PCOLCE
vinblastine	*Catharanthus roseus* (L.) G.Don [Apocynaceae], (Changchunhua)	−0.815	3	−0.932	0.0005	0.0153	ABCB1,JUN,ABCB4,TUBA1A,TUBB4B,ABCC2,TUBB,ABCG2,TUBE1,TUBD1
atractyloside	*Xanthium strumarium subsp. strumarium* (Cangerzi)	−0.432	5	−0.661	0.01071	0.0758	ANO6,VDAC1,CFD,PDLIM5,SLC25A4,SLC25A5
naringenin	*Typha angustifolia L. [Typhaceae]* (Puhuang), *Curcuma aromatica Salisb./Curcuma longa L*.(Yujin)	−0.341	4	−0.668	0.02733	0.1129	PPARA,ABCB1,CYP1A2,APOB,CYP1B1,CCL2,HMOX1,RAPGEF1,BDNF,LDLR
**B: CMap analysis of DEGs of eutopic endometrium in patients vs. eutopic endometrium in healthy women**.							
canadine	*Coptis teeta Wall*. [Ranunculaceae] (Huanglian),*Corydalis yanhusuo* (Yanhusuo)	−0.633	4	−0.797	0.00334	0.0201	F3,DRD1
ursolic acid	*Salvia miltiorrhiza* Bung (Danshen)	−0.475	4	−0.735	0.00985	0.0146	PLAU,CTSB,VEGFA,BCL2,MMP2,TNF,JUN,IL6,TP53,MAPK8,PTGS2,FASN,MMP1,MMP3,MMP10,IL-1beta,SELE,PTGER3,PTGS1
lycorine	*Curculigo orchioides Gaertn*. [Hypoxidaceae] (Xianmao)	−0.404	5	−0.621	0.02055	0.2267	CHRM3,CHRM1,ADRB1,CHRM5,CHRM4,OPRD1,CHRM2,ADRA2B,ADRA1B,ADRB2,OPRM1
naringenin	*Typha angustifolia L. [Typhaceae]* (Puhuang), *Curcuma aromatica Salisb./Curcuma longa L*. (Yujin)	−0.34	4	−0.658	0.03133	0.121	PPARA,ABCB1,CYP1A2,APOB,CYP1B1,CCL2,HMOX1,RAPGEF1,BDNF,LDLR

### Discovery of Potential Compounds

**Figure 8:**
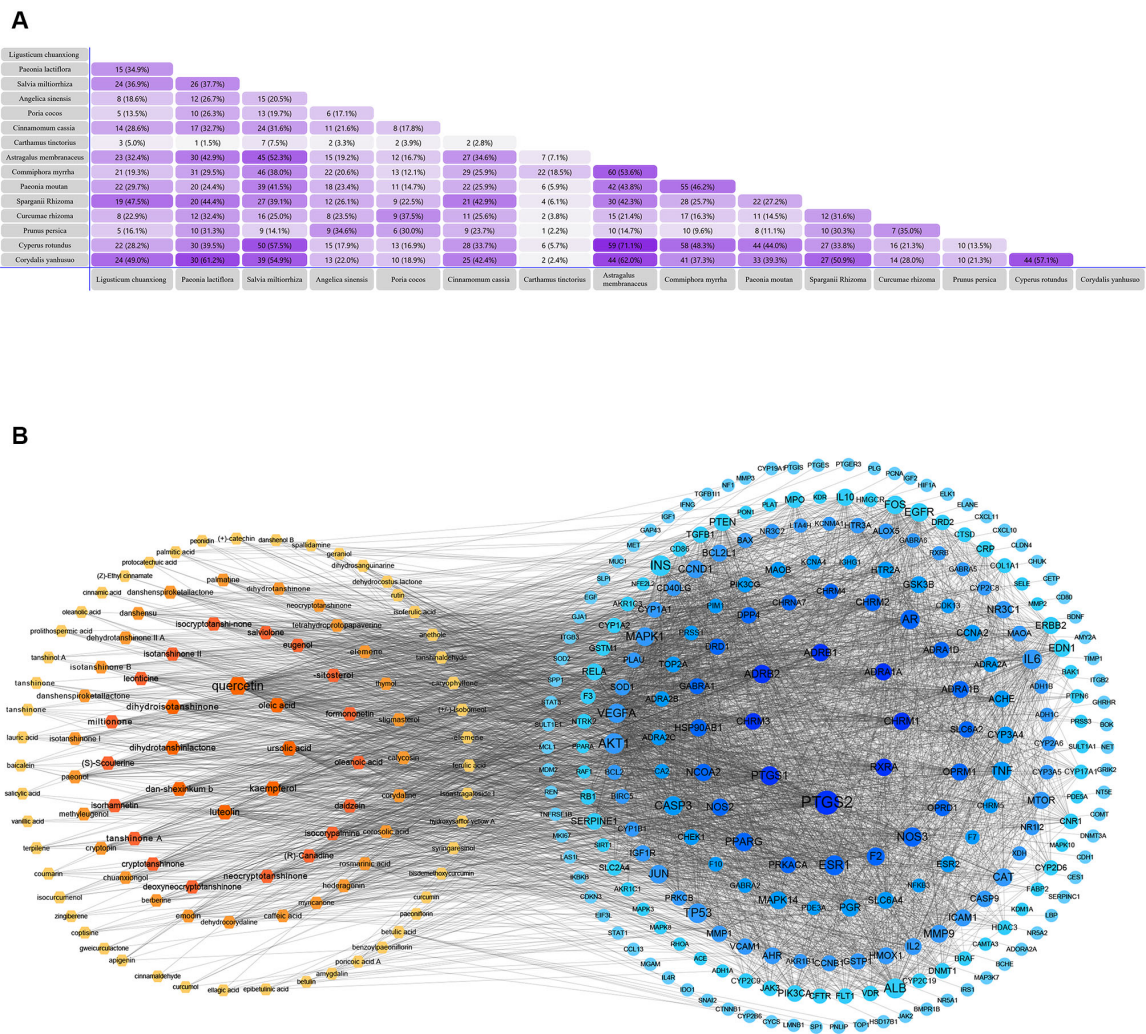
Discovery of potential compounds. **(A)** Venn diagram of major Chinese herbs associated targets. The venn diagram reflects the similarity of these Chinese medicines in regulating targets. **(B)** Network analysis to decipher the synergistic mechanisms of the compound-target network. We selected natural compounds that may have therapeutic effects for endometriosis and combined that information with molecular docking results. The compounds were derived from data mining of common Chinese herbs. Relationship between formula targets and drug targets. The network was constructed with compounds sharing the same targets between compounds. The data represents the ratio of formula targets and drug targets in biological processes. Blue nodes represent targets and orange nodes represent compounds. The deeper the color of a node, the more frequently it appears. Deeper colors may be the main targets and compounds. The bigger font size, the greater its degree based on undirected network analyisis. The major compounds were showed in **Table 6**.

Based on network pharmacology prediction, we identified some compounds that may have therapeutic effects, including polyphenolic compounds, sesquiterpenes, terpenoids, flavonoids, alkaloids, polysaccharides, and steroid glycosides. These compounds have better biological activity and are common in the literature regarding the treatment of endometriosis and diseases with similar target pathways. The Venn diagram of comomn targets of major Chinese herbs in endometriosis treatment was established by FunRich, an open access standalone functional enrichment and interaction network analysis tool (**Figure 8A**) (Pathan et al., 2015). The Venn analysis suggests that the targets of these Chinese herbs are similar, which means that these herbs could play synergistic role. Furthermore, compounds such as ursolic acid, rosmarinic acid, ferulic acid, tanshinone IIA, and oleic acid could regulate pain. Quercetin, salviolone, acetic acid, formononetin, luteolin, hederagenin, tanshinone II A, palmitic acid, cryptotanshinone, rutin, and curcumol could regulate the inflammatory or immunomodulatory response. These compounds could regulate core genes related to endometriosis and with more experimental verification, and could be potentially used in the treatment of endometriosis. The potential effective compounds are listed in **Table 6**, and the compounds and targets network is presented in **Figure 8B**. The potential compounds and targets are listed in **Supplementary Table 10**.

**Table 6:** The potential effective compounds and targets in endoemtriosis treatment.

Scientific Name(TCM names)	compounds name	PubChem CID	Molecular Formula	OB(%)	DL	Structure	Potential targets
*Sparganium stoloniferum* (Buch.-Ham. ex Graebn.) Buch.-Ham. ex Juz. (Sanleng)	betulin/trochol*	72326	C30H50O2	15.48	0.78	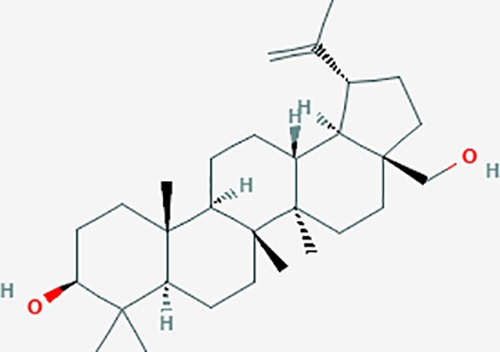	LAS1L,PGR,NOS2,ACE
	epibetulinic acid*	485711	C30H48O2	15.66	0.78	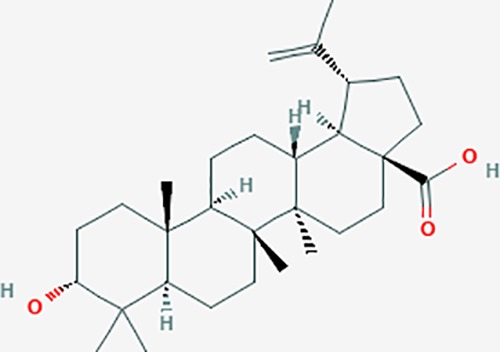	ADH1A,ADH1B,ADH1C,NCOA2,PGR
	hederagenin	73299	C30H48O4	36.91	0.75	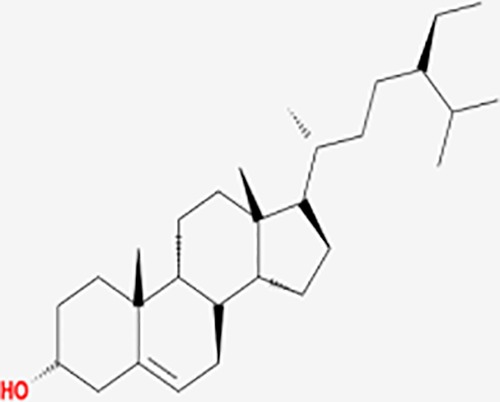	ADH1B,ADH1C,ADRA1B,ADRB1,CYP2C8,GABRA2,IGHG1,PGR,PTGS1,PTGS2,CHRM1,CHRM2,CHRM3,GABRA5,RXRA,PDE3A,ADRB1,GABRA1,NCOA2,GABRA6
*Salvia miltiorrhiza* Bunge (Danshen)	salvianolic acid A*	5281793	C26H22O10	2.96	0.70	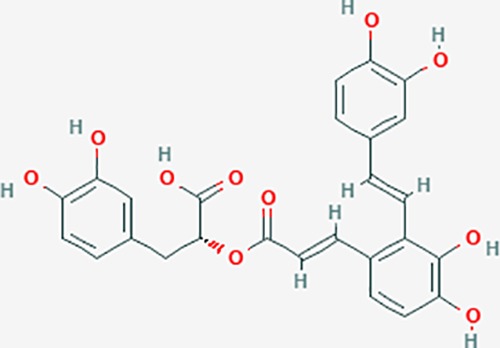	AKT1,BCL2,CDKN3,EIF3L,F10,PRSS1,CASP3, COL7A1,F7,PTPN6,CCND1
	dihydrotanshinone I	11425923	C18H14O3	45.04	0.36	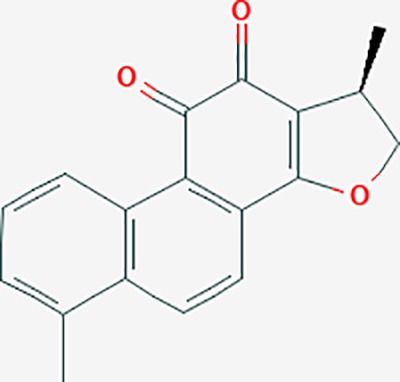	PIK3CG,ADRA1A,ADRA1B,ADRB1,ADRB2, CHRNA7,GABRA1,IGHG1,PRKACA,HTR3A, PTGS1,PTGS2,HSP90AB1,RXRA,NCOA2
	rosmarinic acid*	5281792	C18H16O8	1.38	0.35	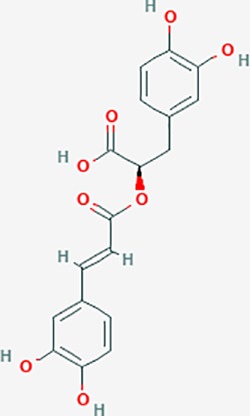	F2,ESR1,AR,PPARG,PTGS2,DPP4,PRSS1,NFKB3,IKBKB,CDKN3,EIF3L,MAPK1,CASP3,STAT1,CCL13,MGAM,IL2,IL4R,IDO1,IGHG1,
	oleic acid*	445639	C18H34O2	33.13	0.14	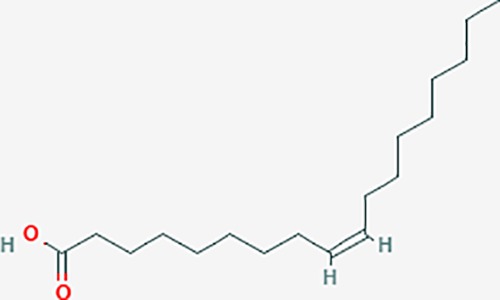	ADRA1D,ADRB1,ADRB2,EDN1,ERBB2,PLAU,SOD1,ADH1A,ADH1B,ADH1C,BDNF,CETP,CITED1,CRP,ENPEP,F10,FABP1,HMGCR,IGHG1,MPO,NCOA2,PLG,PON1,PPARG,PTGS1,PTGS2,RXRA,TEP1,UCP2,UCP3,GCG,SCD,INS,RXRB,DNPEP,RBP2,GAP43,SOAT1,CHRM1,PPARD,CAT,CCK,CHRM3,CYP2C8,LPL,NTRK2,PAM,PDE3A,PDX1,PPARA,PTPN6,PYY,SERPINE1,SLC2A1,KCNA4,KCNMA1,RHO,PGR
	tanshinlactone	5321617	C17H12O3	45.04	0.36	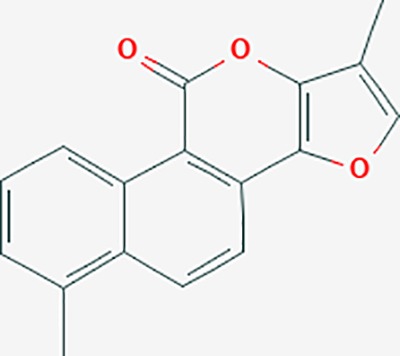	MMP9,ALB,MMP13
	tanshinol A	5321622	C18H12O4	21.31	0.41	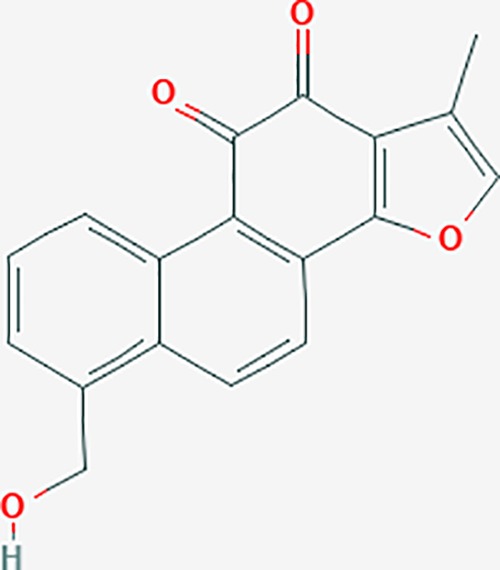	AR,F2,PIK3CG,DPP4,PTGS2,RXRA
	tanshinone IIA	164676	C19H18O3	49.89	0.40	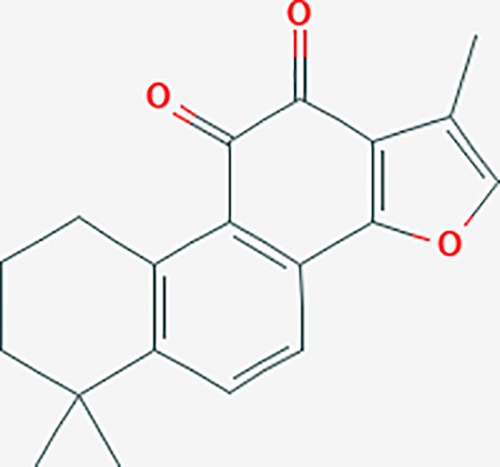	ACHE,ADRA1A,ADRB1,ADRB2,CASP3,CHRM1,F2,OPRM1,CHRM2,DPP4,RXRA,PTGS2, CHRM5,CHRNA7,OPRD1,CHRM3,CHRM4,DRD1,NFKB3,CYP1A1,EDN1,BCL2,FOS,TP53,CYP1A2,CYP3A4,ITGB3,JUN,MMP9
	tanshinone IIB	184102	C19H18O4	21.07	0.45	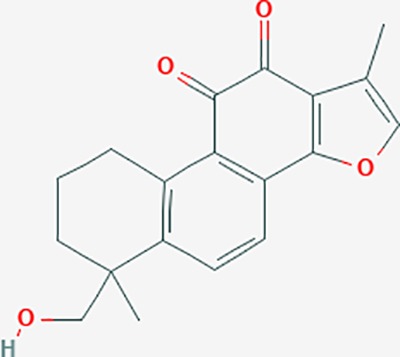	ACHE,ADRB1,ADRB2,AR,CCNA2,CHEK1,DPP4,OPRD1,OPRM1,GSK3B,PRSS1,ESR1,PTGS2CDK13,PIM1,CHRM1,F2,CHRNA7
	corosolic acid*	6918774	C30H48O4	15.16	0.74	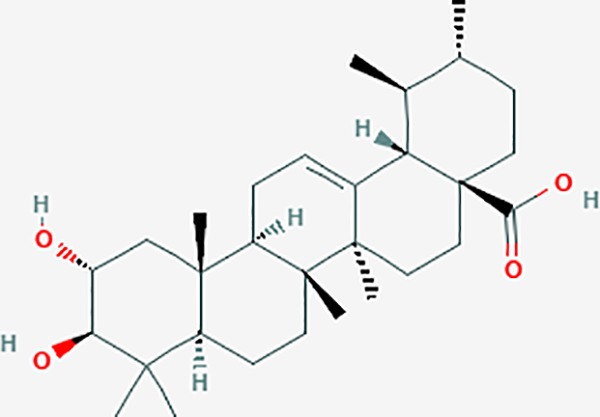	NTRK2,CYP2C9,JAK3,NR1I2,PIK3CA,MAPK1,NOS3,CFTR,FLT1,SNAI2,VDR,CYP3A4,ALB,CYP2C19,MTOR,GSK3B,DNMT1,CYP3A5,BRAF
	luteolin	5280445	C15H10O6	36.16	0.25	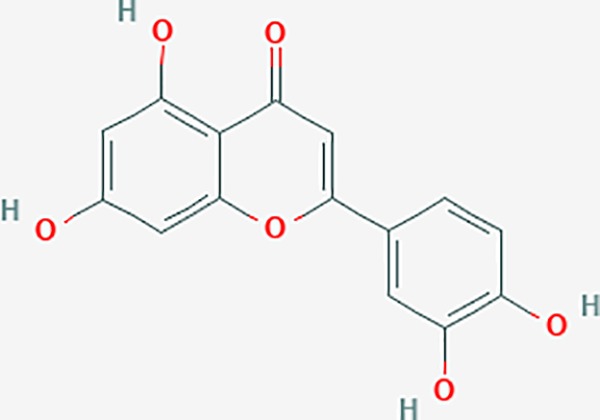	CASP3,CCND1,CDKN3,EGFR,IL6,PCNA,PTGES,TP53,TYR,MMP1,CD40LG,GSTP1,HMOX1,IL10,MMP9,PPARG,PRKACA,PRSS1,CASP7,ICAM1,IL2,MET,IL4R,CASP9,CCNB1,IKBKG,NUF2,PIK3CG,PTGS1,PTGS2,RB1,SLC2A4,TNF,TOP2A,XDH,ERBB2,JUN,MCL1,MDM2,NFKB3,HSP90AB1,INSR,NCOA2,VEGFA
	danshenol B	3083515	C22H26O4	57.95	0.56	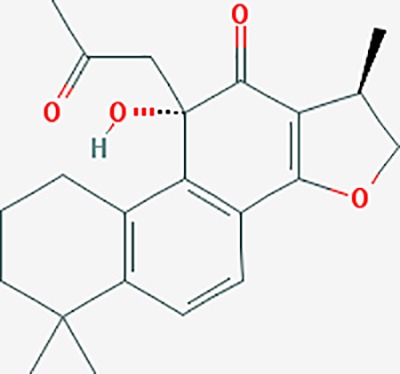	OPRM1,CA2,NR3C1,TOP2A,HSP90AB1,PTGS2,PGR
	cryptotanshinone	160254	C19H20O3	52.34	0.40	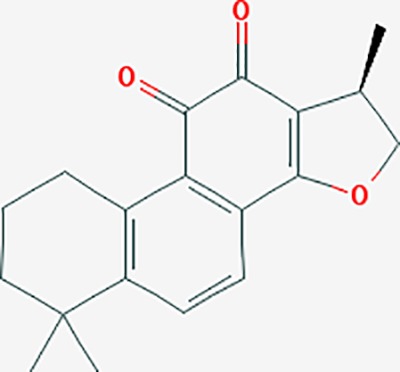	ADRA1A,ADRA1B,ADRA1D,ADRB1,ADRB2,APP,BCL2L1,BIRC5,CHRM1,CHRM3,CHRM4,CHRNA7
*Prunus persica* (L.) Batsch (Taoren)	amygdalin	34751	C20H27NO11	55.38	0.78	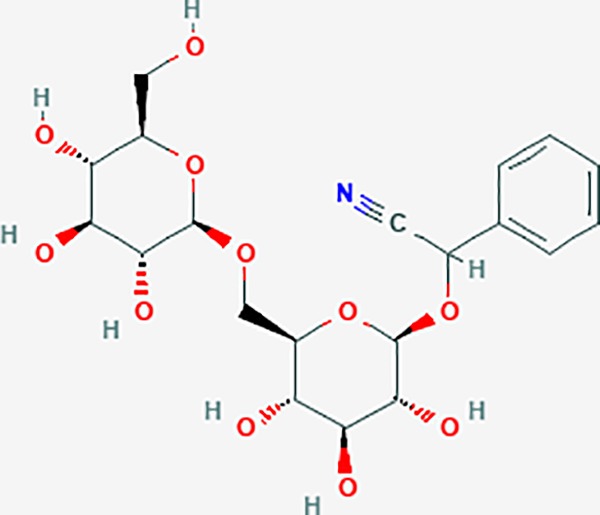	PTGS1,PTGS2,HSP90AB1,PIK3CG,PRKACA,NCOA2, CAMTA3
*Wolfiporia extensa*(Peck) Ginns (Fuling)	poricoic acid A*	5471851	C31H46O5	30.61	0.76	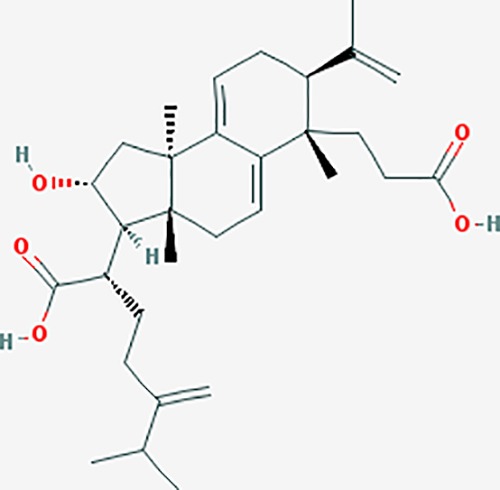	TOP2A,CYP2A6,CYP3A4,CTNNB1,PGR,CYP3A4,HDAC3,CYP1B1,CYP2A6,NR3C2,TOP2A
*Paeonia suffruticosa* Andrews (Mudanpi)	benzoylpaeoniflorin	21631106	C30H32O12	31.14	0.54	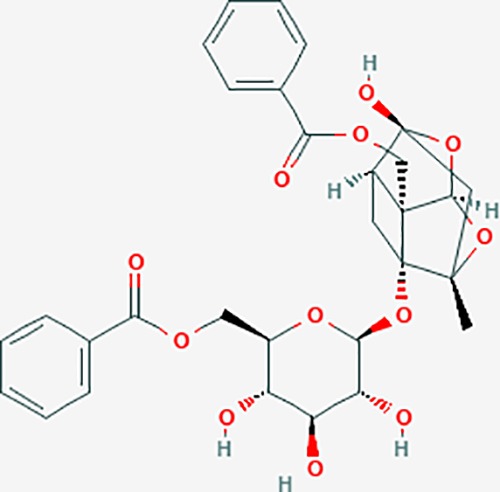	CYP2A6,CYP2B6,KDM1A,CYP1B1,MTOR,FABP2,HMOX1
	mairin/betulic acid	64971	C30H48O3	55.38	0.78	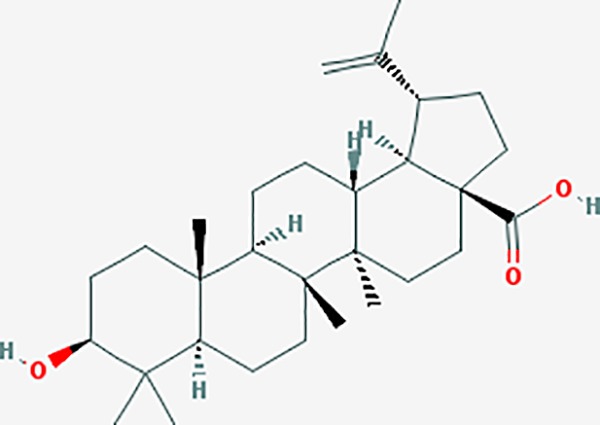	CYCS,LMNB1,SP1,PNLIP,NOS3,CASP3,AKT1,BIRC5,TOP1,TOP2A,PGR
	ursolic acid*	64945	C30H48O3	16.77	0.75	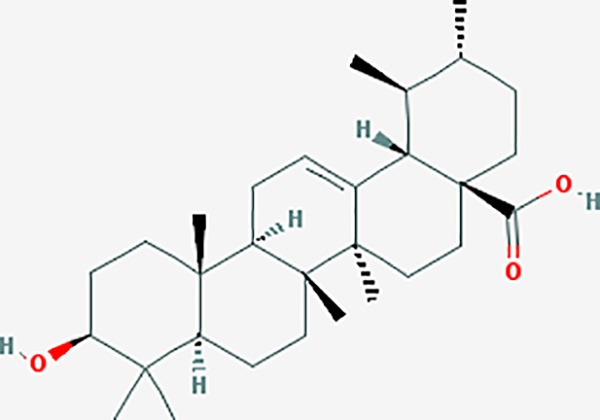	NR1I2,NTRK2,CYP2A6,CYP2C9,JAK3,CD40LG,FLT1,NR3C2,HSD17B1,PIK3CA,NOS2,CYP2D6,NR3C1,CYP3A5,MAPK10,GSK3B,CNR1,NOS3,CYP2C19,JAK2,FABP2,PDE5A,BRAF,CYP3A4,BMPR1B,NR5A1,IRS1,MAP3K7,MTOR,ALB,DNMT1,MAPK3,KDM1A,BCHE,MAPK8,RAF1,CFTR,CYP17A1,ADORA2A,VDR,NR5A2,EGFR,PLAT
*Paeonia lactiflora* Pall. (Shaoyao-Chishao/Baishao)	paeoniflorin	442534	C23H28O11	53.87	0.79	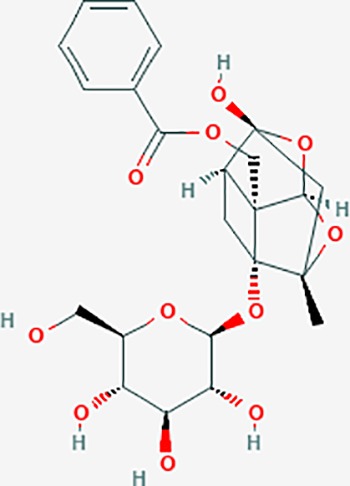	IL6,LBP
	paeonol*	11092	C9H10O3	28.79	0.04	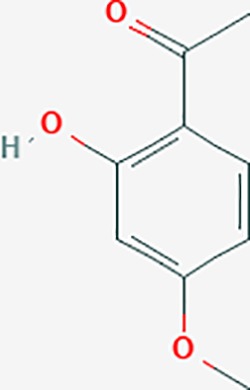	CHRM1,MAOB,PTEN,TYRP1,CHRM2,MAPK1,PTGS1,PTGS2,ADRA1A,ADRA1B,ADRA1D,ADRA2A,ADRA2B,ADRA2C,ADRB1,ADRB2,AHSA1,AKT1,BAX,BCL2,ADRA1A,ADRB2,AKT1,BCL2,ICAM1,MAOB,PTGS1,CHRM2,IKBKG,MAOA,MAOA,TNF,RELA,SLC6A2,SLC6A2,IL2
*Cyperus rotundus L*. (Xiangfu)	oleanoic acid*	485707	C30H48O3	12.84	0.34	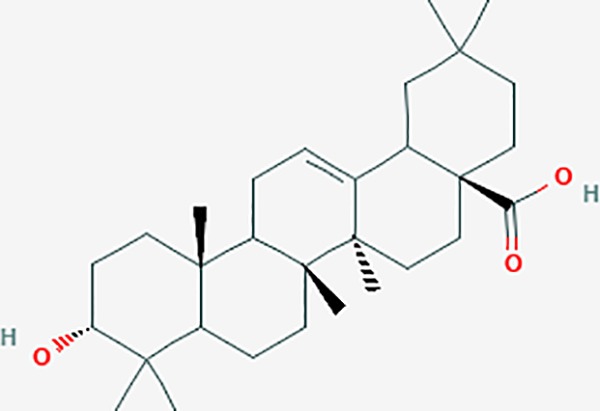	NR1I2,CYP2C9,NR3C2,CYP2A6,SERPINC1,PIK3CA,JAK3,NR3C1,PDE5A,HMOX1,VDR,CES1,MAPK10,CYP3A5,CYP3A4,NOS3,FABP2,BRAF,MTOR,CFTR,CDH1,CD40LG,CYP2C19,FLT1,DNMT3A,HDAC3
	kaempferol	5280863	C15H10O6	41.88	0.24	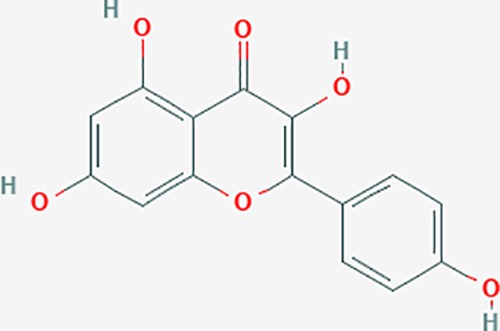	CYP1A1,PSMD3,SELE,CYP1B1,F2,GABRA2,HSP90AB1,NCOA2,NR1I3,CHRM2,DPP4,MMP1,PTGS2,CYP3A4,ACHE,ADRA1B,AHR,AHSA1,AKR1C3,AKT1,ALOX5,AR,BAX,BCL2,CYP1A2,GSTM1,NR1I2,PPARG,ICAM1,SLPI,CHRM1,PIK3CG,DIO1,F7,IKBKB,NOS2,PGR,PPP3CA,PRSS1,SLC2A4,STAT1,TNF,XDH,CASP3,GSTM2,GSTP1,JUN,MAPK8,NOS3,RELA,PRKACA,VCAM1,GABRA1,HAS2,HMOX1,INSR,PTGS1
*Curcuma phaeocaulis* Valeton (Ezhu)	curcumin*	969516	C21H20O6	5.15	0.41	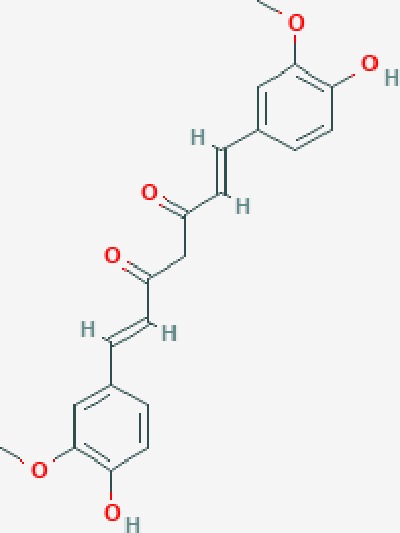	SULT1A1,CNR1,MMP1
	bisdemethoxycurcumin	5315472	C19H16O4	77.38	0.26	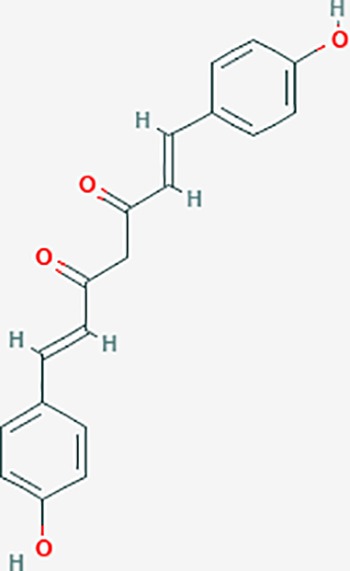	SULT1A1,COMT,MMP1,NT5E
	isocurcumenol*	5255901	C15H22O2	97.67	0.13	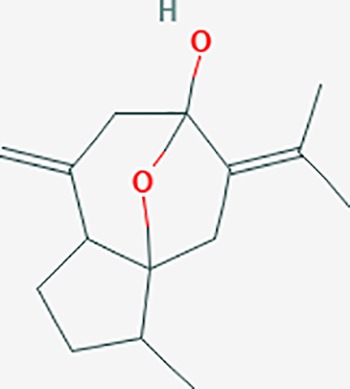	GRIK2,CHRM3,CHRM1,PTGS2,CHRM2,GABRA1,CHRNA7,GABRA6
	β-elemene*	6918391	C15H24	25.63	0.06	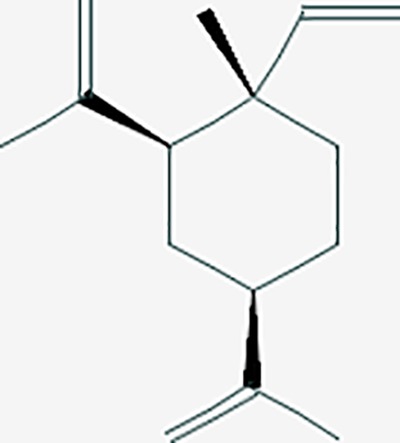	PTGS2,GABRA2,RXRA,NET,CHRM2,GABRA1,GABRA6,PTGS1,CHRM3,CHRM1,ADRA1A,CHRNA7,NCOA2,GABRA5,BCL2,CDKN3,EIF3L,RB1,TP53,TEP1,RUNX1T1,CRK2,CCNB1,RHOA
	β-caryophyllene*	5281515	C15H24	29.70	0.09	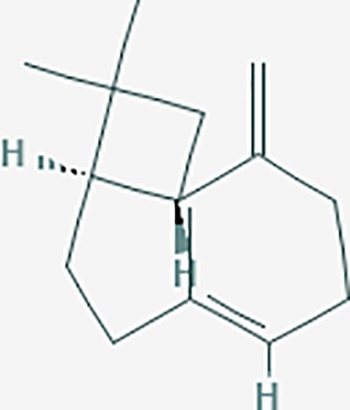	PTGS1,CHRM3,CHRM1,PTGS2,GABRA2,RXRA,CHRM2,ADRA1B,CHRNA2,GABRA1,NCOA2,GABRA6,NET,ADRA1A,SLC6A2,IL6
	curcumol*	14240392	C15H24O2	109.64	0.13	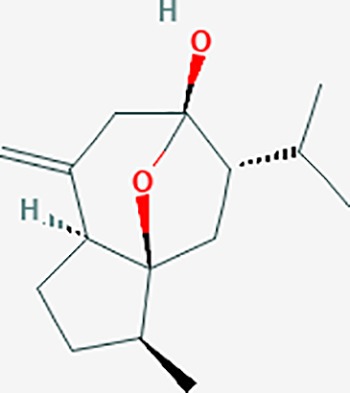	PGR,NR3C1,CHRM3,CHRM2
	γ-elemene*	6432312	C15H24	23.79	0.06	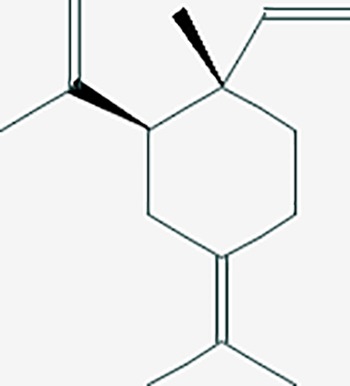	CHRM2,PTGS1,PTGS2,RXRA,ADRA1A,RXRA,GABRA2,GABRA1,GABRA6,PTGS1,CHRM3
*Corydalis Corydalis yanhusuo* (Y.H.Chou & Chun C.Hsu) W.T.Wang ex Z.Y.Su & C.Y.Wu (Yanhusuo)	corydaline	101301	C22H27NO4	65.84	0.68	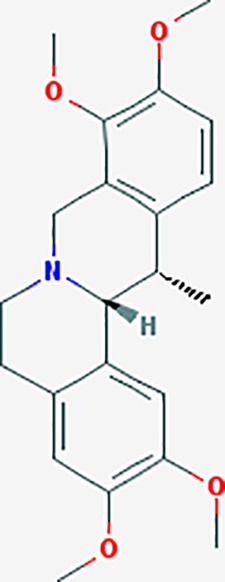	SLC6A2,CHRM1,DRD1,DRD2,OPRM1,CHRM4,RXRA,OPRD1,SLC6A4,TOP2A,CHRM3,ADRA1B,ADRA1D,ADRA2B,ADRB1,ADRB2,CA2,HTR2A,HSP90AB1,KCNA4,RXRB
	coptisine	72321	C19H14ClNO4	30.67	0.86	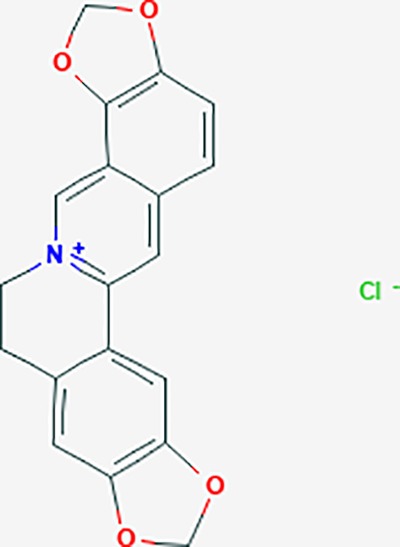	KCNA4,PTGS2,PTGS1,ADRB1,AR,NOS2,PRSS1,NOS3,ESR1
	berberine	12456	C20H18ClNO4	36.86	0.78	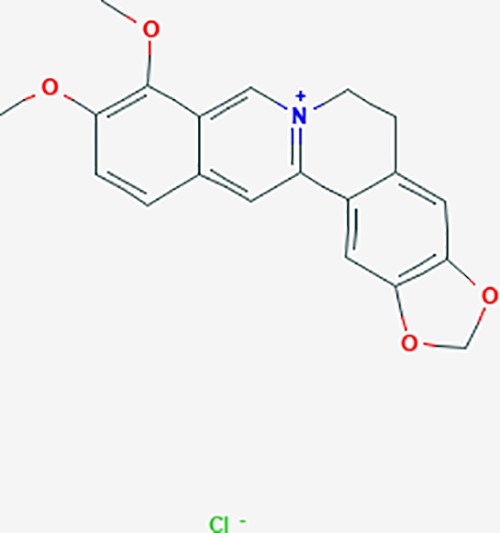	F10,PTGS2,RXRA,PRKACA,NCOA2,ADRB1,ADRB2,AR,NOS2,PRSS1,HSP90AB1,ESR1,KCNA4,NOS3
	dehydrocorybulbine	101879963	C21H22NO4+	46.97	0.63	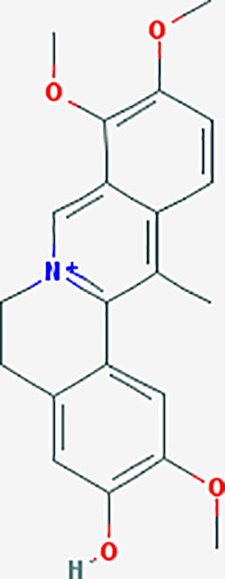	CHEK1,NCOA2,PTGS2,ESR1,KCNA4,MAPK14,RXRA,ADRB1,AR,NOS2,PRSS1,PTGS1,PIM1
	stylopine/tetrahydrocoptisine	6770	C19H17NO4	48.25	0.85	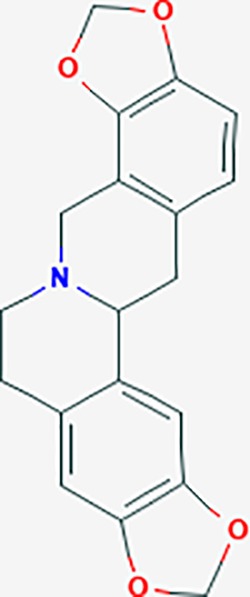	CHRM4,CHRM1,CHRM3,RXRA,OPRD1,ADRA1B,ADRA1D,ADRB1,ADRB2,HTR2A,OPRM1,HTR3A,SLC6A2,PTGS1,PTGS2
	canadine	34458	C20H21NO4	55.37	0.77	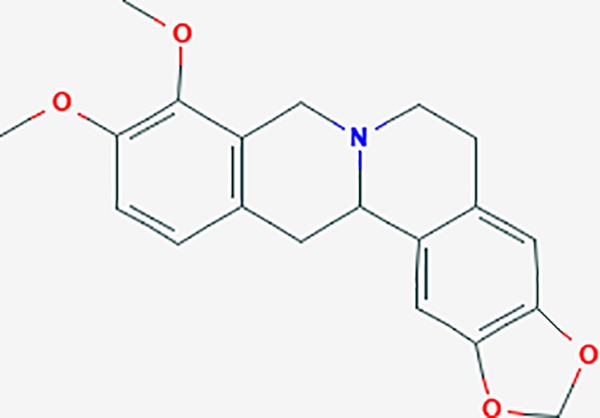	CHRM1,DRD1,HSP90AB1,OPRD1,OPRM1,RXRA,SLC6A4,HTR3A,PTGS1,CHRM4,CHRM2,CHRM3,ADRA1A,ADRA1B,ADRA1D,ADRA2C,ADRB1,ADRB2,HTR2A,KCNA4,SLC6A2,F10,KCNMA1,PRKACA
	capaurine	94149	C21H25NO5	62.91	0.69	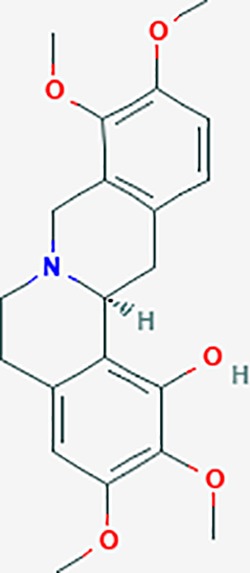	CHRM1,DRD1,KDR,OPRD1,OPRM1,SLC6A4,KCNA4,CHRM3,ADRA1B,ADRA1D,ADRB1,ADRB2,CA2,HTR2A,PTGS1,RXRA,SLC6A2,TOP2A,CHRM4,F10,HSP90AB1,KCNMA1,NOS3, RXRB
	palmatine	19009	C21H22NO4 +	64.60	0.65	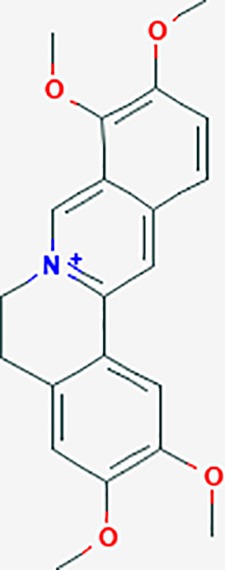	PIM1,CDK13,NCOA2,RXRA,PTGS2,ESR1,HSP90AB1,ADRB1,ADRB2,AR,ESR2,F7,NOS2,PRSS1,KCNA4,NOS3,PRKACA,PTGS1
	(S)-Scoulerine	439654	C19H21NO4	32.28	0.54	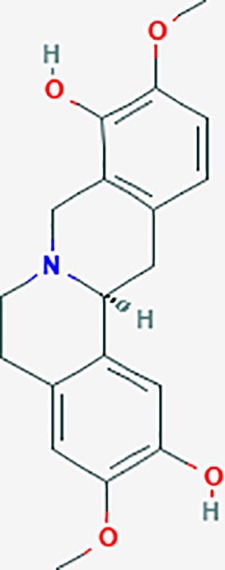	KCNA4,RXRA,CHRM1,DRD1,OPRM1,PTGS2,CA2,PTGS1,CHRM4,F10,CHRM2,CHRM3,NCOA2,OPRD1,ADRA1A,ADRA1B,ADRA1D,ADRA2A,ADRA2B,ADRA2C,ADRB1,ADRB2,F7,HSP90AB1,HTR2A,PDE3A,SLC6A2,TOP2A,SLC6A4
*Cinnamomum cassia* (L.) J.Presl (Guizhi)	syringaresinol*	100067	C22H26O8	3.29	0.72	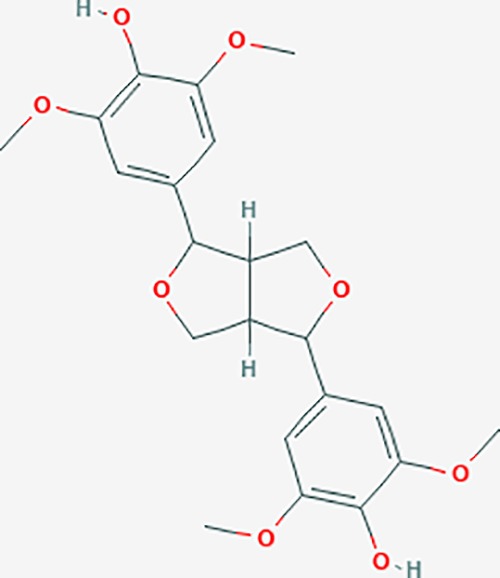	KCNA4,ADRB1,F10,PTGS2,TOP2A,NCOA2,CAMTA3,HSP90AB1
*Carthamus tinctorius* L. (Honghua)	hydroxysafflor yellow A*	6443665	C27H32O16	4.77	0.68	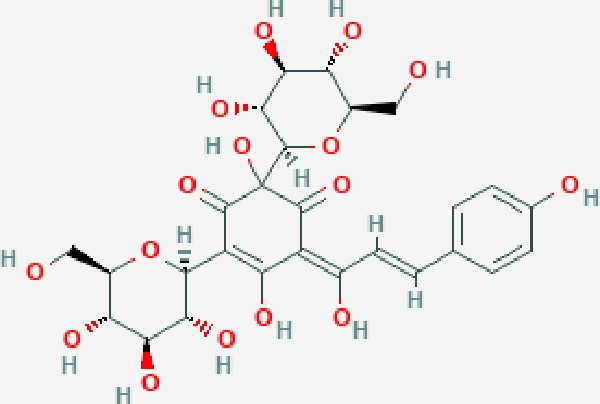	NR3C1,SIRT1,CAT
	rutin*	5280805	C27H30O16	3.20	0.68	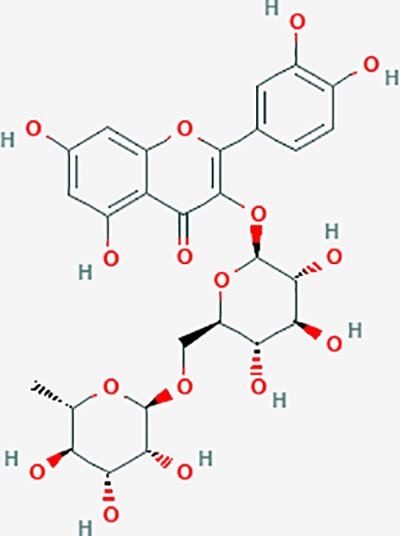	TOP2A,NFKB3,TNF,IL6,CASP3,POR,SOD1,CAT,IL-1beta,CXCL8,PRKCB,ALOX5,HMGCR,HAS2,GSTP1,DIO1,C5AR1,INS,FCER2,ITGB2,TBXA2R
*Astragalus mongholicus* Bunge (Huangqi)	Isoastragaloside I*	13996685	C45H72O16	46.79	0.11	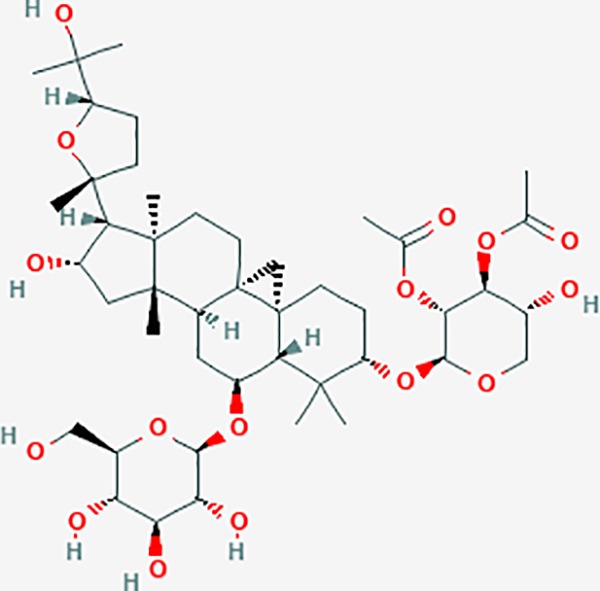	CYP17A1,CYP2D6,NR1I2,NOS3,CYP3A4,CYP3A5
	quercetin	5280343	C15H10O7	46.43	0.28	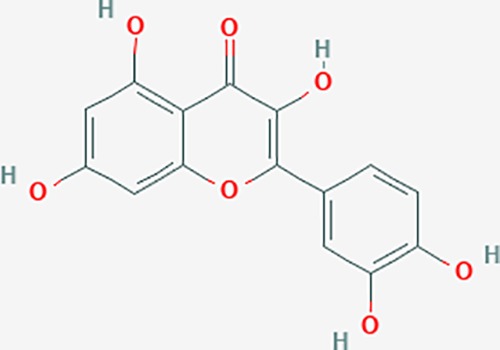	CASP8,CD40LG,CYP1A1,DPP4,IRF1,KCNA4,MMP2,NPEPPS,POR,PPARD,SELE,SOD1,CASP3,CDKN3,CHUK,CLDN4,COL1A1,COL3A1,CRP,CTSD,CXCL10,CXCL11,DIO1,EGFR,EIF3L,ELK1,F10,F2,FOS,GABRA1,GSTM2,HIF1A,HK2,HSP90AB1,IGF1R,IL10,IL6,JUN,MAOB,MPO,NCF1,NCOA2,PCOLCE,PLAT,PON1,PRKACA,PRKCB,PTEN,PTGER3,PTGS1,PTGS2,RXRA,TGFB1,TOP2A,E2F2,INSR,MMP1,THBD,CXCL2,HSPA5,HSPB1,MMP3,NFE2L2,PIK3CG,PPARG,CYP1B1,NOS3,RUNX2,TP53,IFNG,ABCA2,ACACA,ACHE,ACPP,ADRB1,ADRB2,AHR,AHSA1,AKR1B1,AKT1,ALOX5,AR,BAX,BCL2,BCL2L1,BIRC5,CCNB1,CYP3A4,IL1R1,IL2,MMP9,PLAU,PRKCARELA,CXCL8,ICAM1,IL1α,MGAM,ODC1,EGF,F7,GJA1,MAPK1,NR1I3,CASP9,CCL13,ERBB2,ERBB3,F3,HSF1,IKBKG,IL1b,NQO1,NR1I2,PARP1,PPARA,PRSS1,PSMD3,RAF1,RASA1,RASSF1,RASSF5,RB1,SERPINE1,SLC2A4,SPP1,STAT1,SULT1E1,TNF,VEGFA,XDH,CYP1A2,GSTM1,GSTP1,HAS2,RUNX1T1,CCND1,CHEK2,VCAM1,E2F1,HMOX1,NFKB3
	formononetin	442813	C22H22O9	66.39	0.21	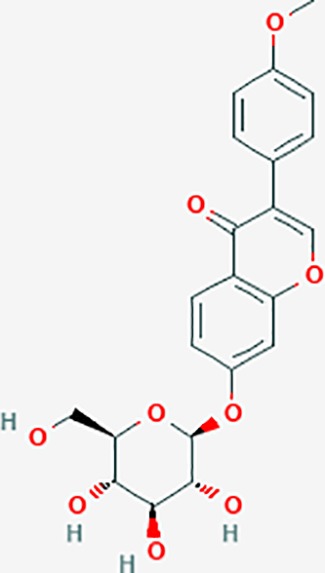	ACHE,ADRA1A,ADRB2,AR,ATP5F1B,CCNA2,CDK13,GSK3B,NOS2,PPARG,PRSS1,CHEK1,ESR1,HSP90AB1,IL4R,PRKACA,PTGS1,PTGS2,SLC6A4,DPP4,HSD3B1,JUN,MAOB,MAPK14,PKIA,PIM1,CHRM1,ESR2,F2,PDE3A,SIRT1,NOS3,RXRA,SLC6A2
	calycosin	5280448	C16H12O5	47.75	0.24	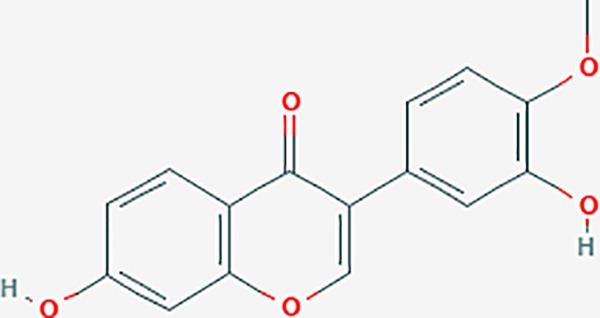	HSP90AB1,NOS2,ESR2,MAPK14,PRKACA,PTGS1,PTGS2,DPP4,GSK3B,CCNA2,CDK13,CHEK1,NCOA2,PIM1,PPARG,PDE3A,ADRB2,AR,ESR1,PRSS1,RXRA
*Angelica sinensis* var. *wilsonii (H.Wolff) Z.H.Pan & M.F.Watson* (Danggui)	stigmasterol	5280794	C29H48O	43.83	0.76	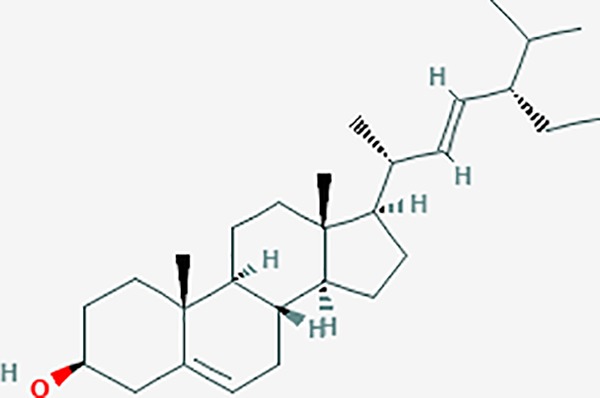	ADH1C,ADRA1A,ADRA1B,ADRA2A,ADRB1,ADRB2,AKR1B1,HTR2A,PTGS1,CHRM1,CHRM2,CHRM3,CHRNA7,GABRA1,IGHG1,NCOA2,NCOA2,PGR,PRKACA,PTGS1,PTGS2,RXRA,PLAU,ADRB2,MAOB,LTA4H,MAOA,NR3C2,NR3C2,SLC6A2
	ferulic acid	445858	C10H10O4	39.56	0.06	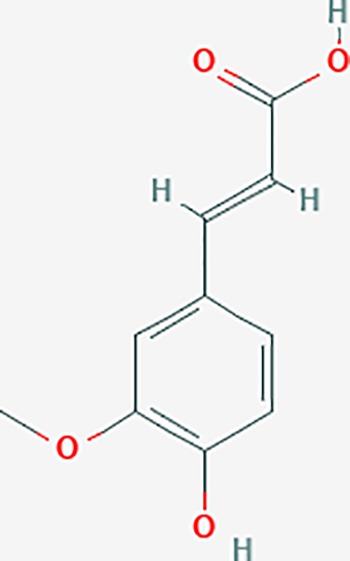	PTGS1,PTGS2,NOS3,ADRA2A,NET,ADRA2B,SLC6A2,ADRB2,LTA4H,MAOB,MAOA,PRKACA,CHRM2
*Conioselinum anthriscoides* ‘Chuanxiong' (Chuanxiong)	myricanone	161748	C21H24O5	40.60	0.51	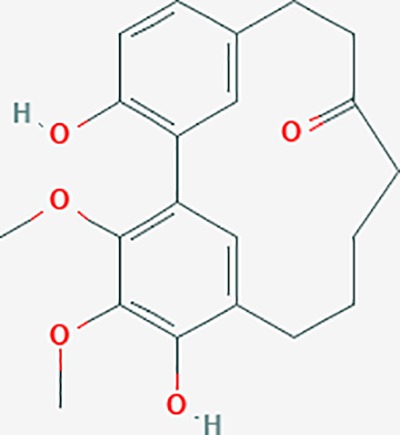	NOS2,PTGS1,F2,KCNA4,ESR1,AR,ADRB1,PPARG,PTGS2,F7,KDR,RXRA,PDE3A,ADRB2,ESR2,DPP4,MAPK14,GSK3B,HSP90AB1,CDK13,CHEK1,IGHG1,PIM1,CCNA2

*These compounds were predicted with low oral bioavailability (OB) and drug-likeness (DL) values in database, but could have potential therapeutic value from literature and network pharmacology.

The major compounds and targets network were showed in **Figure 8B**.

## Discussion

In this study, data mining and network pharmacology were used to study the rules and potential mechanisms of Chinese medicine in endometriosis. Our research shows that the compatibility of TCM is favorable, and the herbs currently used are mainly blood stasis drugs; the mechanism of action entails multiple pathways. Chinese medicine could provide a new source of screening for potential therapeutic drugs. Such Chinese herbs could also be used to treat other diseases, especially those associated with pain.

### Guidelines of Chinese herbs in Endometriosis

Endometriosis is the most common cause of secondary dysmenorrhea. Blood stasis syndrome (BSS) is an important associated pathology in Chinese medicine. Based on the data mining results, most of the common prescriptions for the treatment of endometriosis originate from classic prescriptions in ancient Chinese treatment of dysmenorrhea. These discovered herbs combinations and prescriptions could provide guidance for clinical treatment. The use of data mining methods to screen the core prescriptions of TCM also provides a new method for discovery of TCM.

Machine learning and deep learning methods provide a new method to extract informative medicinal and pharmaceutical research (Liang et al., 2019). Data mining could provide a way to explore the empirical knowledge to promote the development of Chinese medicine from individualized empirical knowledge to large-scale evidence-based medicine (Zhou et al., 2010). The “*Framework for Automated Knowledge Graph Construction Towards Traditional Chinese Medicine*” in our work could also aid the discovery of pharmaceutic rule of Chinese medicines. These tools are crucial to establish the effectiveness of Chinese medicine/herbs in endometriosis clinical treatment.

We selected some commonly used herbs and HPs to provide a source for network pharmacology. To provide better clinical evidence, the quality of modern clinical research in Chinese medicine is in urgent need of improvement. The mechanisms of animal-derived Chinese medicine, as well as some herbal Chinese medicines, need further analysis and exploration.

### The Major Mechanism of TCM

In this study, we discovered potential compounds for the treatment of endometriosis. Studies on phytochemicals have been mostly focused on polyphenolic compounds, sesquiterpenes, terpenoids, flavonoids, alkaloids, polysaccharides, and steroid glycosides. The molecular biological approach has further helped to elucidate the mechanisms of HPs and their pharmacological actions. TCM has advantages in the regulation of endometriosis pain, can directly regulate neurotransmitters, and indirectly regulate pain by modulating inflammation and immunity.

Chinese herbs may interfere with the development of endometriosis, during the processes of invasion, adhesion, angiogenesis, immune inflammation, and oxidative stress, among other processes, such as inhibition of the epithelial-mesenchymal transition. The results of the current *in vivo* and *in vitro* experiments are consistent with the analysis of network pharmacology. This suggests that TCM could reduce ectopic lesions and inhibit the development of endometriosis by intervening inflammation, immunity, and inducing apoptosis.

The potential effective compounds based on network pharmacology have also been validated in experiments. The effects of compounds in endometriosis treatment typically induce apoptosis to reduce lesions through various pathways. For example, nerolidol could reduce the average volume of lesions in rats with endometriosis (Melekoglu et al., 2018). Beta-caryophyllene could suppress the growth of endometrial implants in a rat model of endometriosis, without affecting fertility by inducing apoptosis in the luminal epithelium of cysts and in the endothelial cells of blood vessels (Abbas et al., 2013). Tanshinone IIA could also reduce the expression of 14-3-3ζ in ectopic endometrial stromal cells but has no effect on apoptosis (Wan et al., 2015).

To analyze the role of various modules, we discuss only the main modules of Chinese medicine treatment in endometriosis.

#### The Pain Relief Function

Increased levels of proinflammatory cytokines have been observed from endometriotic lesions, based on examination of the peritoneal fluid of women with endometriosis. These cytokines are also responsible for excessive sensory innervation and the development of chronic pelvic pain (Kobayashi et al., 2014; Zhang J. et al., 2015). The results showed that Chinese herbs could reduce pain associated with the regulation of neurotransmitters (5-hydroxytryptamine, dopamine, and nervous system associated pathways (Wang et al., 2018).

Compounds such as fumarine isocorypalmine fagarine I, in *C. anthriscoides ‘Chuanxiong’* and *C. yanhusuo* could reduce pain *via* HTR3A, HTR2C, HTR2A. Furthermore, PTGS, also known as cyclooxygenase, is the key enzyme in prostaglandin biosynthesis. It acts both as a dioxygenase and a peroxidase, and may be a core target of TCM for pain. Prostaglandin E2 (PGE2) production by PTGS2/cyclooxygenase 2 (COX-2) is known to be a critical inflammatory factor in endometriosis-associated pain (Sacco et al., 2012).

Various volatile oils, especially terpenes and derivative compounds in Chinese herbs could relieve endometriosis associated pain by regulating prostaglandins and opioidreceptors, in a similar manner to steroidal antiinflammatory drugs (Sacco et al., 2012) or prescribed opioid drugs, cannabis, and cannabinoids (Guimaraes et al., 2014). Chinese medicine compounds could also alleviate pain by regulating inflammatory, immunity, and other related neuronal receptor pathways. For example, *C. myrrha could exert analgesic effects, depending on the presence of a biologically active sesquiterpene with a furandadiene skeleton* (Shen et al., 2012). C*orydalmine*, L*-*tetrahydropalmatine, and demethylcorydalmatine in *Curcuma aromatica Salisb*. (*Curcumae Radix*,Yujin) *and Curcuma phaeocaulis* Valeton(*Curcumae Rhizoma*,Ezhu) alleviate mechanical hyperalgesia in models of chronic inflammatory and neuropathic pain in mice (Zhou H. et al., 2016).

In addition, BDNF may be a key factor in chronic pelvic pain of endometriosis, and is an effector of estrogen in endometrial cells, thus affecting cell growth (Dong et al., 2017; Ding et al., 2018). Furthermore, BDNF is the potential target of oleic acid, based on the results of network pharmacology.

Chinese medicine could also relieve pain by regulating the inflammatory immune processes and neurotransmitters. Ferulic acid could significantly attenuate vincristine-induced behavioral alterations, as well as electrophysiological and histopathological changes (Vashistha et al., 2017). *C. yanhusuo* effectively attenuates neuropathic pain, and could exhibit prominent dopamine receptor antagonistic properties(Wang et al., 2016). The herbs that can eliminate blood stasis could also regulate pain through multiple cross-talk pathways. Based on the results of data mining, we found that Chinese medicines often include several blood stasis-associated herbs that can also relieve pain. Further screening of pain-related HPs and effective combinations of compounds could identify new drugs for the treatment of endometriosis and other diseases associated with pain.

#### Regulatory Hormone Function

Estrogen-mediated associated signaling pathways have important implications for the pathogenesis of endometriosis (Tang et al., 2019). Estrogenreceptor (ER) β could inhibit TNF-α-induced apoptosis and interact with components of the cytoplasmic inflammasome, and finally enhances epithelial-mesenchymal transition (Han et al., 2015). Moreover, we found that estrogen pathway and multiple pathways have cross-talk. Estrogen pathway is related to apoptosis and neural and inflammatory regulatory pathways. Estrogen also play an important role in the regulation of the central serotoninergic system in pain (Paredes et al., 2019). Aromatase is considered a key therapeutic target for regulating the biosynthesis of local estrogen in endometriosis.

At present, the main targets of drug treatment include aromatase, steroid hormone biosynthesis, the estrogen signaling pathway, and drug metabolism-cytochrome P450 pathway. *A. sinensis* could act in conjunction with estrogen signaling pathways. Genistein could regulate estrogen receptor-α and estrogen receptor-β, and reduce the TNF-α, IL-6, VEGF, and HIF-1α levels in ectopic lesions in the endometriosis murine model (Sutrisno et al., 2018). Chinese medicine may play a synergistic role in the regulation of estrogen and serotonin. The regulatory effect of Chinese herbs/compounds on the estrogen receptor still needs further experimental verification, as its agonistic and inhibitory effects cannot be determined through network pharmacology.

#### The Antiinvasive, Antiadhesion, and Angiogenesis Functions

Invasion, adhesion, and angiogenesis are the core mechanisms of endometriosis (Gagne, 2003), which was also the core module of our Chinese medicine intervention. Various molecular compounds (genistein, oleanolic acid, luteolin, kaempferol, and ursolic acid) have been involved in antiinvasive activity [*via* decreased expression of intercellular adhesion molecule-1 (sICAM-1), vascular cell adhesion molecule-1 (VCAM-1), and matrix metalloproteinases (MMPs)] associated with endometriosis (Kuessel et al., 2017). For example, formononetin, an isoflavone from *A. membranaceus*, showed potential effects on the induction of apoptosis, and suppression of migration and invasion (Zhang J. et al., 2018).

The VEGF pathway is a core pathway in Chinese medicine to regulate angiogenesis. Formononetin also could also promote the proliferation and migration of human umbilical vein endothelial cells (HUVECs) by upregulating VEGF and activating extracellular signal-regulated kinase (ERK) (Liang et al., 2018). Tanshinone IIA could induce angiogenesis by inducing the VEGF/VEGFR2 pathway and CD146 (melanoma adhesion molecule) and regulating angiogenic function in HUVECs (Zhang et al., 2016). Curcumin could reduce ectopic endometrial microvascular density (MVD) and VEGF protein expression in an endometriosis rat model (Zhang et al., 2011). Traditional Chinese herbal medicine for the promotion of blood circulation and removal of blood stasis may become a crucial target for antiangiogenesis in endometriosis.

#### Antiinflammatory Effect and Regulation of Immune Function

The results of network pharmacology and related molecular biology experiments suggest that the toll-like reporter signal pathway and NF-κB signal pathway could be core pathways of TCM in inflammatory immune regulation. Differential expression of antiinflammatory cytokines (IL-10, TGF-β, TLR4 and NF-κB) occurs in women with endometriosis, and this can promote survival, growth, invasion, differentiation, angiogenesis, and immune escape of the endometriotic lesions (Zhou et al., 2019). Rutin, quercetin, quercitrine, catechin, kaempferol, epicatechin, caffeine, and theobromine *are also active compounds that are present in several other types of Chinese herbs*. These compounds could form complexes with NF-κB and IκB in the cytoplasm (Yuniwati et al., 2018). Curcumin, epigallocatechin gallate, luteolin, quercetin, resveratrol, caffeic acid phenethyl ester, xanthohumol, genistein, and berberine could inhibit TLR4 activation (Chen C. et al., 2018).

#### Regulating the Kinase Signalling Pathways Function

Imbalance of protein kinases can lead to uncontrolled cell proliferation by stimulating the tumor formation process, leading to kinase-dependent tumor growth. Chinese medicine could play an active role in regulating the kinase signaling pathways. These phenomena are also related to the occurrence and development of endometriosis. The kinase signaling pathway represents a viable target for the treatment of endometriosis (McKinnon et al., 2016). Major signaling pathways, such as the PI3K-Akt signaling pathway, generally promote survival through the inhibition of proapoptotic factors and activation of antiapoptotic factors.

The PI3K-Akt-mTOR pathway is one of the major signaling pathways in endometriosis. Furthermore, mTOR is a key kinase downstream of PI3K-Akt, which regulates tumor cell proliferation, growth, survival and angiogenesis. The expression of the PI3K-Akt pathway (PI3K, PTEN, Akt, and p-Akt) is related to the severity of endometriosis (Madanes et al., 2019). Natural pentacyclic triterpenoids and their semisynthetic derivatives are involved in cell cycle arrest, apoptosis, and autophagy, triggered by the effects of triterpenoids on TGF-β and HER cell surface receptors. They also induce the PI3KAkt-mTOR and IKK/NF-kB signaling pathways, and the STAT3 and MAPK pathways (Markov et al., 2017). Luteolin, could inhibit the development of endometriosis by regulating the expression of PI3K/Akt signaling, the MAPK signaling pathway, and CCNE1 protein in human VK2/E6E7 and End1/E6E7 cell lines and in the endometriosis mouse model (Yi et al., 2018). Naringenin could induce apoptosis in human endometriosis cells by regulating the MAPK and Akt pathways (Park et al., 2017). Research on the mechanism of TCM treatment in endometriosis based on the kinase pathway can also be a main direction for future research.

#### The Anticancer Function

Based on the results of gene enrichment analysis of endometriosis, we can also determine that the related genes are associated with many cancer pathways. Endometriosis shows tumor-like behavior. Neoplasia is also a rare but significant complication of endometriosis. It usually occurs in the ovaries, and can often arise in younger patients (Matias-Guiu and Stewart, 2018). Curcumol could inhibit MMP-9 *via* the c-Jun N-terminal kinase (JNK) 1/2 and NF-κB signaling pathways in breast cancer cells (Ning et al., 2016). Ellagic acid is a naturally occurring polyphenolic compound with strong antioxidant and anticancer properties, which could also inhibit tumor cell migration, extracellular matrix invasion, and angiogenesis (Ceci et al., 2018).

β-elemene, a compound extracted from *C. phaeocaulis, C. cassia*, *and C. myrrha*, has proven broad-spectrum antitumor activity and is an effective treatment for several types of tumors (Bi et al., 2018). β-caryophyllene and β-caryophyllene oxide-based natural compounds possess anticancer and analgesic properties (Fidyt et al., 2016). Betulinic acid and betulin are lupane-type pentacyclic triterpenoids with multiple bioactivities, particularly antitumor effects (Zhang D. et al., 2015).

Phytosterols, such as stigmasterol, β-sitosterol, and spinasterol, which are closely related to endometriosis-related targets, could exert anticancer effects by reducing cell cycle progression, inducing apoptosis, and inhibiting tumor metastasis (Shahzad et al., 2017). Hydroxysafflor yellow A could inhibit hepatocellular carcinoma through the inhibition of MMP-2, MMP-9, and p38MAPK signaling pathways in HepG2 cells (Zhang J. et al., 2019). These tumor-related targets are closely related to endometriosis and are the core targets of current research in endometriosis treatment.

**Figure 9:**
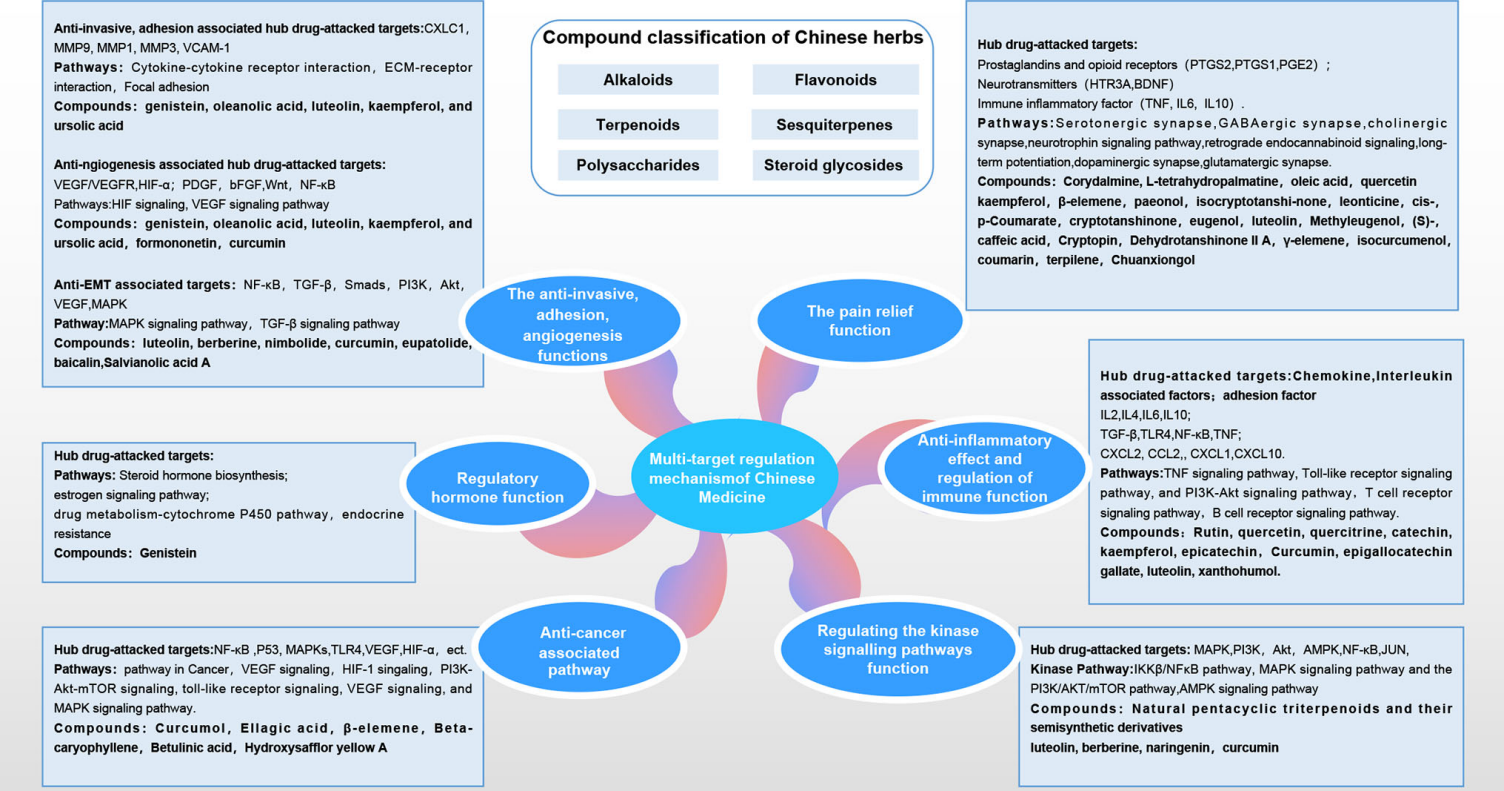
Potential mechanism of action of Chinese herbs/compounds in endometriosis. Based on the results of our network pharmacological analysis, polyphenolic compounds, sesquiterpenes, terpenoids, flavonoids, alkaloids, polysaccharides, and steroid glycosides are commonly used for the treatment of endometriosis. Traditional Chinese medicine for treating endometriosis includes regulating estrogen receptors, regulating nerve-related receptors, regulating inflammatory and immunity, inhibiting invasion, adhesion, and angiogenesis, affecting the kinase signalling pathways. The Gene Ontology (GO) and Kyoto Encyclopedia of Genes and Genomes (KEGG) enrichment analyses showed that the treatment of endometriosis with Chinese medicine is regulated by multiple cross-talk pathways. Such treatments could act directly on neurotransmitter-related pathways and the estrogen pathway, which is similar to the action of conventional treatment. Chinese medicine also could regulate adhesion, invasion, and angiogenesis by regulating PI3K-Akt signaling, toll-like receptor signaling, NF-κB signaling, and the MAPK signaling pathway, among others.

Based on the results of gene enrichment, we could more comprehensively consider the regulatory effects of Chinese medicine on neurotransmitter-associated pathways, focal adhesion, PI3K-Akt-mTOR signaling, toll-like receptor signaling, VEGF signaling, and MAPK signaling pathway, among others, which may be used to treat the uterine endometriosis-associated core pathway. The mechanism of Chinese medicine in endometriosis is presented in **Figure 9**.

### The Combining Rule and Synergy Effects of HPs and Prescriptions

The formation of Chinese formulas composed of multiple herbs is not random. Data mining provides the basis for our discovery of drug combinations and core prescriptions. The effects of a combination of TCM compounds differ from those of a single natural compound and could play a synergistic role (Yang et al., 2014). HPs play a key role in the investigation of general herbal compatibility and their indispensable effects (Wang et al., 2012). Different compounds/herbs may regulate either the same or different target in various pathways, as well as regulate the enzymes and improve oral drug bioavailability. Systematic identification of the druggable interactions could provide a way to determine the effective mechanisms and new potential drugs for endometriosis treatment (Jiang et al., 2017).

*C. phaeocaulis* and *S. stoloniferum*(Ezhu and Sanleng), as a basic HP was first recorded as Sanleng Wan in “*Jingyan Liangfang*” during the Qing dynasty. The pairs possess extensive activities, including anticancer activity, antiinflammatory activity, and antithrombotic activity (Xu et al., 2015). This HP has shown therapeutic effects on hysteromyoma *via* the regulation of multiple metabolic pathways. This was demonstrated by UPLC-Q-TOF-MS analysis of a hysteromyoma rat model comprising 16 potential biomarkers from serum and 18 potential biomarkers from urine (Li et al., 2019).

The five herbs in GFW prescriptions appear to play different roles in complementary ways. In addition, GFW could regulate toll-like receptor signaling, TNF signaling, NF-kappa B signaling, neuroactive ligand-receptor interaction, and affect the occurrence and development of endometriosis based on network pharmacology. The pharmacological network functions of *C. ramulus* target proteins were focused on neurological, inflammatory disease, cancer, cellular growth and proliferation, cell signaling, and molecular transport. Cinnamon extract, active compounds in *C. ramulus*, could exert antiinflammatory effects by influencing the TLR2 and TLR4 signaling pathways (Schink et al., 2018). Major compounds of *P. lactiflora* Pall. have been classified as flavonoids, hydrolysable tannins (polyphenols), and monoterpene glycosides. Paeoniflorin could inhibit the plantar incision-induced microglia TLR4/matrix metalloproteinase (MMP-9)/MMP2/IL-1β signaling pathway and suppress postoperative pain in mice (Fan et al., 2018). Paeoniflorigenone could induce apoptosis and has antiproliferative effects in the cancer cell lines (Huang et al., 2017).Paeonol has antipyretic, analgesic, antitumor, antioxidation, antiinflammatory, and immunoregulatory properties (Zhang L. et al., 2019). Paeonol could regulate TLR4/MyD88/NF-κB signaling pathway in lipopolysaccharides-induced acute lung injury C57BL/6 mice (Wang et al., 2019).

These results suggest that the herbal combinations with GFW could have synergistic effects, and may induce apoptosis, antiinflammatory and regulate immunity through the Toll-like receptor signaling, NF-κB signaling pathway.

Other commonly used Chinese medicine prescriptions, such as the *ShaoFu Zuyu* decoction could reduce the size of ectopic lesions in rats with endometriosis, inhibit cell proliferation, promote apoptosis, and reduce microvessel density and HIF-1α expression (Huang et al., 2016). *Jiawei Foshou San*, composed of ligustrazine, ferulic acid, and tetrahydropalmatine, could inhibit the progression of endometriosis by regulating epithelial-mesenchymal transformation, based on network pharmacology and experimental verification (Tang et al., 2014; Chen Y. et al., 2018). Network pharmacology could facilitate the analysis of the roles of the synergism effect of compouds or Chinese medicine (Yuan et al., 2017).

Most Chinese herbs/natural compounds may work in a synergistic manner. More research should be focussing on combinatorial effects from HPs/compounds and not just single compounds in drug discovery for complex diseases (Thomford et al., 2018). TCM provides valuable resources for research on complex diseases such as endometriosis. We should adhere to the concept of HPs and prescriptions of TCM. While we still need to use data mining, computer-aided drug discovery and other methods to find more effective herb/medical plant/compound combinations.

This study presented the systemic pharmacological mechanisms of antiendometriosis herbal medicines. A well-designed herbal formula composed of several herbs may synergistically enhance the treatment of specific symptoms. These symptoms may be manifestations of the functional genome. Therefore, using data mining and network pharmacological methods to construct a suitable herbal formula and compound combination could have positive significance.

However, there are still several problems need to be solved. The methodological quality of clinical trials of Chinese herbs in endometriosis needs to be improved. More rigorous research is required to accurately assess the potential roles in endometriosis treatment. Some Chinese medicines are derived from plants of different family and genus. In addition, there are several kinds of names,such as plant names, latin names, and English names, etc., which makes it difficult to confirm medical plants. Accurate scientific nomenclature for plants is essential in the study of ethnopharmacology (Rivera et al., 2014). TCM databases, such as TCMSP, TCMID, TCM-MeSH, are still not comprehensively included in some medical plants/drug targets. Thus, our study used multiple databases to supplement the available information. Network pharmacology analysis needs more evidence to evaluate the potential pharmacological effects of compounds/herbs. We need to further establish comprehensive databases of Chinese medicine and natural compounds to improve compound-related information (Zhang R. et al., 2019).

We should also focus on the pharmacology/bioactivity of bioactive preparations (Heinrich et al., 2020). However, it is confusing that several compounds with low OB, DL in the database have reported many pharmacological values. Such as Salvianolic acid A (OB=2.96%) has several pharmacological actions in antithrombosis, antifibrosis (Xu et al., 2018). In the field of target analyses of network pharmacology, it is difficult to distinguish whether a compound has an effect of inhibition or agitation. The amounts of each bioactive compound in herbs and TCM formulas are very low and Complex Interactions are not fully understood (Eng et al., 2019).

Thus, regarding the compounds and gene targets in the network pharmacology database of Chinese medicine, and the large amount of available information, we still need to conduct more comprehensive evaluations of their therapeutic action based on the literature and in-depth *in vivo* and *in vitro* studies, as well as toxic or side effects. We also need to identify ways to improve the current methods. Chinese medicine research requires modernization, such as computational methods (computer-aided drug design) (Chen X. et al., 2018), metabolomics (Wang et al., 2017), mass spectrometry (Zhang A. et al., 2019), and new multiinformational-based profiling approaches integrating taxonomic or bioactivity data (Wolfender et al., 2019), which would present additional techniques by which new compounds can be discovered.

## Conclusions

Endometriosis is a common and difficult disease in gynecology. The mechanism of TCM in the treatment of endometriosis is realized through functional modules, such as inhibiting inflammation, enhancing the immune response, regulating angiogenesis-related pathways, inhibiting the epithelial-mesenchymal transition, and inducing apoptosis. The use of data mining combined with bioinformatics techniques could help us understand the associated targets and pathways networks, candidate genes in endometriosis, and the mechanisms of action of Chinese medicine. Combined techniques may also offer an efficient method of drug discovery and development from herbal medicines.

However, there are still several potential limitations in the study and need to improve. First,the mechanism of endometriosis needs to be further clarified based on molecular biology and multiomics technology. Moreover,the antagonistic or agonistic effects of compounds–targets pairs should be further clarified. It is essential to improve the methods in data mining and network pharmacology. Further *in vitro* or *in vivo* experiments should be performed to validate the predicted herbs/compounds and targets.

## Data Availability Statement

All datasets generated for this study are included in the article/**Supplementary Material**.

## Author Contributions

All authors were responsible for the study concept and design: WZ and JWu have contributed equally for this work. WZ drafted the paper. JWu help drafted the paper. WZ, JWu, and JWa participated in the literature search. WZ, JWu, and HW participated in data mining. WZ, JG, and TW participated in network pharmacology analysis. LC and XL supervised the study. All authors approved the final paper.

## Funding

This study is supported by the National Natural Science Foundation of China (81574008), the National Natural Science Foundation of China (61871141), Special Research Project for Traditional Chinese Medicine of Guangdong Hospital of TCM (NO. YN2016ML05), and Situ Yi Renowned Medical Heritage Studio.

## Conflict of Interest

The authors declare that the research was conducted in the absence of any commercial or financial relationships that could be construed as a potential conflict of interest.

## Supplementary Material

Supplementary Table 1 The validated information of major herbs, including location, used part, famliy, genus and medical plant reference.

Supplementary Table 2 The frequent item sets of major Chinese herbs.

Supplementary Table 3 KEGG pathways of endometriosis associated genes.

Supplementary Table 4 The major compounds and targets from of major Chinese herbs for endometriosis treatment.

Supplementary Table 5 The Chinese herbs associated targets names.

Supplementary Table 6 The major KEGG pathways of major Chinese herbs, herbs pairs and prescription.

Supplementary Table 7 The targets of endometriosis-associated genes that coincided with those of Chinese herbs.

Supplementary Table 8 The GO enrichment of endometriosis-associated genes that coincided with Chinese herbs.

Supplementary Table 9 Drug targets of endometriosis conventional treatment.

Supplementary Table 10 The potential compounds and targets.

Supplementary Figure 1 The PPI network of the major endometriosis genes.

Supplementary Figure 2 The cross-talk pathways network of Chinese herbs in endometriosis treatment.

## Abbreviations

ADMET, absorption, distribution, metabolism, excretion and toxicity; BP, biological process; BSS, blood stasis syndrome; CC, cellular component; DEGs, differentially expressed genes; DL, drug-likeness; EMT, epithelial-to-mesenchymal transition; GO, Gene Ontology; KEGG, Kyoto Encyclopedia of Genes and Genomes; MCODE, molecular complex detection; MF, molecular function; OB, oral bioavailability; OMIM, Online Mendelian Inheritance in Man; PPI, protein-protein interactions; RCSB PDB, Research Collaboratory for Structural Bioinformatics Protein Data Bank; TCMSP, Traditional Chinese Medicine Systems Pharmacology; TTD, Therapeutic Target Database; UHPLC-QTOFMS, ultrahigh-performance liquid chromatography tandem quadruple time of flight mass spectrometry.
