# Supplementary material for: Modular Characteristics and Mechanism of Action of Herbs for Endometriosis Treatment in Chinese Medicine: A Data Mining and Network Pharmacology–Based Identification
Source: Front Pharmacol. 2020 Mar 6;11:147. doi: 10.3389/fphar.2020.00147 (PMC7069061; doi:10.3389/fphar.2020.00147)
Supplement: Supplementary Table 1 — The validated information of major herbs, including location, used part, famliy, genus and medical plant reference. [file Table_1.pdf]

Table 1: The validated information of major plants/herbs, including location, used part,famliy,genus and medical plant references.

TCM name	Species Name/Scientific Name	Botanical Documentation	Location	Used part	Family	Genus	Medical plant reference
Ezhu	1.*Curcuma phaeocaulis* Valeton [Zingiberaceae]<br>2.*Curcuma zedoaria (Christm.) Roscoe* [Zingiberaceae]	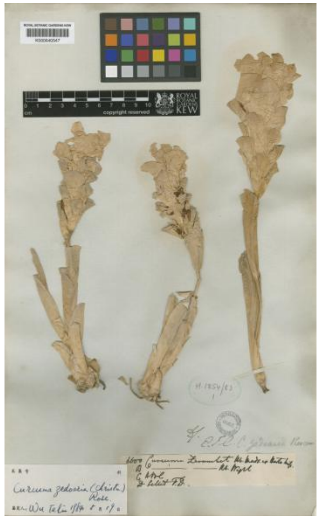	1.Native to: China South-Central, Jawa, Vietnam Introduced into: China Southeast<br>2.Assam, Bangladesh, East Himalaya	Dried rhizome	Zingiberaceae Martinov	*Curcuma L* .	The International Plant Names Index and World Checklist of Selected Plant Families 2020.<br><br>Pharmacopoeia of China (2015) .<br><br>Govaerts, R. (1999). World Checklist of Seed Plants 3(1, 2a & 2b): 1-1532. MIM, Deurne.
Chi Shao/Bai shao	*Paeonia lactiflora* Pall.	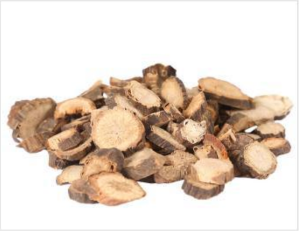	Amur, China North-Central, China South-Central, China Southeast, Chita, Inner Mongolia, Khabarovsk, Manchuria,	Dried root	Paeoniaceae Raf.	*Paeonia* L.	Pharmacopoeia of China (2015) .<br>The International Plant Names Index and World Checklist of Selected Plant Families 2020.<br>Paeonia albiflora Pall. Fl. Ross. 1(2): 92 (1789).

			Mongolia, New York, Primorye				
Danggui	*Angelica sinensis var. wilsonii (H.Wolff) Z.H.Pan & M.F.Watson*<br><br>*Angelica sinensis (Oliv.)Diels*	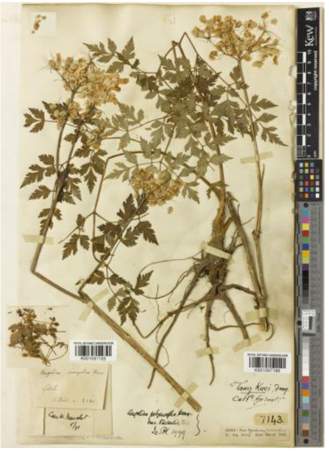	China North-Central, China South-Central, Vietnam	Dried root	*Apiaceae* Lindl.	*Angelica* L.	Chinese Pharmacopoeia Commission (ed.) (2015).<br>The International Plant Names Index and World Checklist of Selected Plant Families 2020.<br><br>Angelica polymorpha var. sinensis Oliv. Hooker's Icon. Pl. 20: t. 1999 (1891).
Sanleng	*Sparganium stoloniferum* (Buch.-Ham. ex Graebn.) Buch.-Ham. ex Juz. [Typhaceae]	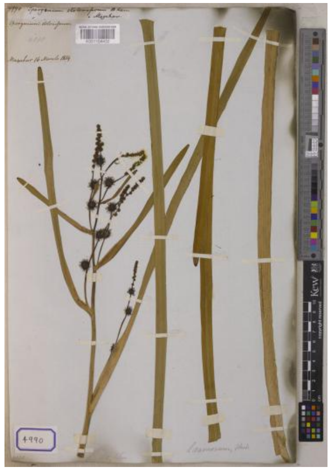	Temp. Asia to Himalaya	Dried rhizome	*Typhaceae* Juss.	*Sparganium* L.	Pharmacopoeia of China (2010).<br>The International Plant Names Index and World Checklist of Selected Plant Families 2020.<br><br>*Sparganium erectum* subsp. stoloniferum (Buch.-Ham. ex Graebn.) H.Hara J. Jap. Bot. 51: 228 (1976).

Yanhusuo	*Corydalis yanhusuo* (Y.H.Chou & Chun C.Hsu) W.T.Wang ex Z.Y.Su & C.Y.Wu [Papaveraceae]	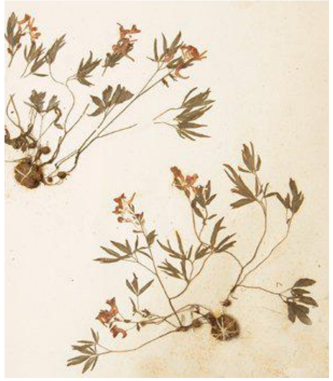	China South-Central, China Southeast	Dried tuber	*Papavera ceae Juss* .	*Corydalis* DC.	The International Plant Names Index and World Checklist of Selected Plant Families 2020.<br><br>Corydalis ternata f. yanhusuo (Y.H.Chou & Chun C.Hsu) Y.C.Zhu in Z.Y.Chang (ed.), Pl. Medic. Chinae Bor.-Orient.: 442 (1989).
Taoren	*Prunus persica* (L.) Batsch	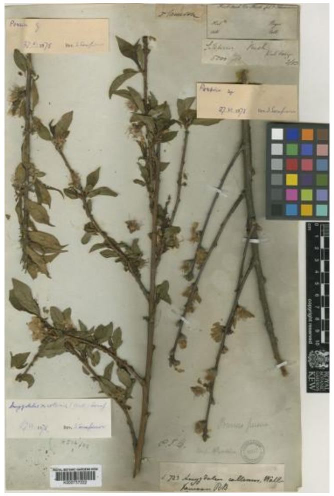<br>*z*	China North-Central	Dried ripe seed	*Rosaceae*	Prunus L.	Pharmacopoeia of China (2015) .<br><br>Amygdalus communis var. persica Risso Hist. Nat. Prod. Eur. Mérid. 2: 327 (1826).

Danshen	*Salvia miltiorrhiza* Bunge<br>[Lamiaceae]	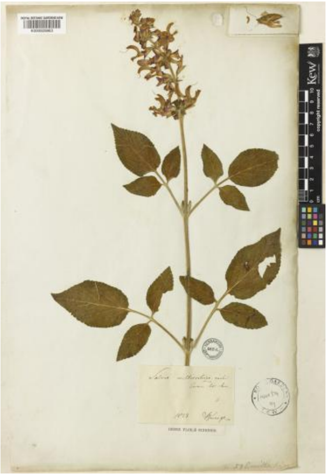	Native to:<br>China North-Central, China South-Central, China Southeast, Vietnam<br><br>Introduced into:<br>Korea	Dried root and rhizome	Lamiaceae Martinov	Salvia L.	Chinese Pharmacopoeia Commission (ed.) (2015).<br><br>Govaerts, R. (2003). World Checklist of Selected Plant Families Database in ACCESS: 1-216203.
Chuanxiong	1.*Conioselinum anthriscoides* (H.Boissieu) Pimenov & Kljuykov [Apiaceae]<br>*2.Ligusticum chuanxiong*<br>*3.Conioselinum anthriscoides* 'Chuanxiong' [Apiaceae]	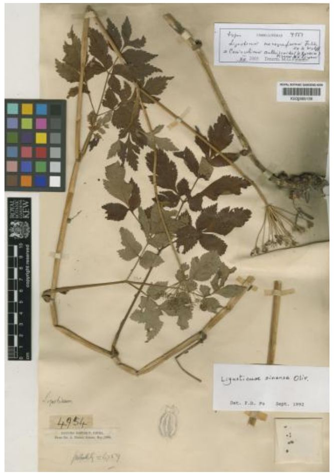	Hebei province in China	Rhizomes	*Apiaceae*	*Ligusticum*	The International Plant Names Index and World Checklist of Selected Plant Families 2020.<br>*Ligusticum chuanxiong S.H.Qiu, Y.Q.Zeng, K.Y.Pan, Y.C.Tang & J.M.Xu Acta Phytotax. Sin. 17(2): 102 (1979).*<br>*Ligusticum sinense Oliv., Hooker's Icon. Pl. 20: t. 1958 (1891).*

Guizhi	*Cinnamomum cassia* (L.) J.Presl [Lauraceae]	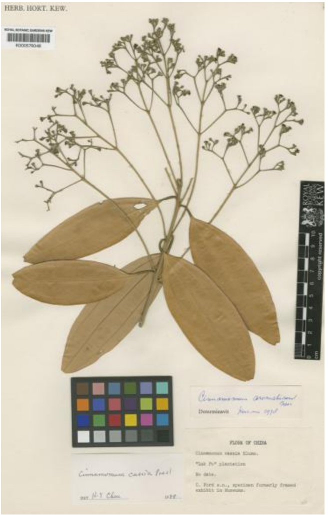	China Southeast	Dried bark,Dried young branch;Dried stem bark	*Lauraceae*	Cinnamomum Schaeff.	The International Plant Names Index and World Checklist of Selected Plant Families 2020.<br>Chinese Pharmacopoeia Commission (ed.) (2015).<br>Kew Royal Botanic Garden.
Puhaung	*Typha angustifolia L.* [Typhaceae]	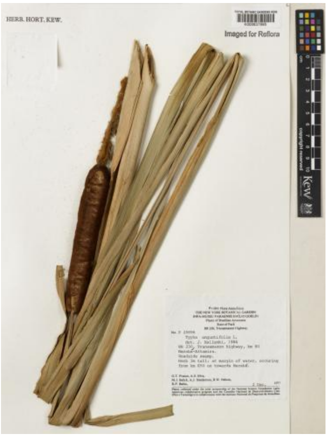	Temp. Northern Hemisphere	Dried pollen	Typhaceae	Typha L.	The International Plant Names Index and World Checklist of Selected Plant Families 2020.<br><br>Charkevicz, S.S. (ed.) (1996). Plantae Vasculares Orientalis Extremi Sovietici 8: 1-382. Nauka, Leningrad.

Huangqi	*1.Astragalus mongholicus* Bunge [Fabaceae]<br>2.*Astragalus membranaceus (Fisch.)* Bunge	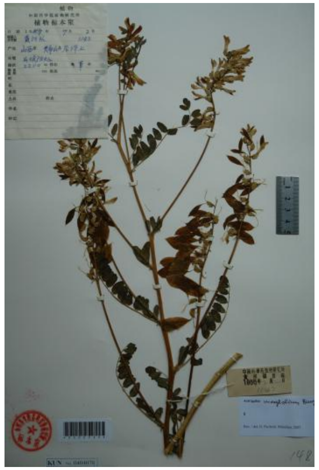	Russian Far East and West & North of China.	Root	*Fabaceae*	*Astragalus* L.	The International Plant Names Index and World Checklist of Selected Plant Families 2020.<br>Medicinal Plants in China (WHO, 1997) .
Xiangfu	*Cyperus rotundus L.*	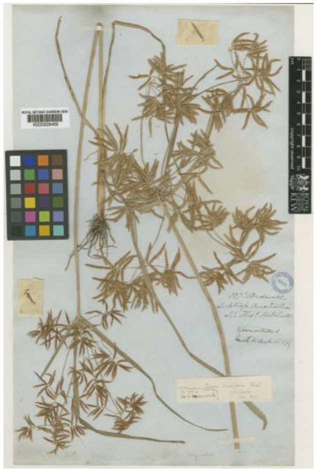	*Tropical & Subtropical Old World.*	Dried rhizome	Cyperaceae Juss.	Cyperus.	The International Plant Names Index and World Checklist of Selected Plant Families 2020.<br>The International Plant Names Index and World Checklist of Selected Plant Families 2020.

Mudanpi	*Paeonia suffruticosa* Andrews [Paeoniaceae]	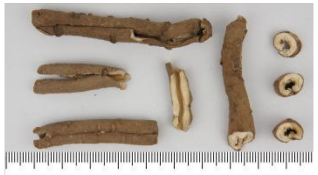	Henan province in China	Dried root bark	Paeoniaceae Raf.	Paeonia	The International Plant Names Index and World Checklist of Selected Plant Families 2020.<br>Pharmacopoeia of China (2010).
Fuling	*Wolfiporia extensa*(Peck) Ginns Poria cocos(Schw.)Wolf	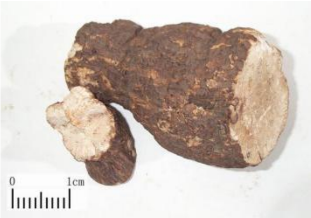	Hebei province in China	Sclerotium	Polyporaceae	Poria Pers.ex Grag	The International Plant Names Index and World Checklist of Selected Plant Families 2020.<br><br>Pharmacopoeia of China (2010).
Gancao	*Glycyrrhiza uralensis* Fisch. ex DC. [Fabaceae]	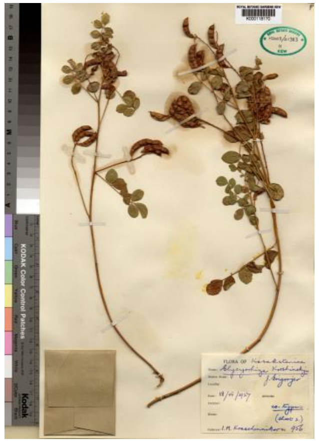	E. European Russia to Mongolia and Pakistan	Dried root and rhizome	*Fabaceae Lindl*	Glycyrrhiza Tourn. ex L.	The International Plant Names Index and World Checklist of Selected Plant Families 2020.<br>Glycyrrhiza asperrima var. uralensis (Fisch. ex DC.) Regel & Herder Bull. Soc. Imp. Naturalistes Moscou 39(1): 566 (1866).

Moyao	*Commiphora myrrha* (T.Nees) Engl.	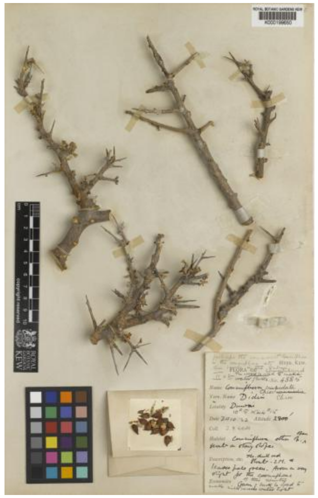	East Africa (Djibouti, Eritrea, Ethiopia, Kenya, Oman, Saudi Arabia, Somalia, Yemen)	Dried oleo-gum	Burseraceae Kunth	Commiphora Jacq.	The International Plant Names Index and World Checklist of Selected Plant Families 2020. *Flora of Tropical East Africa* *Commiphora cuspidata* Chiov. Bull. Misc. Inform. Kew 1941: 134 (1941). Herbs of Commerce (McGuffin et al., 2000)
Honghua	*Carthamus tinctorius* L.[Asteraceae]	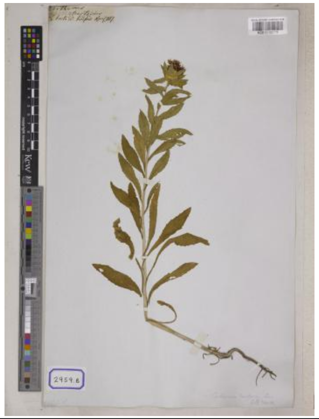	Central & E. Turkey to Iran; China （Iran, Nicaragua, Turkey）	Dried flower	Asteraceae Bercht. & J.Presl	Carthamus L.	The International Plant Names Index and World Checklist of Selected Plant Families 2020. Pharmacopoeia of China (2010).

The validated information of plants, including TCM names,species names/scientific names,botanical documentation, location, used part (used part of medicinal species) and family and genus.
