# Supplementary material for: Modular Characteristics and Mechanism of Action of Herbs for Endometriosis Treatment in Chinese Medicine: A Data Mining and Network Pharmacology–Based Identification
Source: Front Pharmacol. 2020 Mar 6;11:147. doi: 10.3389/fphar.2020.00147 (PMC7069061; doi:10.3389/fphar.2020.00147)
Supplement: Supplementary Table 6 — The major KEGG pathways of major Chinese herbs, herbs pairs and prescription. [file Table_6.pdf]

Table 6:The major KEGG pathways of major Chinese herbs,herbs pairs and prescription

Chinese herbs	KEGG	Term	Count	PValue	FDR
Salvia miltiorrhiza Bunge	hsa04080	Neuroactive ligand-receptor interaction	35	5.13925E-16	7.10543E-13
	hsa04066	HIF-1 signaling pathway	22	2.70133E-15	3.39728E-12
	hsa05200	Pathways in cancer	40	3.3885E-15	4.39648E-12
	hsa04726	Serotonergic synapse	18	5.5652E-10	7.1047E-07
	hsa04020	Calcium signaling pathway	22	7.71595E-10	9.85042E-07
	hsa04151	PI3K-Akt signaling pathway	29	5.16849E-09	6.59825E-06
	hsa04668	TNF signaling pathway	16	2.11219E-08	2.69649E-05
	hsa04725	Cholinergic synapse	16	3.51194E-08	4.48345E-05
	hsa04915	Estrogen signaling pathway	14	4.21996E-07	0.000538731
	hsa04068	FoxO signaling pathway	16	4.43379E-07	0.000566029
	hsa04931	Insulin resistance	14	1.17073E-06	0.001494585
	hsa04370	VEGF signaling pathway	11	1.20367E-06	0.001536624
	hsa04510	Focal adhesion	19	1.25888E-06	0.001607116
	hsa04660	T cell receptor signaling pathway	13	3.17253E-06	0.004050066
	hsa04723	Retrograde endocannabinoid signaling	13	3.5282E-06	0.004504111
Angelica sinensis (Oliv.)Diels	hsa04080	Neuroactive ligand-receptor interaction	21	1.67934E-12	2.08168E-09
	hsa04726	Serotonergic synapse	13	8.2992E-10	1.02876E-06
	hsa04020	Calcium signaling pathway	15	2.12662E-09	2.63615E-06
	hsa05200	Pathways in cancer	20	6.67336E-09	8.27224E-06
	hsa04725	Cholinergic synapse	11	1.4033E-07	0.000173952
	hsa04668	TNF signaling pathway	10	1.12132E-06	0.001389975
	hsa04022	cGMP-PKG signaling pathway	11	3.72905E-06	0.004622404
	hsa04915	Estrogen signaling pathway	9	6.26556E-06	0.007766465
	hsa04723	Retrograde endocannabinoid signaling	9	7.27782E-06	0.009021166
	hsa04024	cAMP signaling pathway	11	2.74002E-05	0.033959789
	hsa04370	VEGF signaling pathway	7	3.19229E-05	0.039564224
	hsa04210	Apoptosis	7	3.50619E-05	0.04345381
	hsa04066	HIF-1 signaling pathway	8	4.83999E-05	0.059979593
	hsa04115	p53 signaling pathway	7	5.46892E-05	0.067771281
	hsa04151	PI3K-Akt signaling pathway	13	0.000159632	0.197698586
Corydalis yanhusuo (Y.H.Chou & Chun C.Hsu) W.T.Wang ex Z.Y.Su & C.Y.Wu	hsa05200	Pathways in cancer	40	2.14492E-17	2.71614E-14
	hsa04066	HIF-1 signaling pathway	19	4.80639E-13	6.08613E-10
	hsa04080	Neuroactive ligand-receptor interaction	26	2.47355E-10	3.13229E-07
	hsa04020	Calcium signaling pathway	21	4.41371E-10	5.58914E-07

	hsa04915	Estrogen signaling pathway	16	1.10938E-09	1.40482E-06
	hsa04668	TNF signaling pathway	16	3.37856E-09	4.27831E-06
	hsa04370	VEGF signaling pathway	12	3.15849E-08	3.99963E-05
	hsa04726	Serotonergic synapse	15	4.64401E-08	5.88078E-05
	hsa00982	Drug metabolism - cytochrome P450	12	1.02459E-07	0.000129745
	hsa04151	PI3K-Akt signaling pathway	25	1.02649E-07	0.000129986
	hsa04022	cGMP-PKG signaling pathway	17	1.13693E-07	0.000143971
	hsa04725	Cholinergic synapse	14	3.48546E-07	0.000441368
	hsa04210	Apoptosis	11	4.11799E-07	0.000521465
	hsa04660	T cell receptor signaling pathway	13	7.72048E-07	0.00097765
	hsa04068	FoxO signaling pathway	14	3.06662E-06	0.003883227
Conioselinum anthriscoides (H.Boissieu) Pimenov & Kljuykov/*Ligusticum chuanxiong*	hsa04080	Neuroactive ligand-receptor interaction	22	6.17748E-12	7.68445E-09
	hsa04020	Calcium signaling pathway	15	2.16604E-08	2.69443E-05
	hsa04022	cGMP-PKG signaling pathway	12	2.57042E-06	0.003197401
	hsa04726	Serotonergic synapse	10	6.49425E-06	0.008078168
	hsa04024	cAMP signaling pathway	10	0.000588903	0.73009854
	hsa05200	Pathways in cancer	14	0.000771833	0.955887841
	hsa00350	Tyrosine metabolism	5	0.000776112	0.96116457
	hsa04370	VEGF signaling pathway	6	0.000793437	0.982522821
	hsa04728	Dopaminergic synapse	8	0.000841631	1.041914593
	hsa04066	HIF-1 signaling pathway	7	0.00100332	1.240934449
	hsa03320	PPAR signaling pathway	6	0.001217354	1.503818297
	hsa00982	Drug metabolism - cytochrome P450	6	0.001301675	1.607207645
	hsa04725	Cholinergic synapse	7	0.002123287	2.609405232
	hsa04727	GABAergic synapse	6	0.003492024	4.258159309
	hsa04911	Insulin secretion	6	0.003492024	4.258159309
Astragalus mongholicus Bunge	hsa05200	Pathways in cancer	52	1.50644E-20	1.93265E-17
	hsa04066	HIF-1 signaling pathway	22	2.69005E-13	3.45113E-10
	hsa04080	Neuroactive ligand-receptor interaction	31	3.50048E-10	4.49087E-07
	hsa04919	Thyroid hormone signaling pathway	20	6.77729E-10	8.69479E-07
	hsa04668	TNF signaling pathway	19	1.44188E-09	1.84983E-06
	hsa04020	Calcium signaling pathway	23	9.42303E-09	1.20891E-05
	hsa04068	FoxO signaling pathway	20	9.58061E-09	1.22913E-05
	hsa05213	Endometrial cancer	13	2.18218E-08	2.79958E-05
	hsa04915	Estrogen signaling pathway	16	1.51694E-07	0.000194613
	hsa04151	PI3K-Akt signaling pathway	30	2.23329E-07	0.000286515

	hsa04115	p53 signaling pathway	13	4.30427E-07	0.000552207
	hsa00982	Drug metabolism - cytochrome P450	13	5.09341E-07	0.000653447
	hsa04660	T cell receptor signaling pathway	15	1.07921E-06	0.001384537
	hsa04370	VEGF signaling pathway	12	1.23194E-06	0.001580481
	hsa00980	Metabolism of xenobiotics by cytochrome P450	13	1.31466E-06	0.001686608
	hsa04510	Focal adhesion	21	2.20044E-06	0.002822974
	hsa04620	Toll-like receptor signaling pathway	15	2.21035E-06	0.00283569
	hsa04022	cGMP-PKG signaling pathway	18	3.27398E-06	0.004200199
	hsa04726	Serotonergic synapse	15	3.86206E-06	0.004954645
	hsa04725	Cholinergic synapse	15	3.86206E-06	0.004954645
Carthamus tinctorius L.	hsa00010	Glycolysis / Gluconeogenesis	7	2.48035E-05	0.030741861
	hsa05200	Pathways in cancer	14	3.01438E-05	0.0373596
	hsa00140	Steroid hormone biosynthesis	6	0.000148329	0.183712192
	hsa04520	Adherens junction	6	0.000385917	0.47732995
	hsa04510	Focal adhesion	9	0.000424709	0.525194639
	hsa00980	Metabolism of xenobiotics by cytochrome P450	6	0.000467868	0.578421821
	hsa05213	Endometrial cancer	5	0.001102507	1.358107623
	hsa04068	FoxO signaling pathway	7	0.001126392	1.387340599
	hsa04910	Insulin signaling pathway	7	0.001311837	1.614044035
	hsa04370	VEGF signaling pathway	5	0.002002738	2.454445789
	hsa04668	TNF signaling pathway	6	0.002470165	3.019268066
	hsa03320	PPAR signaling pathway	5	0.002827573	3.449118272
	hsa00982	Drug metabolism - cytochrome P450	5	0.002984589	3.637404492
	hsa01100	Metabolic pathways	21	0.003144021	3.828243329
	hsa04014	Ras signaling pathway	8	0.003629344	4.407032342
Cyperus rotundus L.	hsa05200	Pathways in cancer	56	8.87937E-23	1.04768E-19
	hsa04370	VEGF signaling pathway	17	1.10289E-08	1.30131E-05
	hsa04660	T cell receptor signaling pathway	20	1.30955E-08	1.54514E-05
	hsa04620	Toll-like receptor signaling pathway	19	2.61626E-08	3.08693E-05
	hsa04020	Calcium signaling pathway	25	2.80035E-08	3.30413E-05
	hsa04510	Focal adhesion	26	9.12014E-08	0.000107608
	hsa04080	Neuroactive ligand-receptor interaction	29	2.32927E-07	0.00027483
	hsa04662	B cell receptor signaling pathway	15	5.50572E-07	0.000649618
	hsa04210	Apoptosis	16	6.29135E-07	0.000742314
	hsa04115	p53 signaling pathway	14	1.06693E-06	0.00125887
	hsa04540	Gap junction	15	4.70048E-06	0.005545963
	hsa04621	NOD-like receptor signaling pathway	12	1.57121E-05	0.018537187
	hsa04010	MAPK signaling pathway	26	1.84686E-05	0.021788895
	hsa04910	Insulin signaling pathway	17	4.02143E-05	0.04743861
	hsa04062	Chemokine signaling pathway	20	6.65876E-05	0.078538465

	hsa00980	Metabolism of xenobiotics by cytochrome P450	11	6.76579E-05	0.079800451
	hsa04722	Neurotrophin signaling pathway	15	0.00020885	0.246143993
	hsa04912	GnRH signaling pathway	13	0.000279296	0.329045529
	hsa04110	Cell cycle	14	0.000783437	0.920477313
	hsa04270	Vascular smooth muscle contraction	13	0.000955826	1.121977695
Commiphora myrrha (T.Nees) Engl.	hsa05200	Pathways in cancer	54	3.82947E-24	4.87468E-21
	hsa04066	HIF-1 signaling pathway	22	4.59844E-14	5.85088E-11
	hsa04668	TNF signaling pathway	22	4.50669E-13	5.73641E-10
	hsa04620	Toll-like receptor signaling pathway	21	3.6518E-12	4.64845E-09
	hsa05213	Endometrial cancer	15	4.58253E-11	5.83329E-08
	hsa04151	PI3K-Akt signaling pathway	34	1.01539E-10	1.29253E-07
	hsa00982	Drug metabolism - cytochrome P450	16	2.01311E-10	2.56256E-07
	hsa04510	Focal adhesion	24	4.94918E-09	6.3E-06
	hsa00980	Metabolism of xenobiotics by cytochrome P450	15	7.03664E-09	8.95721E-06
	hsa04660	T cell receptor signaling pathway	17	7.30844E-09	9.3032E-06
	hsa04726	Serotonergic synapse	17	3.41885E-08	4.35199E-05
	hsa04915	Estrogen signaling pathway	16	4.70904E-08	5.99432E-05
	hsa04370	VEGF signaling pathway	13	5.52325E-08	7.03076E-05
	hsa04080	Neuroactive ligand-receptor interaction	26	7.80601E-08	9.93657E-05
	hsa04068	FoxO signaling pathway	18	8.71687E-08	0.00011096
Curcuma phaeocaulis Valeton and *Sparganium stoloniferum*	hsa04080	Neuroactive ligand-receptor interaction	28	1.94743E-15	2.5091E-12
	hsa05200	Pathways in cancer	28	9.8702E-12	1.23814E-08
	hsa04020	Calcium signaling pathway	17	7.04878E-09	8.84213E-06
	hsa04210	Apoptosis	11	2.04983E-08	2.57135E-05
	hsa04022	cGMP-PKG signaling pathway	14	5.4194E-07	0.000679818
	hsa04726	Serotonergic synapse	12	6.74668E-07	0.000846314
	hsa04919	Thyroid hormone signaling pathway	12	9.65275E-07	0.001210853
	hsa04071	Sphingolipid signaling pathway	12	1.47992E-06	0.001856432
	hsa04725	Cholinergic synapse	11	5.35093E-06	0.006712102
	hsa04115	p53 signaling pathway	8	5.72328E-05	0.071770307
	hsa04668	TNF signaling pathway	9	0.000184372	0.231034015
	hsa04727	GABAergic synapse	8	0.00026067	0.326497821
	hsa04370	VEGF signaling pathway	7	0.000277529	0.347580698
	hsa00982	Drug metabolism - cytochrome P450	7	0.00050277	0.628857232
	hsa04066	HIF-1 signaling pathway	8	0.000550383	0.688222361
Guizhi Fuling Wan	hsa01100	Metabolic pathways	137	1.21758E-18	1.59342E-15
	hsa00260	Glycine, serine and threonine metabolism	20	1.84268E-14	2.4114E-11
	hsa00330	Arginine and proline metabolism	22	3.10796E-14	4.06786E-11

hsa05200	Pathways in cancer	61	1.25593E-13	1.64324E-10
hsa04066	HIF-1 signaling pathway	28	6.20439E-13	8.11895E-10
hsa04620	Toll-like receptor signaling pathway	28	8.24479E-12	1.07897E-08
hsa04668	TNF signaling pathway	26	3.74931E-10	4.90662E-07
hsa00980	Metabolism of xenobiotics by cytochrome P450	20	9.62318E-09	1.25936E-05
hsa00982	Drug metabolism - cytochrome P450	19	1.40912E-08	1.84408E-05
hsa04370	VEGF signaling pathway	17	1.04849E-07	0.000137214
hsa04660	T cell receptor signaling pathway	21	3.52102E-07	0.000460785
hsa05213	Endometrial cancer	15	4.6925E-07	0.000614094
hsa04662	B cell receptor signaling pathway	17	6.59147E-07	0.000862605
hsa04210	Apoptosis	16	8.25757E-07	0.001080641
hsa04010	MAPK signaling pathway	34	2.5499E-06	0.003336939
hsa04068	FoxO signaling pathway	23	3.13325E-06	0.004100332
hsa04064	NF-kappa B signaling pathway	18	3.77638E-06	0.004941942
hsa04014	Ras signaling pathway	31	5.18219E-06	0.006781586
hsa04080	Neuroactive ligand-receptor interaction	35	7.04081E-06	0.009213734
hsa04726	Serotonergic synapse	20	7.95857E-06	0.010414675
