# Supplementary material for: Modular Characteristics and Mechanism of Action of Herbs for Endometriosis Treatment in Chinese Medicine: A Data Mining and Network Pharmacology–Based Identification
Source: Front Pharmacol. 2020 Mar 6;11:147. doi: 10.3389/fphar.2020.00147 (PMC7069061; doi:10.3389/fphar.2020.00147)
Supplement: Supplementary Table 8 — The GO enrichment of endometriosis-associated genes that coincided with Chinese herbs. [file Table_8.pdf]

Table 8:The GO enrichment of endometriosis-associated genes that coincided with Chinese herbs

Category	GO term	term	Count	PValue	FDR
GOTERM_BP	GO:0042127	regulation of cell proliferation	70	5.23963E-42	9.39815E-39
	GO:0042981	regulation of apoptosis	68	3.12277E-39	5.60121E-36
	GO:0043067	regulation of programmed cell death	68	5.8644E-39	1.05188E-35
	GO:0010941	regulation of cell death	68	7.41403E-39	1.32983E-35
	GO:0043066	negative regulation of apoptosis	45	2.37012E-32	4.2512E-29
	GO:0043069	negative regulation of programmed cell death	45	4.36873E-32	7.83604E-29
	GO:0060548	negative regulation of cell death	45	4.9313E-32	8.84511E-29
	GO:0010033	response to organic substance	56	1.24784E-29	2.23821E-26
	GO:0009611	response to wounding	46	6.26694E-26	1.12408E-22
	GO:0008284	positive regulation of cell proliferation	41	4.21058E-25	7.55238E-22
	GO:0044093	positive regulation of molecular function	47	4.54496E-25	8.15215E-22
	GO:0006916	anti-apoptosis	30	7.11061E-23	1.27541E-19
	GO:0009719	response to endogenous stimulus	38	2.34188E-22	4.20055E-19
	GO:0009891	positive regulation of biosynthetic process	47	5.65958E-22	1.01514E-18
	GO:0010604	positive regulation of macromolecule metabolic process	51	1.51892E-21	2.72444E-18
	GO:0051173	positive regulation of nitrogen compound metabolic process	45	1.55928E-21	2.79683E-18
	GO:0031328	positive regulation of cellular biosynthetic process	46	2.37593E-21	4.26163E-18
	GO:0048545	response to steroid hormone stimulus	28	2.5147E-21	4.51054E-18
	GO:0009725	response to hormone stimulus	35	9.4908E-21	1.70233E-17
	GO:0042592	homeostatic process	47	1.3311E-20	2.38754E-17
GOTERM_CC	GO:0005615	extracellular space	45	1.02009E-20	1.34353E-17
	GO:0044421	extracellular region part	49	3.81139E-18	5.01987E-15
	GO:0005829	cytosol	47	2.21086E-11	2.91185E-08
	GO:0005576	extracellular region	59	3.40949E-11	4.49054E-08
	GO:0000267	cell fraction	41	9.39912E-11	1.23793E-07
	GO:0005792	microsome	18	4.09607E-09	5.39481E-06
	GO:0042598	vesicular fraction	18	6.35774E-09	8.37359E-06
	GO:0009986	cell surface	20	4.17004E-08	5.49224E-05
	GO:0031974	membrane-enclosed lumen	50	4.41651E-08	5.81685E-05
	GO:0043233	organelle lumen	48	1.84626E-07	0.000243165
	GO:0005626	insoluble fraction	30	2.47206E-07	0.000325587
	GO:0045121	membrane raft	12	1.2791E-06	0.001684647
	GO:0043005	neuron projection	17	3.96883E-06	0.005227098
	GO:0005624	membrane fraction	27	4.37781E-06	0.005765732

	GO:0009897	external side of plasma membrane	12	6.88722E-06	0.009070574
	GO:0070013	intracellular organelle lumen	43	1.08008E-05	0.014224484
	GO:0030141	secretory granule	12	1.18833E-05	0.015649966
	GO:0005625	soluble fraction	15	2.70641E-05	0.035639501
	GO:0030425	dendrite	11	2.89583E-05	0.038133391
	GO:0042995	cell projection	23	3.43852E-05	0.045278286
GOTERM_MF	GO:0020037	heme binding	18	1.80985E-13	2.62945E-10
	GO:0046906	tetrapyrrole binding	18	5.31786E-13	7.72693E-10
	GO:0046983	protein dimerization activity	30	3.94059E-11	5.72558E-08
	GO:0042802	identical protein binding	31	4.38211E-10	6.36711E-07
	GO:0005506	iron ion binding	21	2.13794E-09	3.10639E-06
	GO:0005496	steroid binding	11	7.01757E-09	1.01964E-05
	GO:0008289	lipid binding	24	1.22367E-08	1.77796E-05
	GO:0004879	ligand-dependent nuclear receptor activity	10	4.34274E-08	6.30991E-05
	GO:0019899	enzyme binding	25	4.52794E-08	6.57899E-05
	GO:0016209	antioxidant activity	9	1.18471E-07	0.000172136
	GO:0005125	cytokine activity	15	1.84668E-07	0.000268318
	GO:0009055	electron carrier activity	15	8.48571E-07	0.001232948
	GO:0019825	oxygen binding	8	1.0339E-06	0.001502218
	GO:0016712	oxidoreductase activity, acting on paired donors, with incorporation or reduction of molecular oxyge	7	1.77422E-06	0.00257787
	GO:0046982	protein heterodimerization activity	14	2.46668E-06	0.003583971
	GO:0003707	steroid hormone receptor activity	8	2.58523E-06	0.003756216
	GO:0008083	growth factor activity	12	6.44052E-06	0.009357533
	GO:0070330	aromatase activity	6	1.34945E-05	0.019605479
	GO:0042803	protein homodimerization activity	16	2.32E-05	0.033703766
	GO:0004601	peroxidase activity	6	4.75997E-05	0.069139119
