# Supplementary figures and images for: Modular Characteristics and Mechanism of Action of Herbs for Endometriosis Treatment in Chinese Medicine: A Data Mining and Network Pharmacology–Based Identification
Source: Front Pharmacol. 2020 Mar 6;11:147. doi: 10.3389/fphar.2020.00147 (PMC7069061; doi:10.3389/fphar.2020.00147)

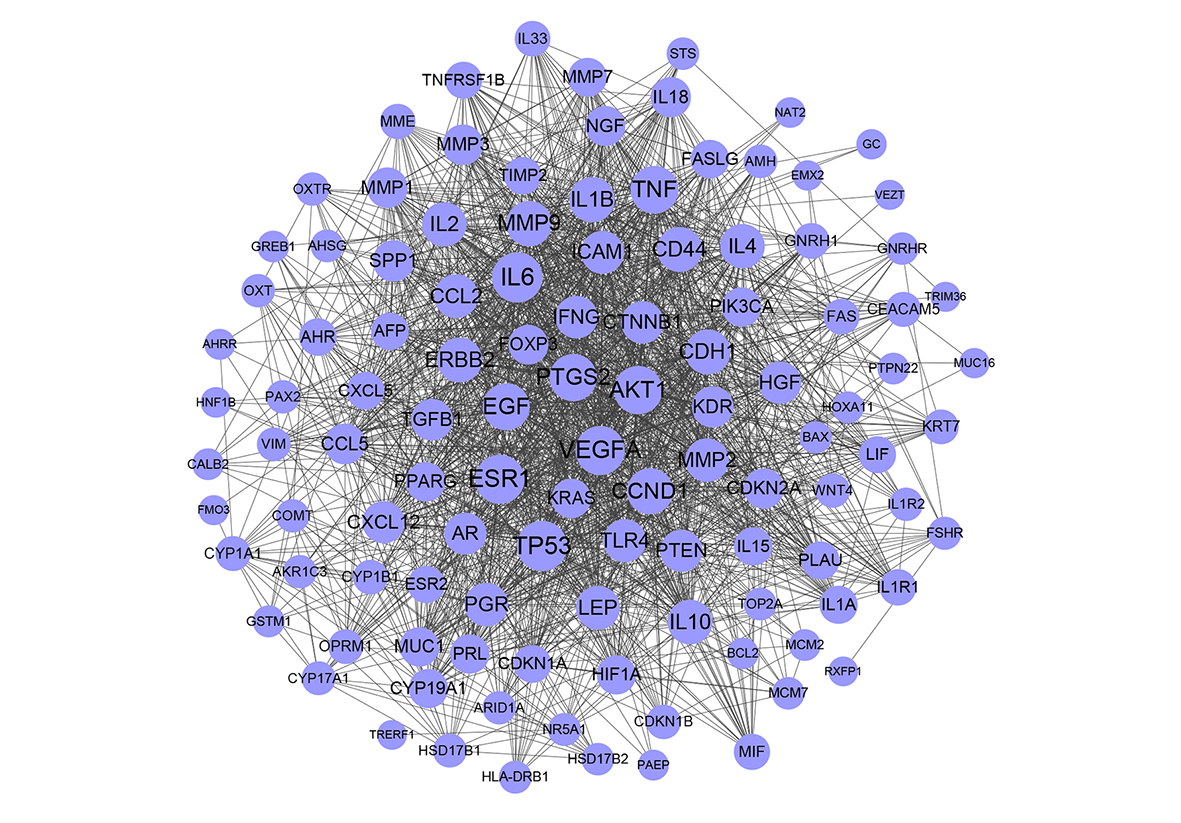

Supplement: Supplementary Figure 1 — The PPI network of the major endometriosis genes. [file Image_1.tif]

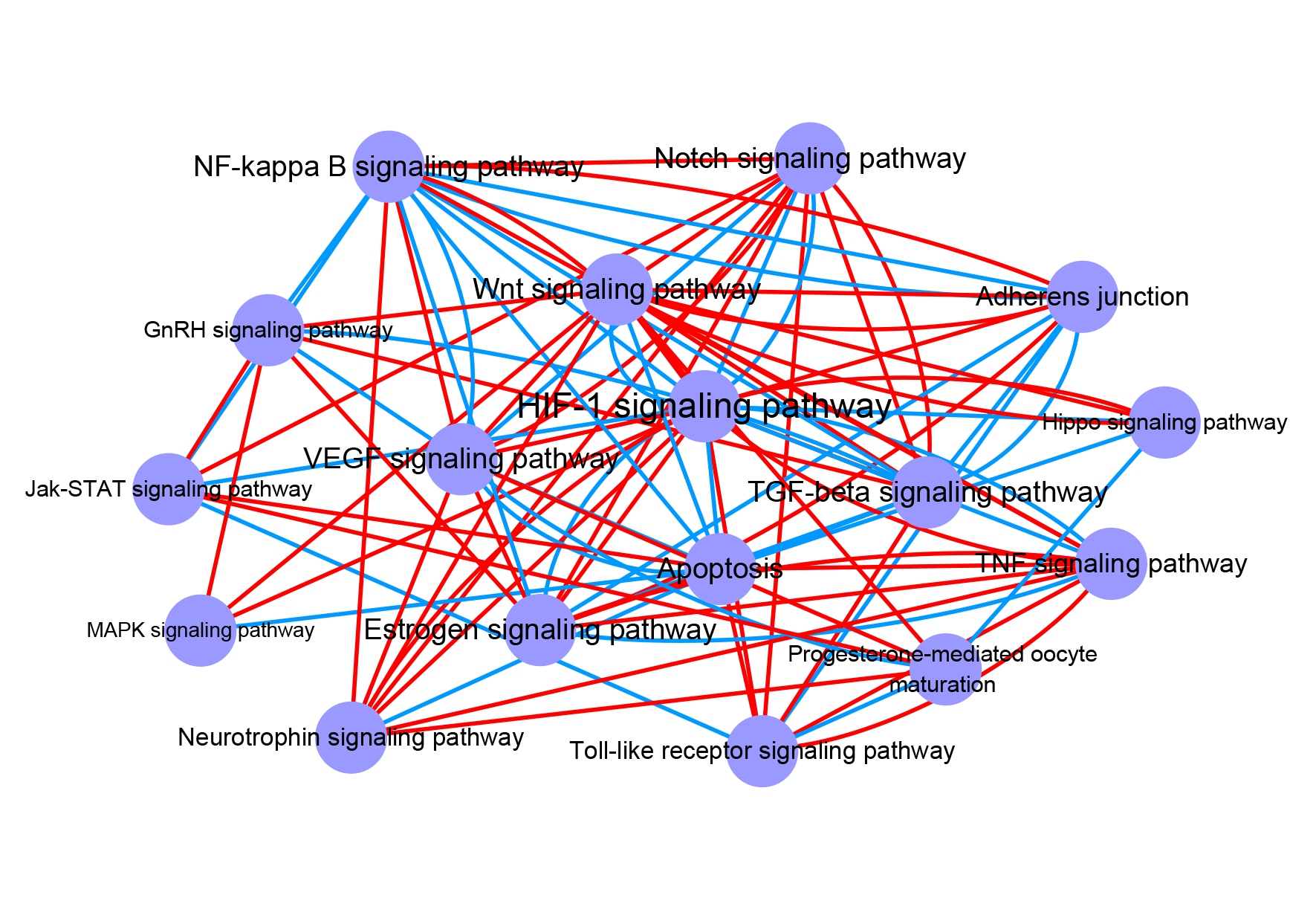

Supplement: Supplementary Figure 2 — The cross-talk pathways network of Chinese herbs in endometriosis treatment. [file Image_2.tif]
